# Reduced human activity during COVID-19 alters avian land use across North America

**DOI:** 10.1126/sciadv.abf5073

**Published:** 2021-09-22

**Authors:** Michael B. Schrimpf, Paulson G. Des Brisay, Alison Johnston, Adam C. Smith, Jessica Sánchez-Jasso, Barry G. Robinson, Miyako H. Warrington, Nancy A. Mahony, Andrew G. Horn, Matthew Strimas-Mackey, Lenore Fahrig, Nicola Koper

**Affiliations:** 1 Natural Resources Institute, University of Manitoba, Winnipeg, Canada.; 2 Environment and Climate Change Canada, Edmonton, Winnipeg, and Ottawa, Canada.; 3 Cornell Lab of Ornithology, 159 Sapsucker Woods Road, Ithaca, NY, USA.; 4 Dalhousie University, Halifax, Canada.; 5 Geomatics and Landscape Ecology Research Laboratory, Carleton University, Ottawa, Canada.

## Abstract

The COVID-19 pandemic resulted in extraordinary declines in human mobility, which, in turn, may affect wildlife. Using records of more than 4.3 million birds observed by volunteers from March to May 2017–2020 across Canada and the United States, we found that counts of 66 (80%) of 82 focal bird species changed in pandemic-altered areas, usually increasing in comparison to prepandemic abundances in urban habitat, near major roads and airports, and in counties where lockdowns were more pronounced or occurred at the same time as peak bird migration. Our results indicate that human activity affects many of North America’s birds and suggest that we could make urban spaces more attractive to birds by reducing traffic and mitigating the disturbance from human transportation after we emerge from the pandemic.

## Teaser

Bird counts increased in locations with reduced human traffic resulting from the COVID-19 pandemic.

## INTRODUCTION

In response to the threats posed by the coronavirus disease 2019 (COVID-19) pandemic, human movements were restricted to a degree unprecedented in modern times (*1*, *2*). This “anthropause” (*3*) may have had numerous impacts on other species. Urban wildlife may have been particularly affected, as markedly decreased vehicular traffic resulted in decreased air pollution, anthropogenic noise, and risks of wildlife collisions with vehicles (*4*–*6*). Many bird species are negatively affected by roads (*7*–*9*), so urban birds may have temporarily benefited from decreased traffic [e.g., (*10*)]. Conversely, some species benefit from human disturbances, such as anthropogenic noise, which can reduce interspecific competition and predation rates (*11*), and thus, the anthropause may have had negative consequences for some species. Understanding these interactions could ultimately help us to develop effective strategies to reduce our environmental footprint in a post–COVID-19 world (*12*).

**Fig. 1.**
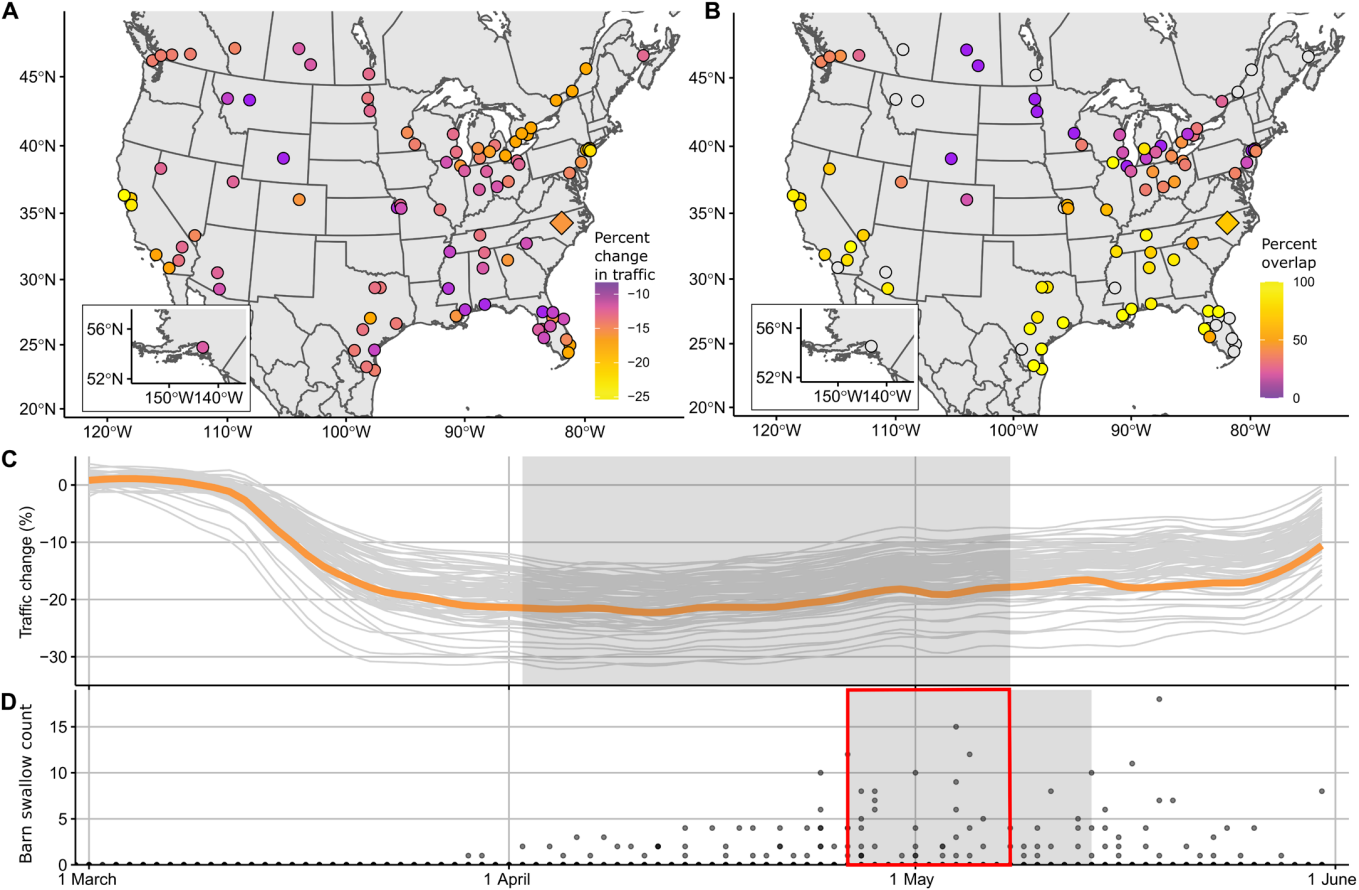
Change in traffic during COVID-19 pandemic travel restrictions and temporal overlap with migration. (**A**) Change in traffic volume in 93 counties in North America, calculated from (*31*) as the inverse of the time cell phones spent at home during the peak pandemic lockdown period. (**B**) Example map showing percent overlap between the peak lockdown period and peak migration of an example species, barn swallow (*H. rustica*). Gray circles are counties in which barn swallows were absent from >95% of the checklists and were therefore not included in that model. (**C**) Changes in human mobility (travel away from home) varied among counties (gray lines). Orange line shows an example county [Wake, North Carolina; located at diamond, maps (A) and (B)], and peak lockdown period (25th to 75th percentile of cumulative total mobility reduction) for this county is shown using gray rectangle. (**D**) Example of typical migratory pattern (here, for barn swallow) with the peak period in gray rectangle showing 25th to 75th percentile of the cumulative total counts of this species. Overlap between peak abundance and peak travel restriction is the percent of the shaded region in (D) that overlaps the shaded region in (C), indicated by the red outline.

We used data from one of the world’s largest biodiversity community science programs, eBird (*13*, *14*), to examine whether reported bird abundances in cities and surrounding rural areas across Canada and the United States changed during the 2020 spring migration season (March to May). This 3-month period coincided with the largest decreases in human mobility due to pandemic travel restrictions in this region. We compared the recorded abundances of 82 bird species from March to May before (2017–2019) and during (2020) pandemic travel restrictions, among and within 93 counties in Canada and the United States that contained urban areas (Fig. 1A). Because of potential effects of the pandemic on the behavior of volunteer observers (*15*), we subsampled the available eBird surveys (termed “checklists”) to ensure that there were no differences in sampling effort, sampling location, or other sampling attributes in the prepandemic and pandemic periods (see Materials and Methods and figs. S1 to S4).

We tested whether bird responses to the pandemic differed across locations with varying levels of reduced human activity during the pandemic lockdowns. Because bird populations and densities naturally fluctuate among years, we did not consider increased or decreased bird counts in 2020 per se to indicate an effect of travel restrictions. Instead, we focused on understanding whether changes occurred in the relationships between bird counts and five different indices of human activity that underwent pandemic-related changes [described below, (i) to (v), and in table S1]. Examining interactions between these indices and the prepandemic versus pandemic periods allowed us to establish whether abundances changed more in areas where the pandemic’s effects were greater. For example, if differences in bird observations among years were caused by decreased air and land vehicular traffic, we expected that bird abundances would change more when close to major roads and airports. This approach also allowed us to statistically control for regional variability in lockdowns; for example, counties included in our analysis had traffic reductions, ranging from 8 to 25% relative to baselines.

We measured human traffic reductions across counties during March to May 2020, using the best available mobility data [(i); “change in traffic”; Fig. 1A]. In addition, we tested whether bird responses to the pandemic were stronger for migrating birds that encountered counties primarily during the height of lockdown versus before or after the change in traffic had peaked. For this, we calculated the county- and species-specific degree of overlap between peak lockdown and peak bird migration period [(ii); “migration overlap”; Fig. 1, B to D]. Nonzero interactions at the scales of these two indices suggested that birds altered their distributions at scales of hundreds to thousands of kilometers in response to variable levels of change in average vehicular traffic and human activity.

The comparisons above were made across counties, which is the finest spatial scale at which we could obtain data on declines in human mobility due to COVID-19 lockdowns. However, we also expected that counts of birds might change in response to declining human activity within counties, particularly in locations where the reduction in human activity was strongest. Therefore, we assessed whether the pandemic resulted in changes to the relationships between bird counts and three other indices that acted as proxies of human activity within counties: (iii) distance to major roads, (iv) distance to international airports, and (v) urban versus rural sites. We tested for interaction effects on bird counts between time period (prepandemic versus pandemic) and each of these three variables. We assumed that declines in human activity caused by pandemic lockdowns were greater in the vicinity of major roads and airports, and in urban compared with rural areas, and thus predicted that relationships between bird counts and the pandemic would be influenced by these proxies of human activity. For example, if a species avoided traffic before the pandemic, we predicted that the slope of the relationship between bird counts and distance to a major road would become shallower (less negative) during the pandemic than before the pandemic. Any nonzero interaction terms at this within-county scale suggested that, during the pandemic, birds present in a county altered their use of areas within roughly 1 to 100 km of landscape features associated with human activity.

## RESULTS AND DISCUSSION

**Fig. 2.**
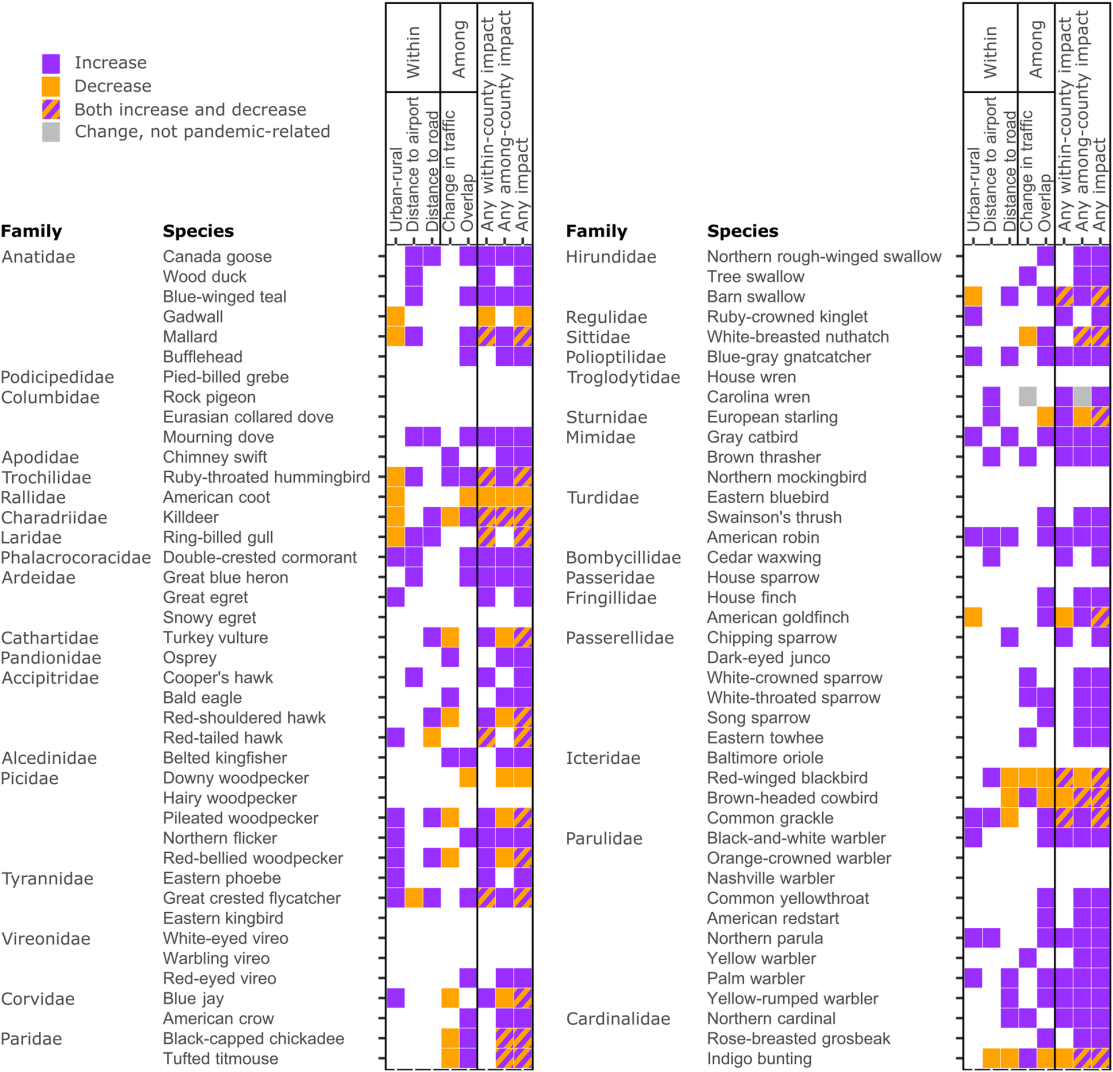
Species-specific responses to declines in human activity during the pandemic. Color matrix indicates whether 82 bird species showed increased (purple), decreased (orange), or both (hashed) counts in response to declining human activity (March to May 2020) in the United States and Canada compared with the same areas before the pandemic (March to May 2017–2019) based on whether 95% credible intervals of coefficient posterior distributions for statistical interactions between each variable and the pandemic/prepandemic period did not overlap zero. Indices of decreased human activity include sites closer to major roads, international airports, or urban areas, or counties with greater traffic reductions or greater overlap between peak lockdown and peak migration period. White squares indicate no demonstrable change in bird counts, and gray indicates a nonlinear trend such that counts changed slightly in counties with intermediate levels of travel restrictions.

A total of 4,353,739 observations of individual birds from 88,846 checklists from the eBird database were selected for the analysis after filtering and subsampling all available checklists to minimize the possibility that results were affected by changes in birder behavior during the pandemic. Of 82 focal species, 80% (66 species) showed a change in relationship with at least one index of reduced human activity during 2020 (Fig. 2 and Table 1). Species were 14 times more likely to consistently show increased rather than decreased counts when considering all indices, and a species’ response relative to any particular index was 2.7 times more likely to be an increase rather than a decrease. One-quarter (26%) of species showed both increasing and decreasing responses to reduced human activity, depending on which variable was evaluated; for example, red-tailed hawks (*Buteo jamaicensis*) were more commonly observed in urban habitats during the pandemic but were less commonly seen close to major roads (Fig. 2). Such mixed results varied greatly among species, suggesting that behavioral adaptations or plasticity have enabled some species to cope with or even benefit from certain types of anthropogenic disturbances, but not others. For example, many species can adjust their vocalizations to communicate more effectively in the presence of anthropogenic noise (*16*), but they may not be able to also adapt to changes to the physical environment within urban landscapes. Future research should assess whether the effects of lockdowns on birds were likely caused by decreased noise, traffic mortality, air pollution, or other changes and would help us better understand why counts of some species increased in relation to some measures of human activity but decreased in relation to others. Nonetheless, it is clear that the effects of lockdowns were strongly biased toward increasing the number of birds counted in human-altered areas when activity and traffic declined.

**Table 1.** Summary of species responses by predictor variable. The number (percent) of 82 focal species whose counts changed in relation to declining human activity during March to May 2020 compared with the prepandemic baseline period (March to May 2017–2019). Responses at among- and within-county scales are summarized by the number (percent) of species with only increases or decreases, any examples of increases or decreases, and a mix of increases and decreases per species among variables at that scale.

	**Increase** **(only)**	**Decrease** **(only)**	**Ratio of** **increase** **(only):decrease** **(only)**	**Mixed** **response**	**Increase** **(any)**	**Decrease** **(any)**	**Ratio of** **increase** **(any):decrease (any)**	**Any response**
Among-county								
Change in traffic	14 (17.1)	10 (12.2)	1.4					
Overlap	35 (42.7)	6 (7.3)	5.8					
Any among- county variable	39 (47.6)	9 (11.0)	4.3	6 (7.3)	45 (54.9)	15 (18.3)	3.0	54 (65.9)
Within-county								
Urban-rural	17 (20.7)	8 (9.8)	2.1					
Distance to airport	18 (22.0)	2 (2.4)	9.0					
Distance to road	17 (20.7)	5 (6.1)	3.4					
Any within- county variable	29 (35.4)	5 (6.1)	5.8	9 (11.0)	38 (46.3)	14 (17.1)	2.7	43 (52.4)
Any variable	42 (51.2)	3 (3.7)	14.0	21 (25.6)	63 (76.8)	24 (29.3)	2.7	66 (80.5)

**Fig. 3.**
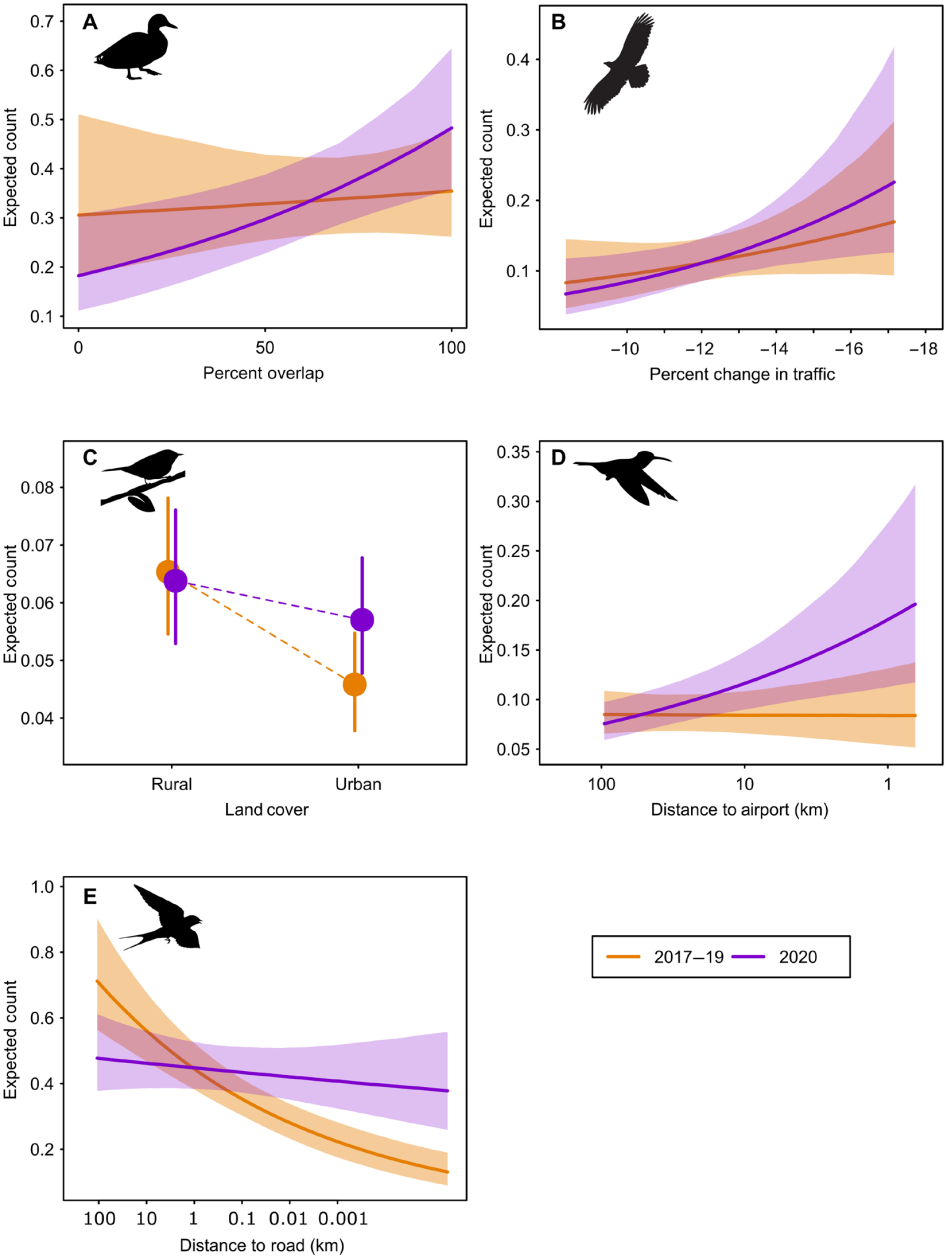
Examples of interactions between the pandemic and prepandemic periods and variables indexing declines in human activity. Marginal predicted mean count (lines or points) including 95% credible interval (shaded areas or error bars) of the effect of the predictor on bird counts during the COVID-19 pandemic (purple) compared to the prepandemic baseline period (orange) for 5 of 82 North American focal species: (**A**) overlap between peak migration and peak travel reductions, blue-winged teal (*Anas discors*); (**B**) percent change in human mobility during pandemic, bald eagle (*H. leucocephalus*); (**C**) urban versus rural habitat, black-and-white warbler (*Mniotilta varia*); (**D**) distance to airport, ruby-throated hummingbird (*A. colubris*); and (**E**) distance to major road, barn swallow (*H. rustica*). Changes to human activity during the pandemic increase from left to right on each panel. The *y* axis shows the mean expected bird count, scaled to a single representative county.

**Fig. 4.**
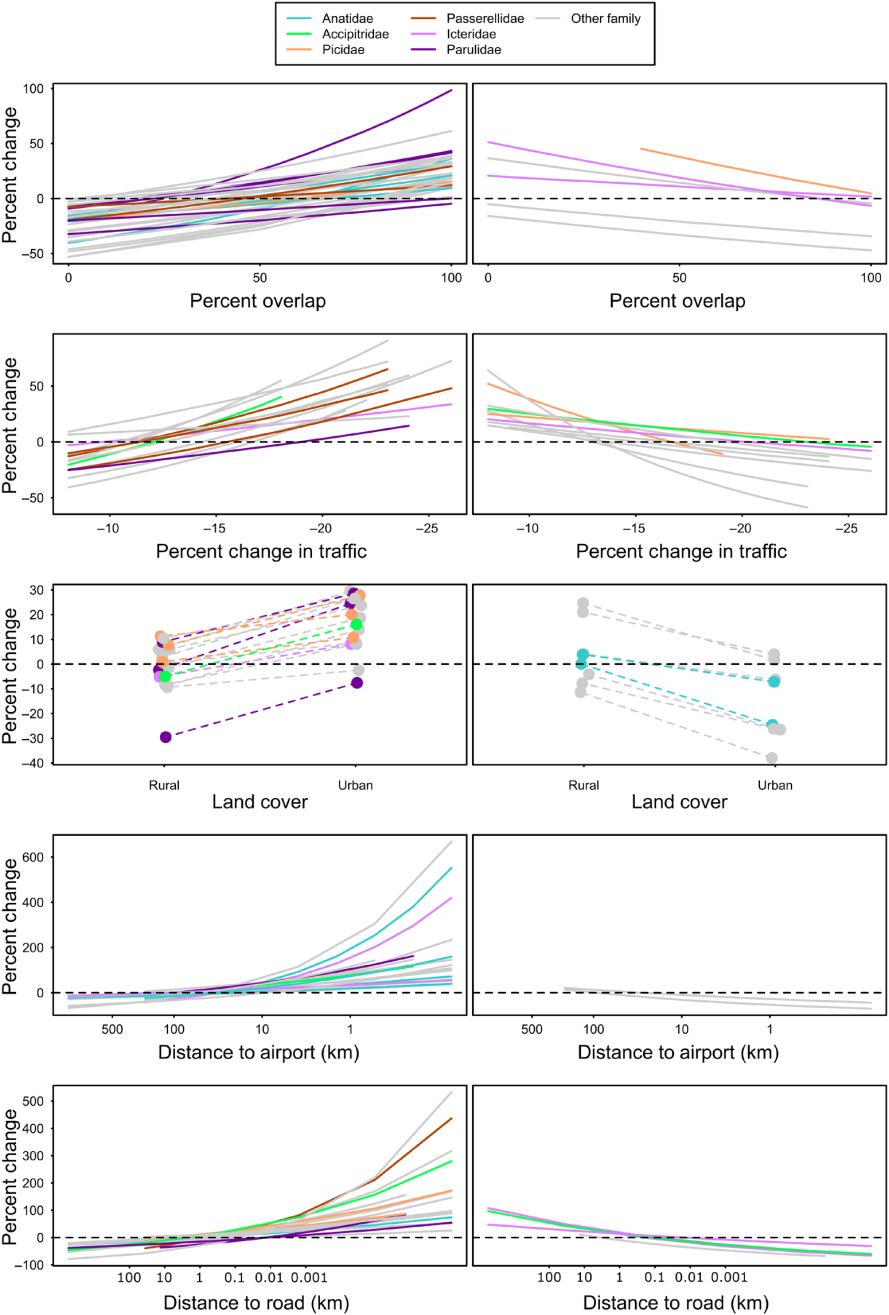
Effect of pandemic on bird counts. *Y* axis shows percent change in expected bird counts relative to predictor variables during the pandemic compared with prepandemic levels: the relative difference between the 2017–2019 and 2020 curves in Fig. 3 and fig. S11. Taxonomic families for which the analysis included >3 representative species are displayed in color. Only nonzero (probability >0.95) interactions are shown. Increases (left) and decreases (right) in relation to pandemic-altered areas are shown separately for easier visualization.

We detected changes in bird counts across a wide diversity of taxonomic families and functional guilds, from hawks to hummingbirds (Figs. 2 to 4). Variability in responses within and among taxonomic groups was high such that most families included some species that consistently increased, consistently decreased, or had mixed responses. However, there were some taxonomic trends. All demonstrable effects for members of Parulidae (New World warblers) and Passerellidae (New World sparrows) represented increases in bird counts in response to declining human activity (Fig. 2), suggesting that high levels of human activity suppressed the abundances of these families before the pandemic. This is particularly notable, as these two families account for nearly 50% of the 3 billion birds lost in North America since 1970 (*17*). Conversely, two nonnative species that are closely associated with urban centers in North America, the rock pigeon (*Columba livia*) and house sparrow (*Passer domesticus*), did not experience any demonstrable effects of lockdowns. The Icterid species that we studied, all of which are well-adapted to human-altered landscapes, were relatively more likely to decline in counties with more migration overlap or as distance to roads decreased (Figs. 2 and 4), showing the opposite trend compared with many other species. Full posterior summaries for all species and interaction coefficients are in figs. S10 and S11.

Two-thirds of our focal species showed changes in counts relative to the among-county indices of human activity. The degree of migratory overlap affected more species than any of the other indices, and the counts of these species were 5.8 times more likely to increase than decrease in counties with higher migration overlap (Table 1; e.g., Fig. 3A). This suggests that, during the pandemic, many birds used urban areas much more during their migration than they otherwise would have. Migratory stopover sites are critical to survival during migration (*18*), meaning that any improvement in the quality of such places could have large conservation implications. Counts of many species changed in relation to the change in traffic among counties, and 1.4 times more species displayed increased (e.g., bald eagle, *Haliaeetus leucocephalus*; Fig. 3B) than decreased counts in counties with greater decreases in traffic. We note that many cities outside of the United States and Canada experienced greater travel restrictions than those examined here (the range in traffic reduction among counties in this study was 8 to 25%), and thus, the degree of lockdown might have had an even greater impact on other continents.

More than half of our focal species showed changes in response to human activity within counties during the pandemic (Table 1 and Fig. 2). Counts of focal species were more than twice as likely to increase rather than decrease in urban compared with rural habitats during the pandemic (e.g., Fig. 3C). Species were markedly more likely to have increased than decreased counts close to airports (9 times) and major roads (3.4 times), compared with the prepandemic period (Table 1 and Fig. 3, D and E). Detections of ruby-throated hummingbirds (*Archilochus colubris*), for example, greatly increased close to airports during the study period (Fig. 3D). The specific mechanisms that explain local-scale effects of lockdowns remain unknown, but plausible hypotheses include decreased noise (*10*, *19*–*20*), altered species interactions (*21*), or decreased air pollution (*4*, *22*) with declining human activity. Many species were likely influenced by multiple factors; we encourage future research that can elucidate the mechanisms that explain these changes. Our results included a few examples of species exhibiting decreased abundance near roads during the pandemic. Human disturbances such as vehicular traffic or industrial noise can have some benefits for wildlife, such as increasing availability of roadkill for scavenging (*23*) or displacement of predators or competitors of focal species (*11*). However, the reduction in heavy vehicle traffic, especially associated with airports, apparently benefited many bird species by providing more functional habitat.

Not only were many species affected but also many of the effect sizes that we observed for the interactions with indices of human activity were large (Fig. 4), suggesting strong impacts of reduced human activity on birds. For example, counts of some species were several hundred percent higher than expected near airports (Fig. 4). Carolina wren (*Thryothorus ludovicianus*) abundances more than doubled near roads (fig. S11), and strong negative relationships with roads for barn swallows (*Hirundo rustica*), gray catbirds (*Dumetella carolinensis*), palm warblers (*Setophaga palmarum*), and other species almost completely disappeared during the pandemic (Fig. 3E and fig. S11). This emphasizes the importance of the lockdowns to many species and that even moderate reductions in human activity could have meaningful conservation implications across some species’ ranges.

Our broad, multispecies approach highlights not only how widespread the effects of the pandemic may have been but also how its impacts varied in scale among species. The mechanisms at play in the responses of different species could be quite different, and more species-specific analyses will ultimately be needed to fully explore those effects. Behavioral changes resulting from lockdowns have been documented in songs of white-crowned sparrows [*Zonotrichia leucophrys* (*10*)], in habitat use by waterbirds in a 7-ha wetland in Italy (*5*), in activity budgets of rock pigeons around Singapore (*24*), and in the use of Australian beaches by Torresian crows [*Corvus orru* (*25*)]. These diverse impacts demonstrate that responses of birds to the pandemic were not limited to North America. Other studies have documented changes to bird songs in response to reduced vehicle noise (*10*) and a shift in avian predator behavior leading to reduced reproductive success in a colonial seabird (*26*), emphasizing how diverse the species-specific effects of lockdowns were.

Note that our multispecies approach precluded the inclusion of species-specific predictors, and thus, the expected mean count for any particular species contains considerable unexplained residual variation. Semistructured programs like eBird are designed to use large sample sizes to evaluate changes in populations and communities despite such variation. Our goal was to determine whether the pandemic altered average bird abundance for a diverse group of birds across the widest geographic extent possible, and we recommend that any model attempting to predict bird abundance at a particular time and place include additional factors to improve predictive power for specific species.

Our analytical approach greatly reduced the possibility that changes in birder behavior during the pandemic could have affected our results. Our spatially explicit subsampling of checklists within counties resulted in equal sample sizes and nearly equal distributions of our predictor variables before and during the pandemic (figs. S1 to S4 and table S2). In this way, we minimized the effects of varied observer effort, and of birders surveying different locations within counties before and during the pandemic. Our focus on assessing changes in the relationships between bird counts and indices of human activity, rather than comparing abundance per se between pandemic and prepandemic periods, also helped insulate our results from variation in the observation process. Detectability of birds can be lower in noisy environments, as noise can mask bird sounds and distract observers from detecting birds (*27*). However, using independent measures of detection probability (table S3) available for most of our focal species, we found no evidence that smaller [*P* > 0.219; see (*28*)] or less detectable (*P* > 0.138) species were more likely to appear to respond to declining human activity during the pandemic (tables S4 and S5). The only significant relationship between the tendency to alter habitat use during the pandemic and mean size or detectability of our focal species was that larger species were more likely to alter their habitat use near airports (*P* < 0.047); this is the opposite of the pattern that we would expect if the apparent effects of lockdowns on avian abundance were driven by observer bias. The taxonomic diversity of species showing changes in these relationships adds circumstantial evidence that apparent changes in bird counts likely do not result from changes in detectability of birds by birders. Furthermore, we note that many species exhibiting increased counts in response to decreased human activity (such as ducks, wading birds, and raptors) are species that are usually detected visually and are thus relatively insensitive to variation in detectability caused by noise. Together, these results suggest that it is unlikely that pandemic-related changes in detection were a major cause of the patterns that we observed. Nonetheless, we cannot rule out the possibility that some species were more detectable during than before the pandemic.

Our results suggest that travel restrictions increased use of human-altered landscapes by many bird species in response to decreases in human activity. However, we cannot yet be certain that these trends resulted in conservation gains, especially given the quick recovery of traffic levels in many areas. For example, birds that occurred in relatively undisturbed territories during peak lockdown may have been exposed to increasing traffic noise and road mortality levels once lockdowns were eased, resulting in ecological traps during the ensuing breeding season (*29*–*32*). Further research should assess whether demographic parameters, such as mortality, nest success, and fecundity, also changed during the pandemic. Nonetheless, our results demonstrated that a reduction in human activity affects the distribution of many North American bird species. The widespread increases in counts of birds in response to reduced human activity during the pandemic suggest that a sustained reduction of vehicular traffic and human activity might have lasting benefits to birds.

The reduction of traffic associated with the sudden closure of global economies had tremendous negative impacts on livelihoods of many people, and these impacts must be addressed to achieve goals of social, economic, and environmental sustainability. However, as we move toward a post–COVID-19 society, our results suggest that finding ways of replicating some of the decreases in human activity that occurred during the pandemic could benefit many species of birds and other wildlife. Simple solutions to reduce noise, such as sound barrier walls, have been shown to mitigate the effects of industrial noise on birds (*11*). New technologies and policies may also help reduce road traffic, for example, by permanently increasing the work-from-home habits developed during pandemic lockdowns. Out of this event of great human suffering, we hope that, as a society, we gain knowledge and humility that will “challenge humanity to reconsider our future on Earth” (*3*) and that we will choose to make real-world changes that benefit both humans and other species.

## MATERIALS AND METHODS

### Study area

Our study included every county (“census division” for Canada) in the United States and Canada that contained a municipality with more than 50,000 residents, an international airport within municipal boundaries (*n* = 95), and at least 200 eBird checklists during our study period (*n* = 93; data S1). We chose 50,000 residents because it is a threshold used to describe metropolitan areas by both Statistics Canada and the U.S. Census Bureau, and it limited our focal cities to those that had sufficient numbers of eBird checklists for analysis. We used only cities that included international airports within municipal boundaries to evaluate impacts of distance to airport on changes in bird populations. For cities spread over multiple adjacent counties, we included each county that encompassed that city if the county included at least 10% of the city’s population. In Canada, counties were defined by the second-level divisions in the Database of Global Administrative Areas (*33*), which generally correspond to census divisions.

### Data collation

We used bird data recorded by community science volunteers participating in the online eBird program. Volunteers of various skill levels entered observations of bird presence and abundance using semistructured surveys known as checklists, each of which is a record of all birds the observers (or groups of observers) found during a given period at a given location. Each checklist is stored in conjunction with relevant effort metadata (e.g., duration and distance traveled) in the eBird database (*13*, *14*). We used the eBird Basic Dataset, version May-2020, from which we extracted data from our focal counties for March to May 2017–2019 (prepandemic period) and March to May 2020 (pandemic period). As eBird data were collected opportunistically by volunteers, we filtered the data so that we could be confident that observed changes in relative abundance did not result from changes in methods or survey effort in space or time (*15*, *34*). To control for effort-related biases in detection, checklists were only included if (i) users indicated that they recorded all birds detected during the survey, i.e.,

“complete” checklists from which we could obtain both detected presence and pseudo-absence; (ii) the protocol type was either “stationary” or “traveling”; (iii) duration was ≤5 hours; (iv) distance traveled was ≤3 km; and (v) the number of observers was ≤10 (*34*). Distance traveled and duration of each checklist were also included in our statistical models as covariates. In addition, because new eBird users have consistently lower detection rates than experienced users (*35*), and because of anecdotal suggestions that many people at home during the pandemic may have started using eBird in 2020, we excluded any checklists from 2020 observers who did not also submit data from the same county at least once in the same months during the prior 3 years.

Because of computational constraints and to ensure that sample sizes were similar among counties, we subsampled checklists using the procedure described below to a maximum of 1000 per county for use in modeling. The spatial distribution of eBird surveys is biased toward areas of high population density, so our decision to focus entirely on counties including cities with more than 50,000 residents helped to reduce variability in eBird sampling density among counties. To further reduce bias that might result from checklists overrepresenting specific locations within counties, we used a stepwise, stratified process to thin the data within counties. We split each county into a hexagonal grid with midpoints separated by 3 km, and randomly sampled a pair of checklists, each with one list from 2017 to 2019 and one from 2020, from each grid cell sequentially, until each cell no longer had a suitable pair or the maximum number had been reached. Choosing the same number of prepandemic and pandemic checklists from each cell minimized the effects of birders choosing to visit different areas during 2020 than previously. Drawing from grid cells sequentially ensured that our subsample had the largest possible geographic coverage within each county. Following this procedure, two counties containing fewer than 200 checklists were dropped from the analysis, leaving a total of 93 counties, 80 of which contained the maximum of 1000 checklists and 13 with fewer (ranging from 204 to 954). To ensure that sample sizes were sufficient to evaluate both within- and among-county responses to declining human activity, each species was modeled with data from all counties in which it was present in at least 5% of checklists. Only species for which that threshold was met in at least 30 counties were included in the analysis. The count of each focal species, including zeros if the species was not detected, was extracted from each checklist.

The natural log of the distances of each eBird checklist to the nearest major road [defined as level 1 and 2 roads (*36*), which generally correspond to national, state, and county highways] and the nearest international airport (*37*, *38*) were calculated using the Near tool within ArcGIS (*39*). These variables were centered so that zero indicated moderate distances to roads or airports. Note that distance was measured to the single point location chosen for each checklist, which often includes some error, and any single checklist could have involved travel up to several kilometers from the starting point. Therefore, any relationship between distance to a major road or airport and the expected mean count of a bird species should be interpreted as a general relationship over long-run averages.

We assessed whether counts of each species changed in urban and rural sites using land cover data derived from the North American Land Change Monitoring System 30-m resolution land cover layer, which is based on 2015 Landsat satellite imagery for Canada and the United States (*40*). We reclassified all land types not in the “urban” category into a single “rural” (nonurban) category and then calculated a binary land cover score for each eBird location based on whether the rounded proportion of pixels within 50 m of the location of each checklist was primarily urban or rural. We used the binary measure rather than a continuous measure of percent urban for simplicity, and because the majority of checklists (64%) were 100% urban or 100% rural. We then scored each checklist as 0 = mostly urban or 1 = mostly rural. We note that the term rural also includes extensive undeveloped areas within municipal city limits, such as large parks or bodies of water.

To generate an index of relative change in vehicular traffic among counties (change in traffic), we used COVID-19 Community Mobility data (*41*), which are based on movement of mobile phone users who shared their locations using the Location History setting on their phones. These data represent changes in mobility relative to expected values calculated daily, relative to the appropriate day of the week averaged across the 5-week baseline period from 3 January 2020 to 6 February 2020. Mobility data were assessed at the scale of counties within the United States, and at the scale of provinces in Canada (*41*), where data at the county level were not present. We created an index of change in traffic during the pandemic period using time spent at home as a negative index of change in traffic volume (fig. S1) such that an increased period of time at home indicated decreased traffic volume. This index was chosen on the basis of (i) our expectation that time spent at home is a negative index of time spent in all activities, cumulatively, outside the home; (ii) the high negative correlation between time spent at home and time spent at either work (*r* = −0.901, *n* = 9021) or retail and recreation facilities (*r* = −0.666, *n* = 9021); and (iii) the absence of other data sources on traffic volumes at the scale of individual counties. We averaged the relative daily change in time spent at home from 1 March 2020 to 31 May 2020 for each county as our measure of change in traffic during the pandemic period. This variable was centered for modeling.

To account for the fact that many migrant species traveled through different counties at different times, we created a species-specific index of overlap between the peak period of human travel restriction and peak bird migration period for each county (hereafter, “overlap”). We defined the period of peak travel restriction as the days between the 25th and 75th percentiles of the cumulative sum of changes in traffic, and the peak migration period as the days between the 25th and 75th percentiles of the cumulative sum of number of birds recorded among sample checklists (Fig. 1). These “peak” periods reflect periods when both traffic levels and migration were at their highest levels. We calculated the proportion of the migration peak that overlapped with the travel restriction peak and then subtracted one from that proportion to create a measure that ranged from negative one (lowest possible overlap) to zero (highest possible overlap). This approach ensured that the main effect of any variable interacting with overlap indicated the effect of that variable when migration coincided most strongly with decreased traffic (i.e., overlap = 0) to facilitate interpretation of parameter estimates. This approach was used for all species, even those that were primarily residents in some counties, for which overlap was generally at or close to 100%. In these cases, one can think of the overlap term as representing the co-occurrence of peak lockdown with peak presence.

We investigated collinearity among predictors and found few correlations among variables. Correlation between logged distance to road and logged distance to airport from all checklists was statistically significant, due to the very large sample size, but ρ was very close to zero (0.069; fig. S5), suggesting that less than 1% of the variation in each variable was explained by the other. The change in traffic at the level of the county was unrelated to the distance to road or airport (figs. S6 and S7). As might be expected, rural checklists were located slightly further from airports and major roads than urban checklists (figs. S8 and S9); however, the large overlap in their distributions makes any concerns about collinearity among those variables relatively minor.

### Statistical models

Relative abundance of each species (species-specific count from each checklist) was modeled as a negative binomial process within a generalized linear mixed model, as the negative binomial distribution was found to provide the best fit in prior analyses of eBird data (*34*). County was included as a hierarchical random effect. Fixed variables included the main effect of the pandemic period (i.e., prepandemic or during pandemic), distance traveled during each checklist (kilometers), duration of each checklist (minutes), main effects of the five variables of interest [(i) change in traffic, (ii) migration overlap, (iii) distance to major road, (iv) distance to airport, and (v) urban versus rural location], and interactions between the five variables of interest and the pandemic period. We used March to May of 2017–2019 to describe prepandemic baseline bird counts (code = −1) and considered March to May of 2020 to be the pandemic period (code = 0).

We performed a Bayesian regression for each species using weakly informative priors for all parameters. All covariates were given priors drawn from a normal distribution with mean of zero, given that we had no preconceived notion about the effect size of any of the parameters, and SD of 2.5 divided by the SD of the covariate. This prior is informative enough to create reasonable limits on values for the coefficients, thus promoting model fit, without severely constraining the posterior within those limits (*42*). The grouped (i.e., random) county-specific intercepts were assigned a multivariate normal distribution with mean of zero and SD of 2.5, applied as a decomposition of a covariance matrix with regularization, concentration, shape, and scale parameters all equal to one, as described in (*43*). The negative binomial reciprocal dispersion parameter had an exponential prior with λ = 1. All of these choices for priors provided relatively flat probabilities over realistic possibilities for the coefficients, without being too broad as to negatively influence the stability of the computations. Prior-posterior overlap was inspected visually for each species model to ensure no posteriors heavily mirrored prior distributions.

We implemented the models in the Stan programming language using the R (*44*) package rstanarm v2.21.1 (*45*). Each model was initially run with five chains of 1000 iterations, following a 300-iteration adaptation, with a thinning rate of one. Convergence was assessed visually and by inspecting *Rˆ* values and effective sample size of posteriors (data S3). Seven species displayed a slight lack of chain mixing and were rerun with two to four times the thinning rate and number of iterations to provide appropriate samples from posterior distributions. Each of the five interactions of interest was assessed by calculating its marginal effect (Fig. 2 and fig. S11) using the R package ggeffects (*46*) and then interpreting the directionality of the shift in relationship from prepandemic to pandemic periods. This approach uses the 95% credible interval of the focal coefficient and average values for all other coefficients to predict the expected count in a single county. Therefore, the value of the expected count is less meaningful than the proportional change due to the pandemic (Fig. 3).

Our interpretation of results focused on interaction coefficients with a posterior probability ≥0.95 of being either greater or less than zero (fig. S10), and we considered all other interaction terms as not providing clear enough evidence of a change in the relationship due to the pandemic. We considered this a conservative cutoff and stress that a lack of demonstrable result for a particular species-by-variable combination here should not be taken as strong evidence that the pandemic did not affect that species in that way. Relationships that did show a high posterior probability of changing during the pandemic were designated as either showing increased or decreased counts relative to declining human activity during the pandemic based on the direction of the shift. We considered changes in the intercept relatively uninformative, as this simply indicated a different abundance in 2020, which could have varied regardless of the pandemic. Our interpretation relied on directional shifts in the slope. For example, ruby-throated hummingbirds were more abundant in 2020 close to airports but not far from them, showing an increase in their counts in response to declining human activity during the pandemic (Fig. 3D). Conversely, indigo bunting counts were greater in proximity to airports during prepandemic years, but that relationship mostly disappeared during 2020, showing a decrease in response to the decline in human activity (fig. S11). In cases where predictor variables were transformed for modeling purposes, they were back-transformed for visualization in figures such that the right side of the *x* axis always shows areas more altered by the pandemic.

### Detectability

We used the following approaches to evaluate whether the patterns that we detected in bird counts during the pandemic might have been caused by increased detectability of birds that would otherwise have remained undetected under normal anthropogenically modified acoustic conditions. First, we hypothesized that if apparent changes in bird counts were caused by increased detectability during the pandemic, then those species that are least detectable should show the greatest increases in counts in response to declining human activity during the pandemic. We evaluated this separately for each interaction term. To do this, we used an independently derived measure of detectability for each species by using a statistical method developed by Sólymos *et al*. (*47*). Briefly, removal sampling (*48*) was used to estimate the species-specific rate at which birds provide a cue that make them detectible (e.g., sing, call, move to a conspicuous perch, and conspecific interaction), and distance sampling (*49*) was used to estimate species-specific effective detection radius around a human observer. We estimated cue rates and effective detection radii using a point count database from the Canadian Wildlife Service and obtained previously published estimates of these parameters from (*50*). If estimates for a given species were available from both sources, we calculated the mean. Last, we used equations from (*47*) to estimate the total probability of detecting each species during a 5-min, 100-m-radius point count and considered this to be a relative measure of detectability among species. Point-count data used to estimate relative detectability were collected primarily from boreal forest, aspen parkland, and prairie grassland ecosystems in Canada. We were able to use these methods to calculate detectability for 54 of our 82 focal species. Detectability ranged from 0.06 (ruby-throated hummingbird, *A. colubris*) to 0.62 (American crow, *Corvus brachyrhynchos*), with a mean of 0.36 (SD = 0.12; table S3).

Our prediction was that if observed patterns were caused by detectability, less detectable species should show more increases in response to lockdown relative to more detectable species. To calculate the response variable, we therefore categorized species that exhibited increased counts in response to decreased human activity as 1 and species with decreased counts or no demonstrable responses as 0. We also wanted to test whether detectability might explain any observed changes in bird counts (both increases and decreases in response to decreased human activity), so we conducted a second suite of analyses in which the response variable was calculated by categorizing species with either increases or decreases as 1, and species for which there was no demonstrable change as 0. We then used a binomial process within a generalized linear mixed model to evaluate impacts of species-specific detectability on the probability of apparent increases (or apparent changes) in counts in response to declining human activity for each interaction term. We included taxonomic family as a random variable to account for potential similarities in relative detectability among related species (table S4).

Previous research has also demonstrated that smaller species are more likely to show decreased detectability near roads than larger species (*28*), so we also wanted to ensure that our analyses were not driven by body size. On the basis of (*28*), we hypothesized that if changes in bird counts were caused by smaller birds being more detectable during the pandemic, then smaller species should show the greatest increases in counts in response to declining human activity during the pandemic. We, therefore, used the same approach as described above for detectability analyses, but with average body size per species as the fixed independent variable. Body size data were obtained for each of our focal species from (*51*). As above, we assessed both whether smaller species were more likely to show increased counts in response to declining human activity and whether smaller species were more likely to show any differences in counts in response to declining human activity (table S5).
