# Supplementary material for: Reduced human activity during COVID-19 alters avian land use across North America
Source: Sci Adv. 2021 Sep 22;7(39):eabf5073. doi: 10.1126/sciadv.abf5073 (PMC10763905; doi:10.1126/sciadv.abf5073)
Supplement: Supplementary file 1 — Supplementary Text Figs. S1 to S11 Tables S1 to S5 Legends for data S1 to S4 [file sciadv.abf5073_sm.pdf]

Science Advances
AAAS

Supplementary Materials for

Reduced human activity during COVID-19 alters avian land use across North America

Michael B. Schrimpf, Paulson G. Des Brisay, Alison Johnston, Adam C. Smith, Jessica Sánchez-Jasso, Barry G. Robinson, Miyako H. Warrington, Nancy A. Mahony, Andrew G. Horn, Matthew Strimas-Mackey, Lenore Fahrig, Nicola Koper*

*Corresponding author. Email: nicola.koper@umanitoba.ca

Published 22 September 2021, *Sci. Adv.* **7**, eabf5073 (2021)
DOI: 10.1126/sciadv.abf5073

The PDF file includes:

Supplementary Text
Figs. S1 to S11
Tables S1 to S5
Legends for data S1 to S4

Other Supplementary Material for this manuscript includes the following:

Data S1 to S4

Supplementary Text

Model variables

Table S1 (p. 101) lists summary statistics for all the variables used in our models. Figures S1–S4 (pp. 3–6) and Table S2 (p. 102) describe the distribution of the distance traveled, duration, distance to road, distance to airport, and landcover (urban vs. rural) variables, and compares those distributions between the pre-pandemic and pandemic periods. The distribution of the change in traffic variable is displayed in the main text (Fig. 1A), and the overlap variable was specific to each species-by-county combination, and therefore not easily displayed (though, see Fig. 1B for a representative example).

Note that the model for each species only included a subset of counties, so the exact distribution of predictor data would have varied slightly for each.

Figures S5–S9 (pp. 7–11) compare the distributions of different predictors to assess collinearity.

Model results

Figures S10 (pp. 12–17) and S11 (pp. 18–100) are multi-page figures that present all of the 95% credible intervals and interaction plots (respectively) for all 82 species and all five interaction terms used in the study. Captions for Figs. S10 and S11 are placed on the page preceding each, on pp. 12 and 18, respectively.

Tables S3–S5 (pp. 103–105) relate to our post-hoc assessment of results in relation to independent measures of detection.

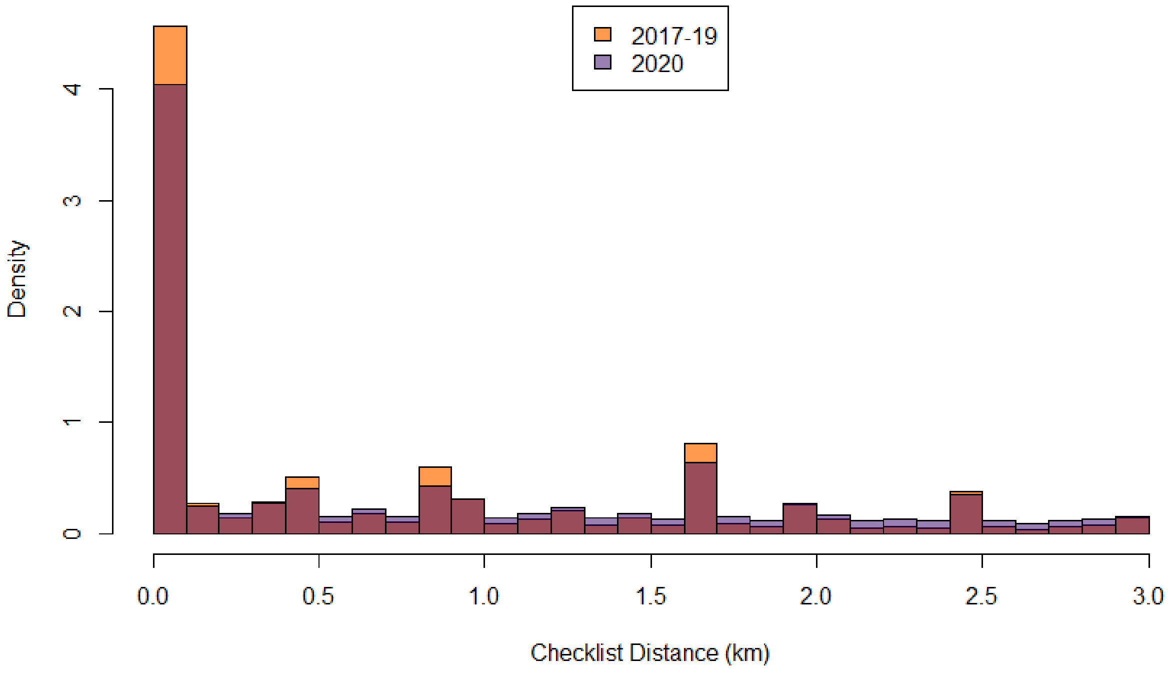

Fig. S1.

Distribution of distance traveled (in kilometers) recorded for all eBird checklists used in the analysis (from all counties), separated into the pre-pandemic and pandemic periods. The third color occurs where both histograms overlap.

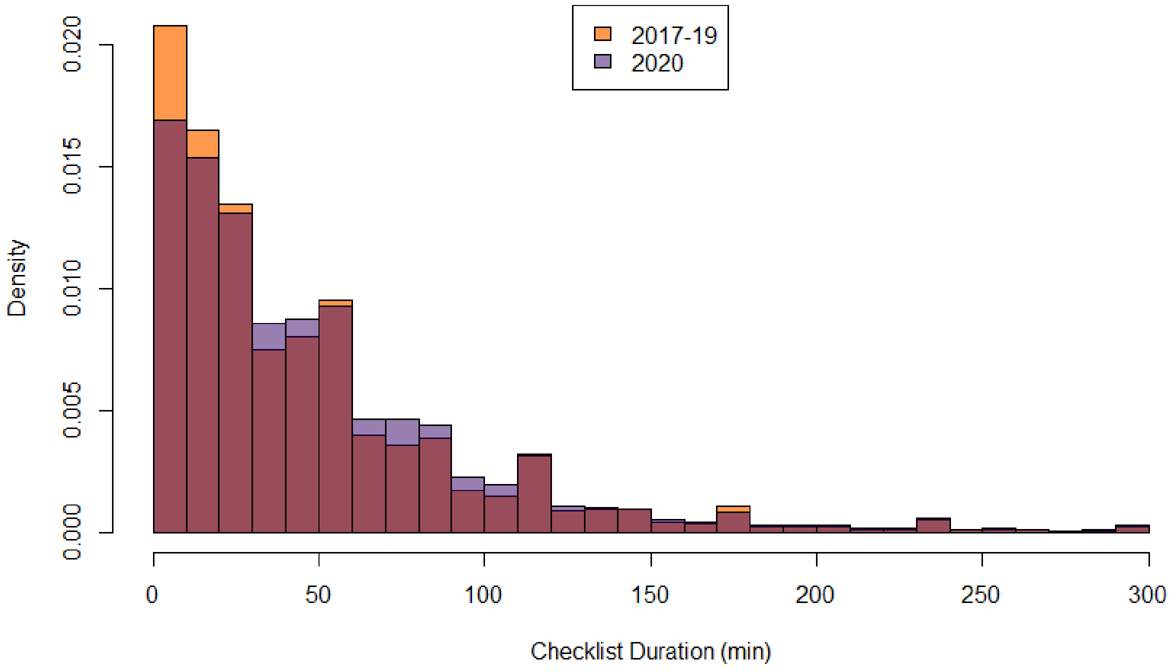

Fig. S2.

Distribution of duration (in minutes) recorded for all eBird checklists used in the analysis (from all counties), separated into the pre-pandemic and pandemic periods. The third color occurs where both histograms overlap.

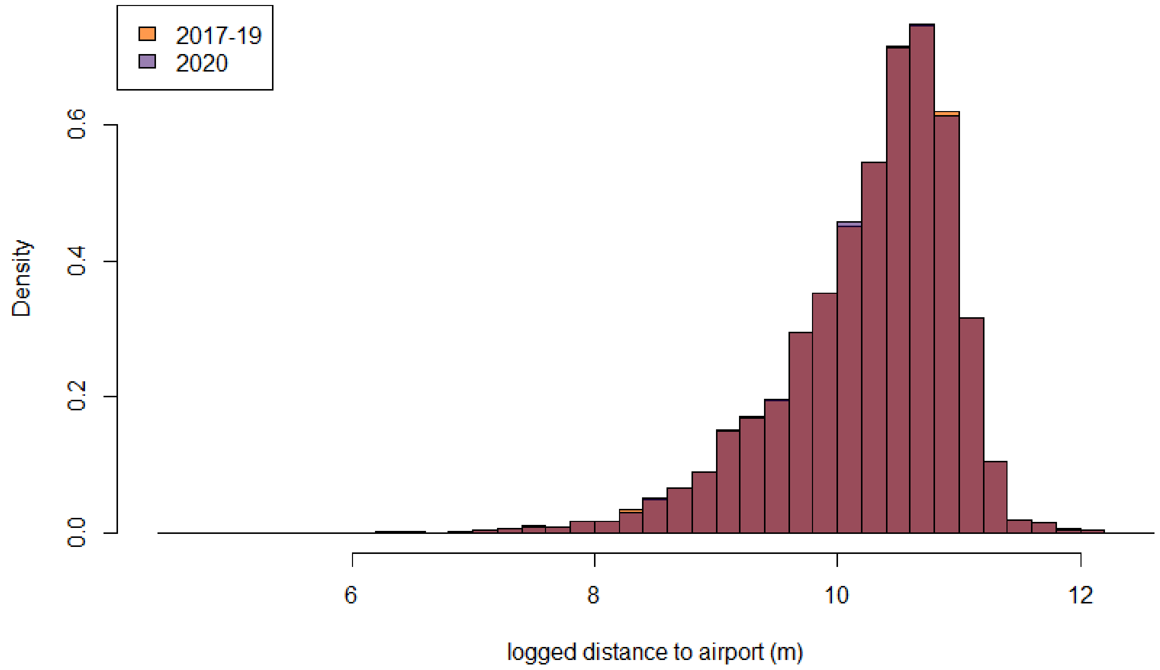

Fig. S3.
Distribution of logged distance to international airports recorded for all eBird checklists used in the analysis (from all counties), separated into the pre-pandemic and pandemic periods. The third color occurs where both histograms overlap.

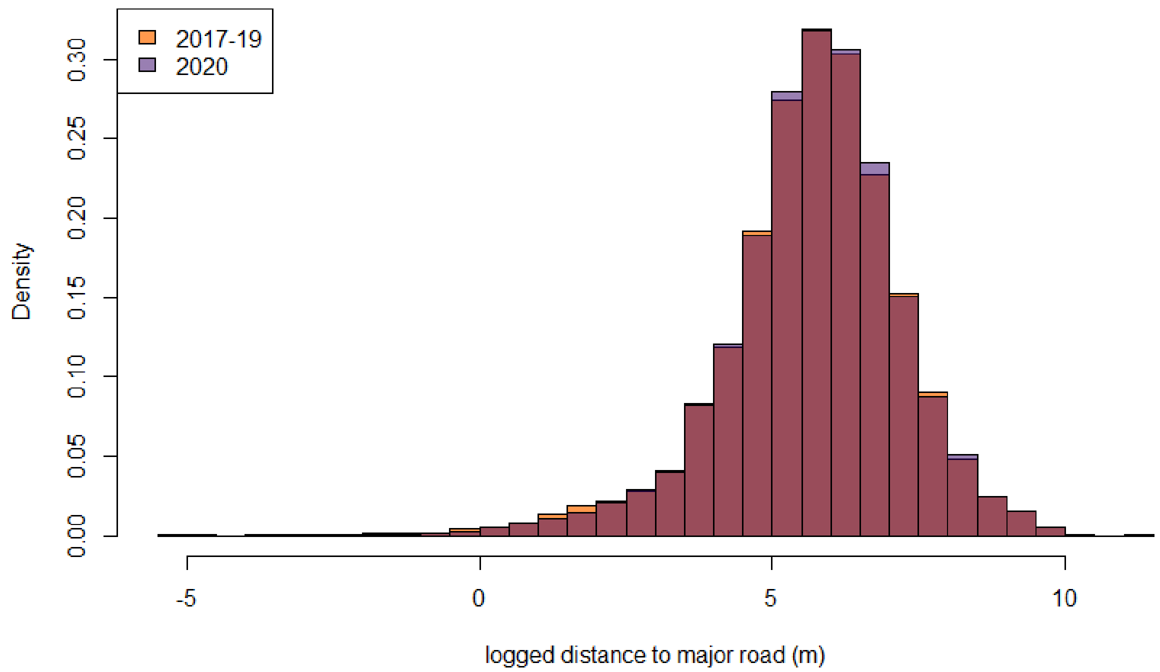

Fig. S4.

Distribution of logged distance to nearest major road recorded for all eBird checklists used in the analysis (from all counties), separated into the pre-pandemic and pandemic periods. The third color occurs where both histograms overlap.

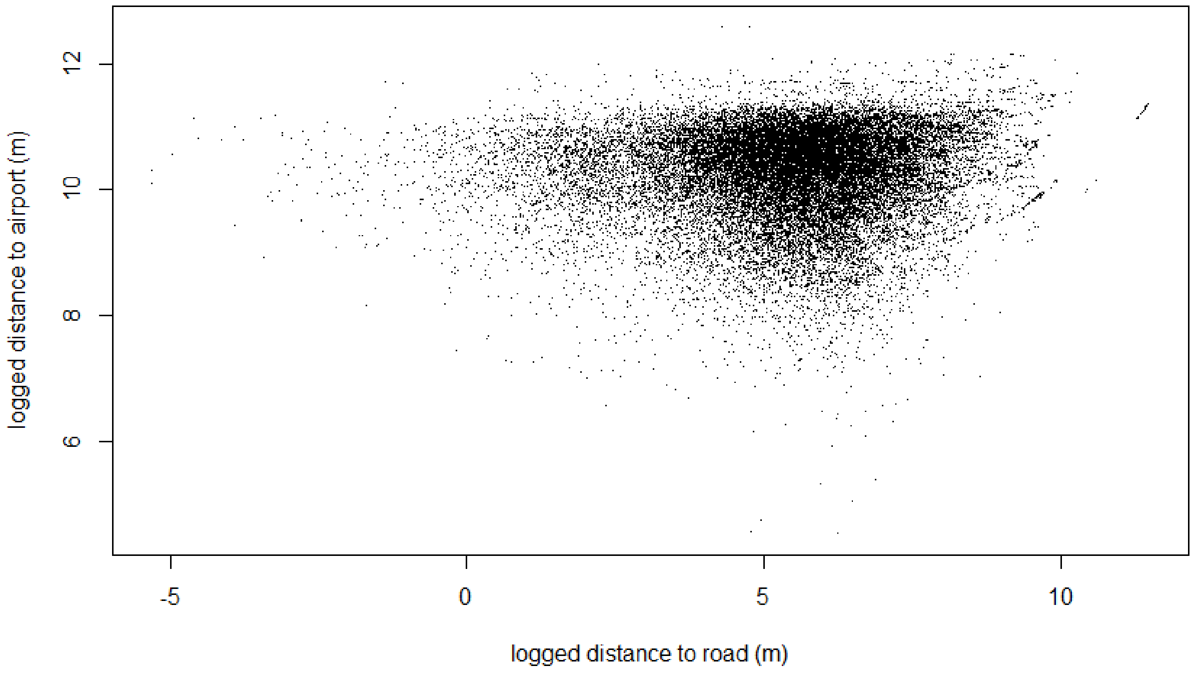

Fig. S5.

Plot of logged distance to road vs. logged distance to airport for all checklists. Pearson product moment correlation coefficient = 0.069 ($t = 20.4$, $df = 88604$, $p < 0.001$).

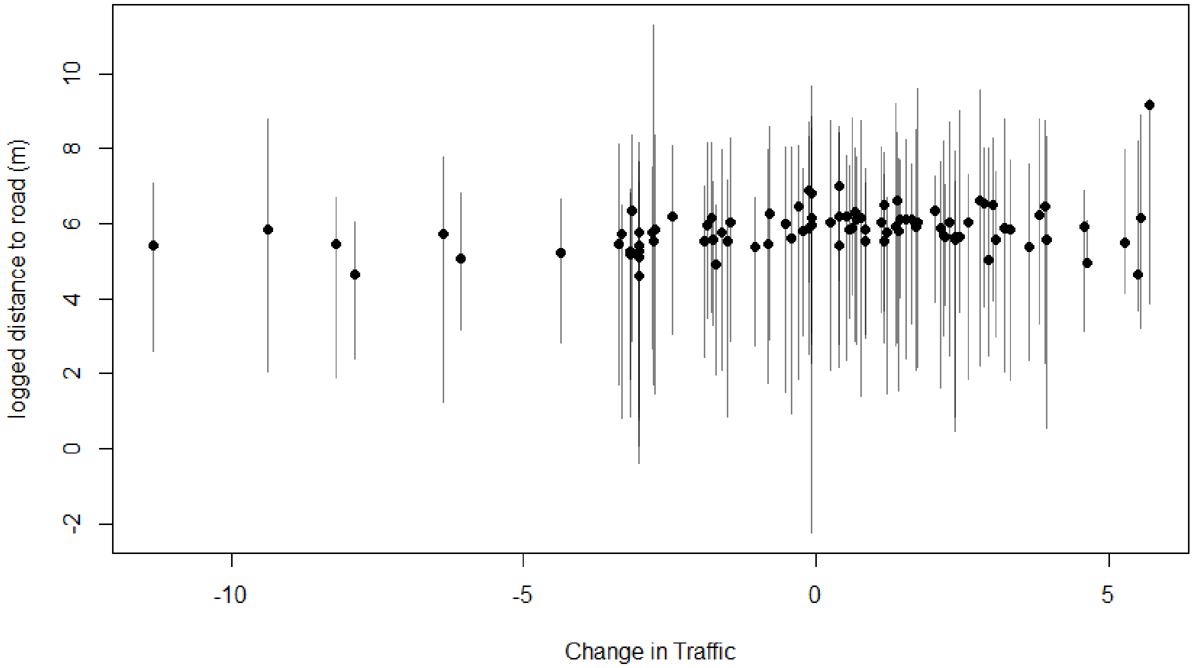

Fig. S6.

The relationship between the county-specific percent change in traffic during the pandemic (centered, such that 0 = the mean percent change) and the county-specific medians (points) and 2.5–97.5% quantiles (bars) of logged distance to major road.

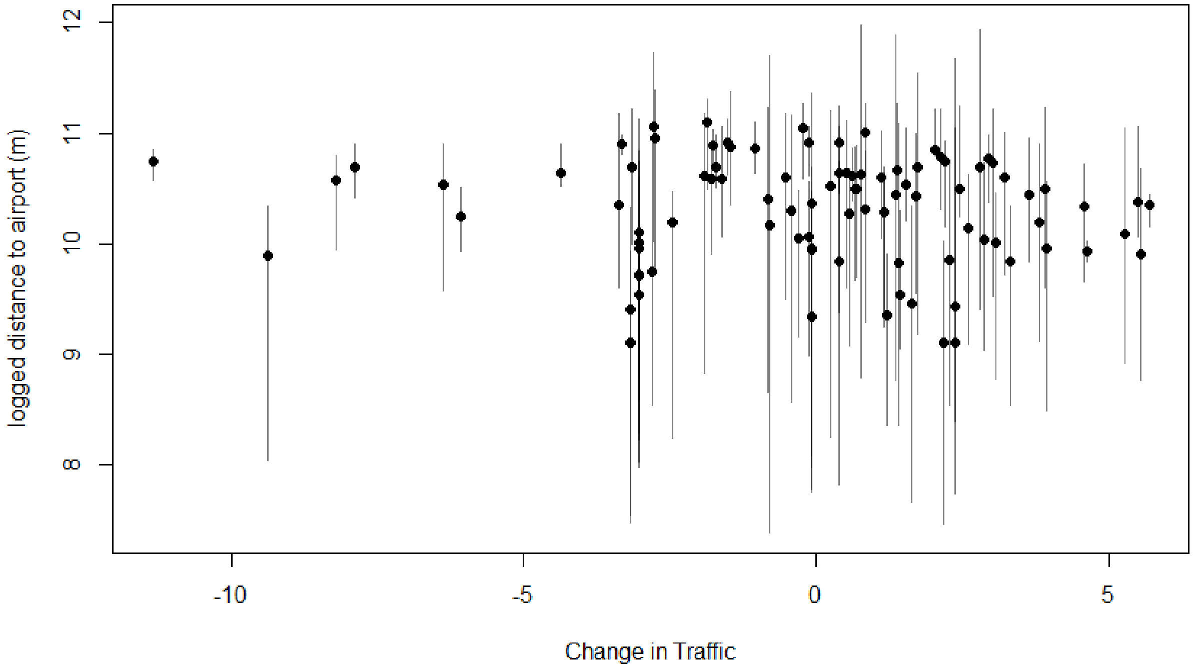

Fig. S7.

The relationship between the county-specific percent change in traffic during the pandemic (centered, such that 0 = the mean percent change) and the county-specific medians (points) and 2.5–97.5% quantiles (bars) of logged distance to airport.

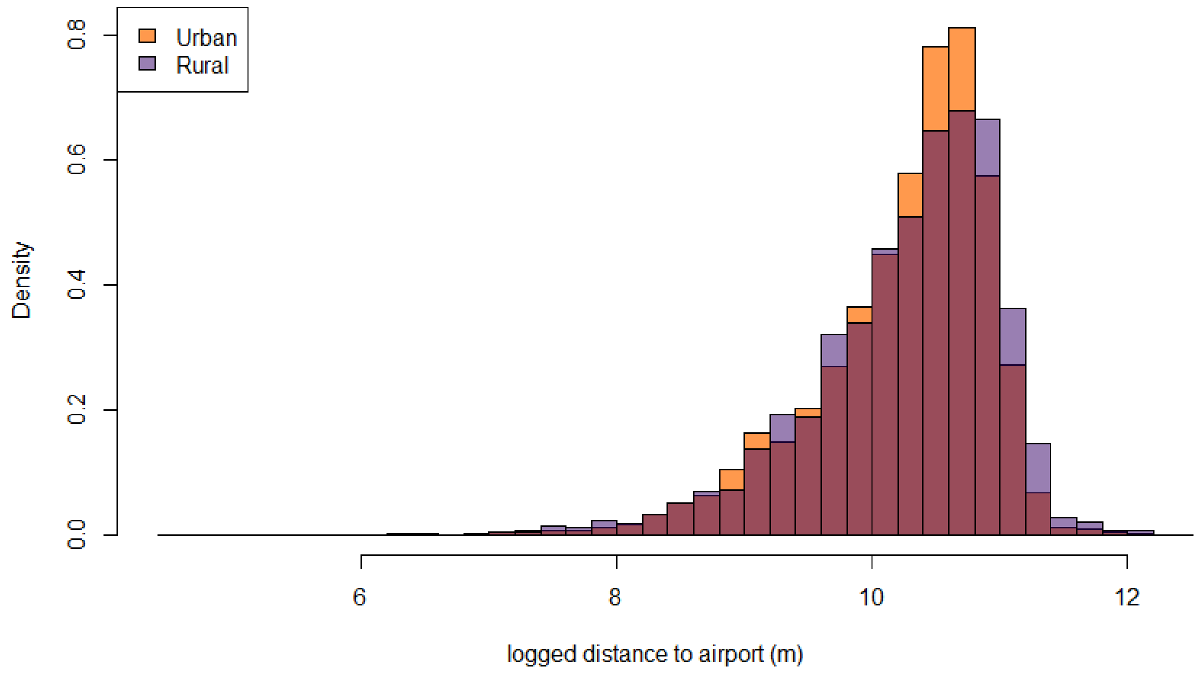

Fig. S8.

Distribution of logged distance to airport recorded for all eBird checklists used in the analysis (from all counties), separated into checklists coming from urban vs. rural habitat. The third color occurs where both histograms overlap.

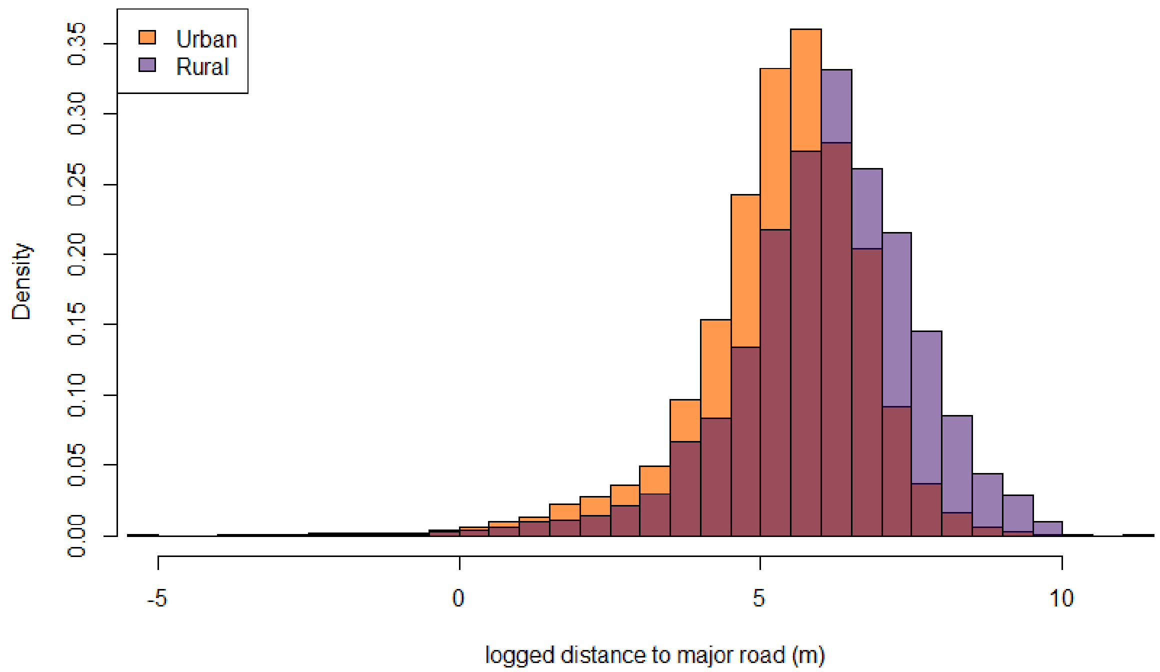

Fig. S9.

Distribution of logged distance to nearest major road recorded for all eBird checklists used in the analysis (from all counties), separated into checklists coming from urban vs. rural habitat. The third color occurs where both histograms overlap.

Fig. S10. (multi-page figure beginning on the next page)

Interaction coefficient posterior distributions. Medians and 95% credible intervals for all interaction coefficients for each species, labeled as: "overlap": overlap between peak migration and peak travel reductions; "delta_traf": percent change in human mobility during pandemic; "land" urban versus rural habitat; "air": distance to airport, and "road": distance to major road. Species with 95% credible intervals that do not overlap zero are in bold. For ease of comparison, the sign of all intervals has been transformed such that positive values indicate increases in counts in areas where human activity was reduced.

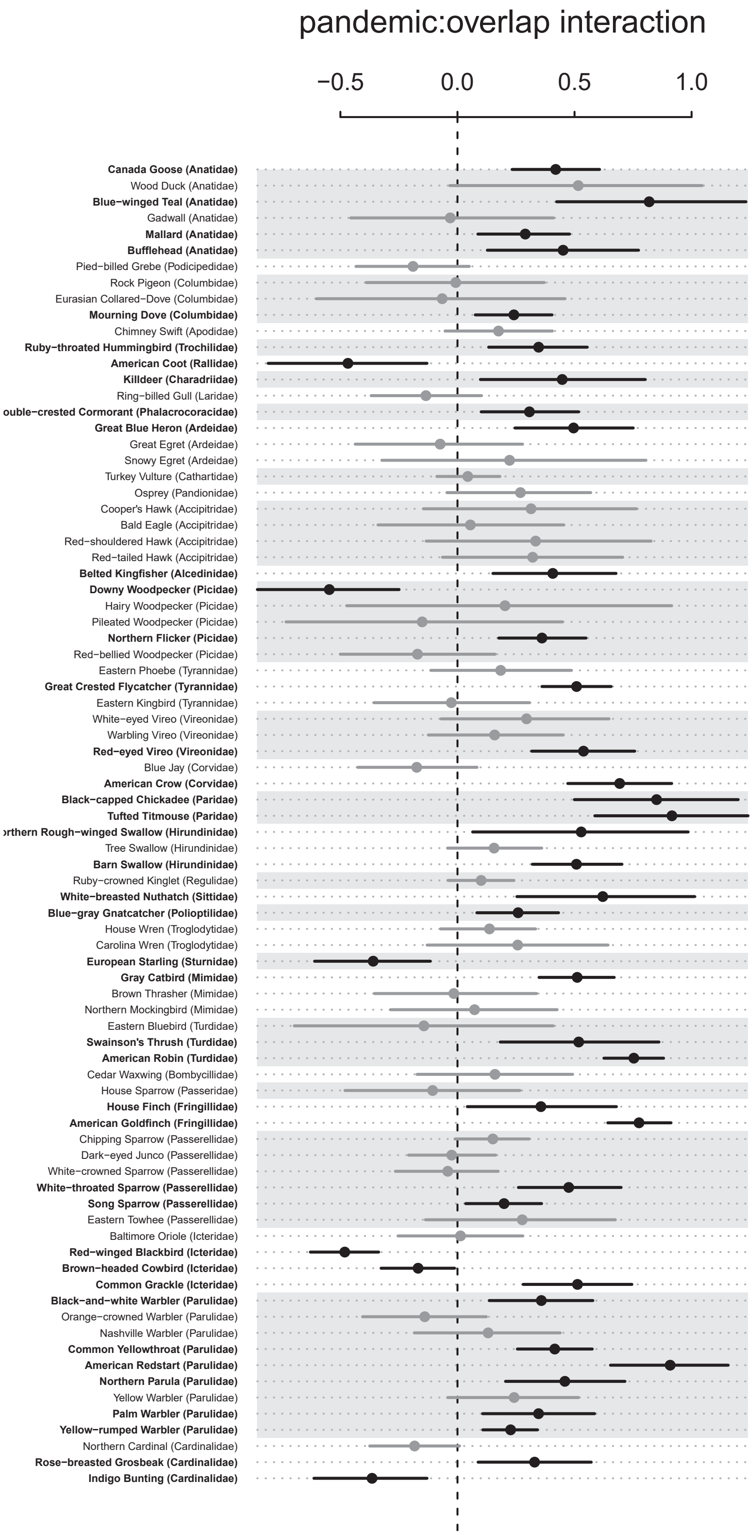

pandemic:overlap interaction
−0.5
0.0
0.5
1.0
Canada Goose (Anatidae)
Wood Duck (Anatidae)
Blue−winged Teal (Anatidae)
Gadwall (Anatidae)
Mallard (Anatidae)
Bufflehead (Anatidae)
Pied−billed Grebe (Podicipedidae)
Rock Pigeon (Columbidae)
Eurasian Collared−Dove (Columbidae)
Mourning Dove (Columbidae)
Chimney Swift (Apodidae)
Ruby−throated Hummingbird (Trochilidae)
American Coot (Rallidae)
Killdeer (Charadriidae)
Ring−billed Gull (Laridae)
ouble−crested Cormorant (Phalacrocoracidae)
Great Blue Heron (Ardeidae)
Great Egret (Ardeidae)
Snowy Egret (Ardeidae)
Turkey Vulture (Cathartidae)
Osprey (Pandionidae)
Cooper's Hawk (Accipitridae)
Bald Eagle (Accipitridae)
Red−shouldered Hawk (Accipitridae)
Red−tailed Hawk (Accipitridae)
Belted Kingfisher (Alcedinidae)
Downy Woodpecker (Picidae)
Hairy Woodpecker (Picidae)
Pileated Woodpecker (Picidae)
Northern Flicker (Picidae)
Red−bellied Woodpecker (Picidae)
Eastern Phoebe (Tyrannidae)
Great Crested Flycatcher (Tyrannidae)
Eastern Kingbird (Tyrannidae)
White−eyed Vireo (Vireonidae)
Warbling Vireo (Vireonidae)
Red−eyed Vireo (Vireonidae)
Blue Jay (Corvidae)
American Crow (Corvidae)
Black−capped Chickadee (Paridae)
Tufted Titmouse (Paridae)
orthern Rough−winged Swallow (Hirundinidae)
Tree Swallow (Hirundinidae)
Barn Swallow (Hirundinidae)
Ruby−crowned Kinglet (Regulidae)
White−breasted Nuthatch (Sittidae)
Blue−gray Gnatcatcher (Polioptilidae)
House Wren (Troglodytidae)
Carolina Wren (Troglodytidae)
European Starling (Sturnidae)
Gray Catbird (Mimidae)
Brown Thrasher (Mimidae)
Northern Mockingbird (Mimidae)
Eastern Bluebird (Turdidae)
Swainson's Thrush (Turdidae)
American Robin (Turdidae)
Cedar Waxwing (Bombycillidae)
House Sparrow (Passeridae)
House Finch (Fringillidae)
American Goldfinch (Fringillidae)
Chipping Sparrow (Passerellidae)
Dark−eyed Junco (Passerellidae)
White−crowned Sparrow (Passerellidae)
White−throated Sparrow (Passerellidae)
Song Sparrow (Passerellidae)
Eastern Towhee (Passerellidae)
Baltimore Oriole (Icteridae)
Red−winged Blackbird (Icteridae)
Brown−headed Cowbird (Icteridae)
Common Grackle (Icteridae)
Black−and−white Warbler (Parulidae)
Orange−crowned Warbler (Parulidae)
Nashville Warbler (Parulidae)
Common Yellowthroat (Parulidae)
American Redstart (Parulidae)
Northern Parula (Parulidae)
Yellow Warbler (Parulidae)
Palm Warbler (Parulidae)
Yellow−rumped Warbler (Parulidae)
Northern Cardinal (Cardinalidae)
Rose−breasted Grosbeak (Cardinalidae)
Indigo Bunting (Cardinalidae)

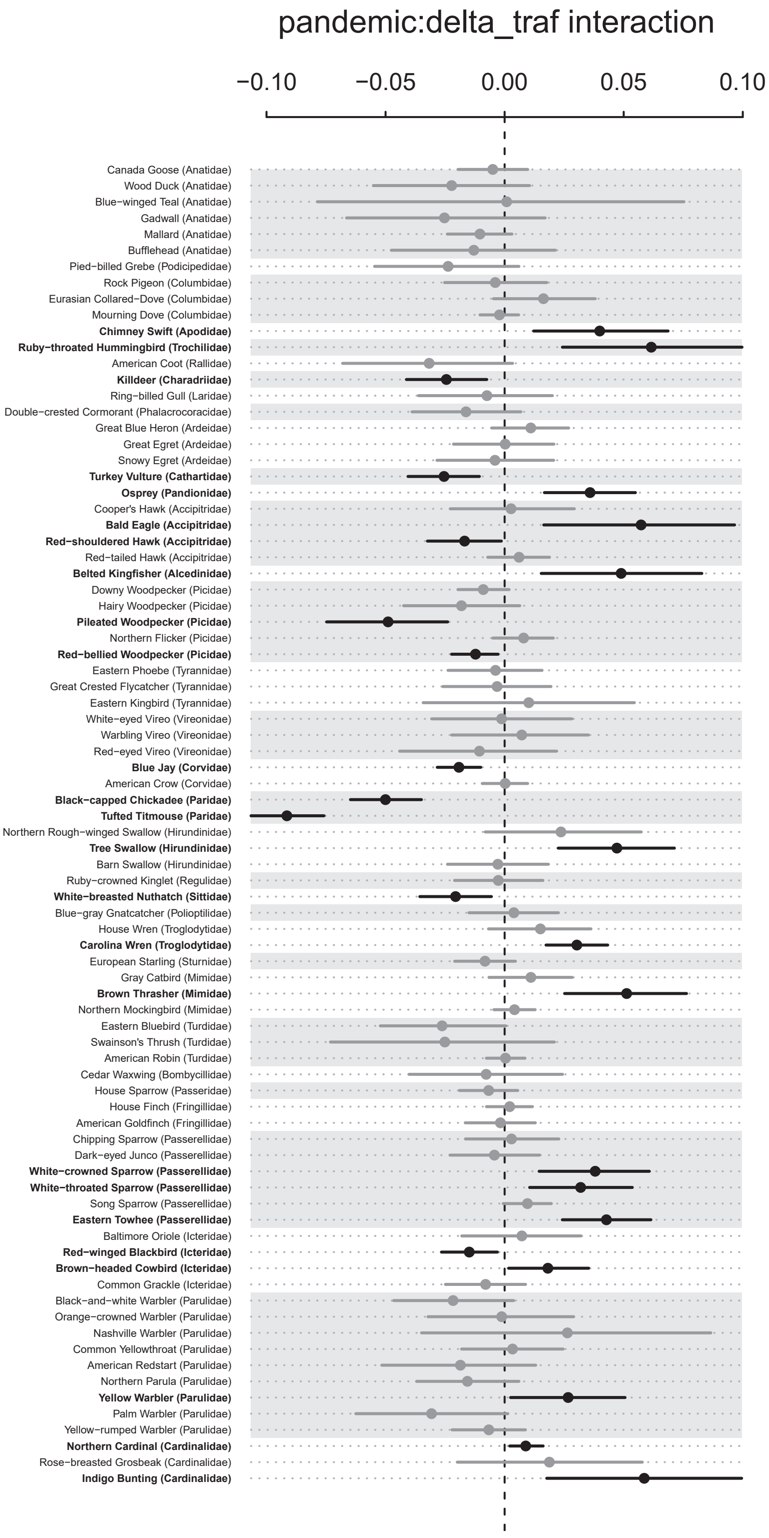

pandemic:delta_traf interaction
−0.10
−0.05
0.00
0.05
0.10
Canada Goose (Anatidae)
Wood Duck (Anatidae)
Blue−winged Teal (Anatidae)
Gadwall (Anatidae)
Mallard (Anatidae)
Bufflehead (Anatidae)
Pied−billed Grebe (Podicipedidae)
Rock Pigeon (Columbidae)
Eurasian Collared−Dove (Columbidae)
Mourning Dove (Columbidae)
Chimney Swift (Apodidae)
Ruby−throated Hummingbird (Trochilidae)
American Coot (Rallidae)
Killdeer (Charadriidae)
Ring−billed Gull (Laridae)
Double−crested Cormorant (Phalacrocoracidae)
Great Blue Heron (Ardeidae)
Great Egret (Ardeidae)
Snowy Egret (Ardeidae)
Turkey Vulture (Cathartidae)
Osprey (Pandionidae)
Cooper's Hawk (Accipitridae)
Bald Eagle (Accipitridae)
Red−shouldered Hawk (Accipitridae)
Red−tailed Hawk (Accipitridae)
Belted Kingfisher (Alcedinidae)
Downy Woodpecker (Picidae)
Hairy Woodpecker (Picidae)
Pileated Woodpecker (Picidae)
Northern Flicker (Picidae)
Red−bellied Woodpecker (Picidae)
Eastern Phoebe (Tyrannidae)
Great Crested Flycatcher (Tyrannidae)
Eastern Kingbird (Tyrannidae)
White−eyed Vireo (Vireonidae)
Warbling Vireo (Vireonidae)
Red−eyed Vireo (Vireonidae)
Blue Jay (Corvidae)
American Crow (Corvidae)
Black−capped Chickadee (Paridae)
Tufted Titmouse (Paridae)
Northern Rough−winged Swallow (Hirundinidae)
Tree Swallow (Hirundinidae)
Barn Swallow (Hirundinidae)
Ruby−crowned Kinglet (Regulidae)
White−breasted Nuthatch (Sittidae)
Blue−gray Gnatcatcher (Polioptilidae)
House Wren (Troglodytidae)
Carolina Wren (Troglodytidae)
European Starling (Sturnidae)
Gray Catbird (Mimidae)
Brown Thrasher (Mimidae)
Northern Mockingbird (Mimidae)
Eastern Bluebird (Turdidae)
Swainson's Thrush (Turdidae)
American Robin (Turdidae)
Cedar Waxwing (Bombycillidae)
House Sparrow (Passeridae)
House Finch (Fringillidae)
American Goldfinch (Fringillidae)
Chipping Sparrow (Passerellidae)
Dark−eyed Junco (Passerellidae)
White−crowned Sparrow (Passerellidae)
White−throated Sparrow (Passerellidae)
Song Sparrow (Passerellidae)
Eastern Towhee (Passerellidae)
Baltimore Oriole (Icteridae)
Red−winged Blackbird (Icteridae)
Brown−headed Cowbird (Icteridae)
Common Grackle (Icteridae)
Black−and−white Warbler (Parulidae)
Orange−crowned Warbler (Parulidae)
Nashville Warbler (Parulidae)
Common Yellowthroat (Parulidae)
American Redstart (Parulidae)
Northern Parula (Parulidae)
Yellow Warbler (Parulidae)
Palm Warbler (Parulidae)
Yellow−rumped Warbler (Parulidae)
Northern Cardinal (Cardinalidae)
Rose−breasted Grosbeak (Cardinalidae)
Indigo Bunting (Cardinalidae)

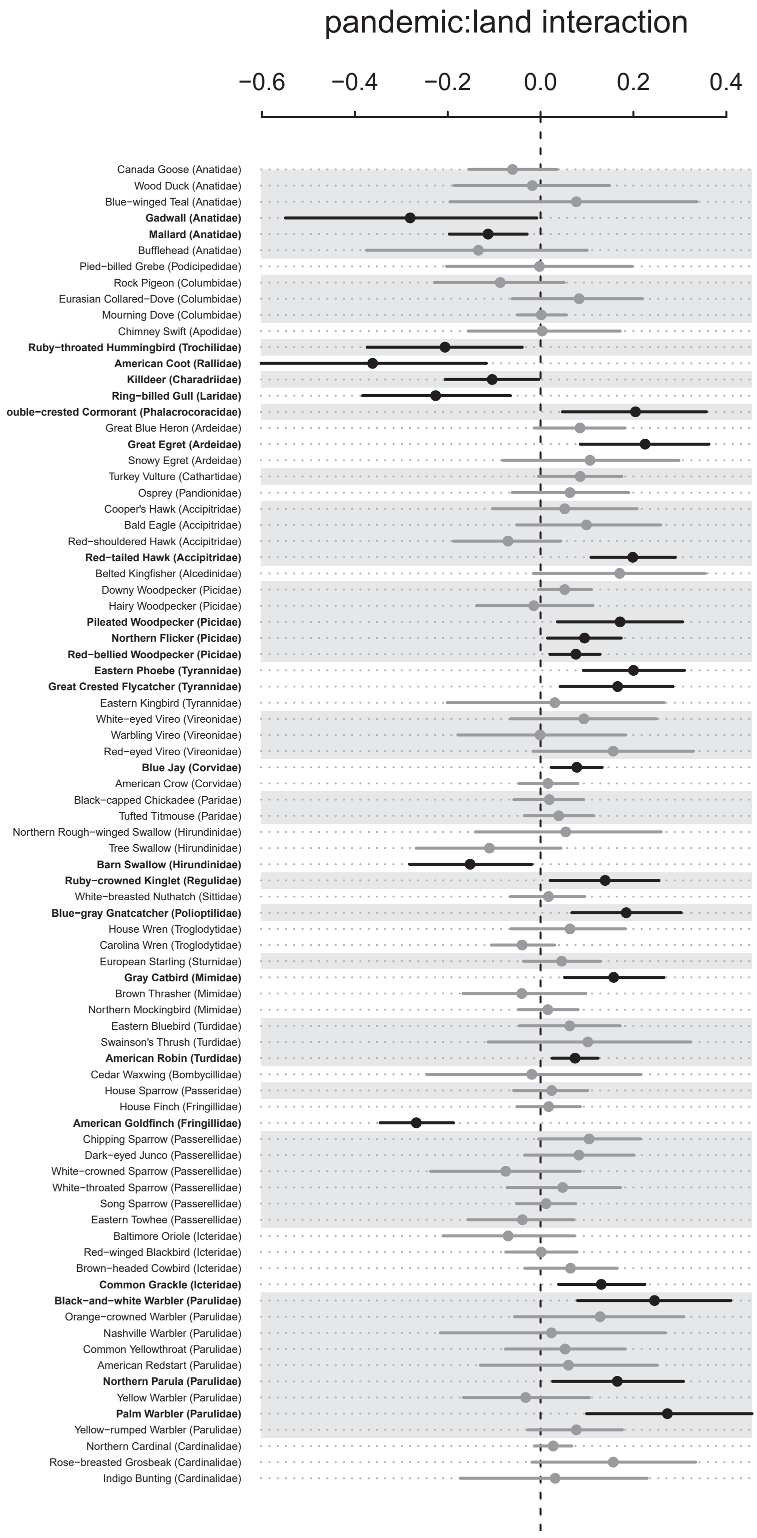

pandemic:land interaction
−0.6
−0.4
−0.2
0.0
0.2
0.4
Canada Goose (Anatidae)
Wood Duck (Anatidae)
Blue−winged Teal (Anatidae)
Gadwall (Anatidae)
Mallard (Anatidae)
Bufflehead (Anatidae)
Pied−billed Grebe (Podicipedidae)
Rock Pigeon (Columbidae)
Eurasian Collared−Dove (Columbidae)
Mourning Dove (Columbidae)
Chimney Swift (Apodidae)
Ruby−throated Hummingbird (Trochilidae)
American Coot (Rallidae)
Killdeer (Charadriidae)
Ring−billed Gull (Laridae)
ouble−crested Cormorant (Phalacrocoracidae)
Great Blue Heron (Ardeidae)
Great Egret (Ardeidae)
Snowy Egret (Ardeidae)
Turkey Vulture (Cathartidae)
Osprey (Pandionidae)
Cooper's Hawk (Accipitridae)
Bald Eagle (Accipitridae)
Red−shouldered Hawk (Accipitridae)
Red−tailed Hawk (Accipitridae)
Belted Kingfisher (Alcedinidae)
Downy Woodpecker (Picidae)
Hairy Woodpecker (Picidae)
Pileated Woodpecker (Picidae)
Northern Flicker (Picidae)
Red−bellied Woodpecker (Picidae)
Eastern Phoebe (Tyrannidae)
Great Crested Flycatcher (Tyrannidae)
Eastern Kingbird (Tyrannidae)
White−eyed Vireo (Vireonidae)
Warbling Vireo (Vireonidae)
Red−eyed Vireo (Vireonidae)
Blue Jay (Corvidae)
American Crow (Corvidae)
Black−capped Chickadee (Paridae)
Tufted Titmouse (Paridae)
Northern Rough−winged Swallow (Hirundinidae)
Tree Swallow (Hirundinidae)
Barn Swallow (Hirundinidae)
Ruby−crowned Kinglet (Regulidae)
White−breasted Nuthatch (Sittidae)
Blue−gray Gnatcatcher (Polioptilidae)
House Wren (Troglodytidae)
Carolina Wren (Troglodytidae)
European Starling (Sturnidae)
Gray Catbird (Mimidae)
Brown Thrasher (Mimidae)
Northern Mockingbird (Mimidae)
Eastern Bluebird (Turdidae)
Swainson's Thrush (Turdidae)
American Robin (Turdidae)
Cedar Waxwing (Bombycillidae)
House Sparrow (Passeridae)
House Finch (Fringillidae)
American Goldfinch (Fringillidae)
Chipping Sparrow (Passerellidae)
Dark−eyed Junco (Passerellidae)
White−crowned Sparrow (Passerellidae)
White−throated Sparrow (Passerellidae)
Song Sparrow (Passerellidae)
Eastern Towhee (Passerellidae)
Baltimore Oriole (Icteridae)
Red−winged Blackbird (Icteridae)
Brown−headed Cowbird (Icteridae)
Common Grackle (Icteridae)
Black−and−white Warbler (Parulidae)
Orange−crowned Warbler (Parulidae)
Nashville Warbler (Parulidae)
Common Yellowthroat (Parulidae)
American Redstart (Parulidae)
Northern Parula (Parulidae)
Yellow Warbler (Parulidae)
Palm Warbler (Parulidae)
Yellow−rumped Warbler (Parulidae)
Northern Cardinal (Cardinalidae)
Rose−breasted Grosbeak (Cardinalidae)
Indigo Bunting (Cardinalidae)

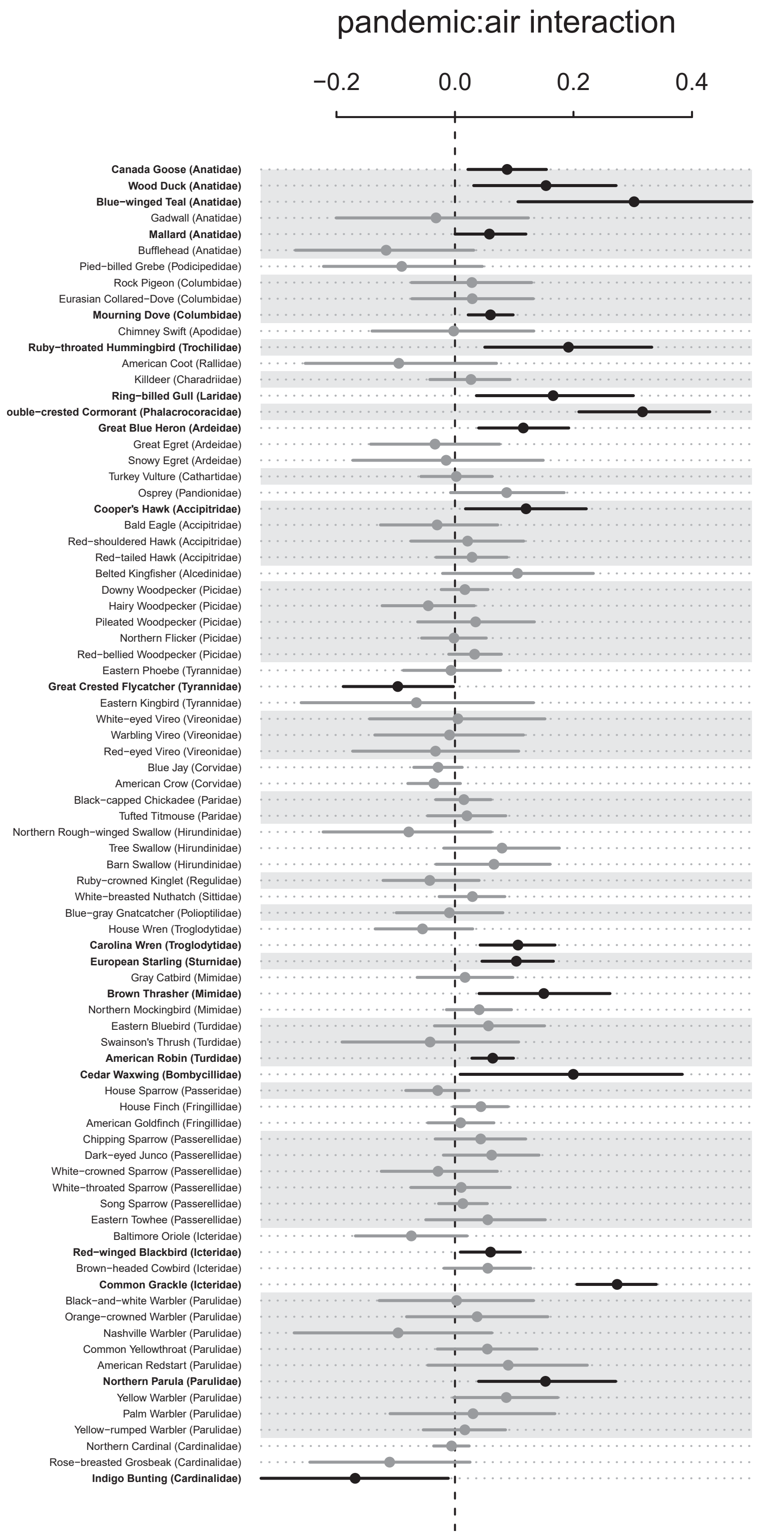
pandemic:air interaction
−0.2
0.0
0.2
0.4
Canada Goose (Anatidae)
Wood Duck (Anatidae)
Blue−winged Teal (Anatidae)
Gadwall (Anatidae)
Mallard (Anatidae)
Bufflehead (Anatidae)
Pied−billed Grebe (Podicipedidae)
Rock Pigeon (Columbidae)
Eurasian Collared−Dove (Columbidae)
Mourning Dove (Columbidae)
Chimney Swift (Apodidae)
Ruby−throated Hummingbird (Trochilidae)
American Coot (Rallidae)
Killdeer (Charadriidae)
Ring−billed Gull (Laridae)
ouble−crested Cormorant (Phalacrocoracidae)
Great Blue Heron (Ardeidae)
Great Egret (Ardeidae)
Snowy Egret (Ardeidae)
Turkey Vulture (Cathartidae)
Osprey (Pandionidae)
Cooper's Hawk (Accipitridae)
Bald Eagle (Accipitridae)
Red−shouldered Hawk (Accipitridae)
Red−tailed Hawk (Accipitridae)
Belted Kingfisher (Alcedinidae)
Downy Woodpecker (Picidae)
Hairy Woodpecker (Picidae)
Pileated Woodpecker (Picidae)
Northern Flicker (Picidae)
Red−bellied Woodpecker (Picidae)
Eastern Phoebe (Tyrannidae)
Great Crested Flycatcher (Tyrannidae)
Eastern Kingbird (Tyrannidae)
White−eyed Vireo (Vireonidae)
Warbling Vireo (Vireonidae)
Red−eyed Vireo (Vireonidae)
Blue Jay (Corvidae)
American Crow (Corvidae)
Black−capped Chickadee (Paridae)
Tufted Titmouse (Paridae)
Northern Rough−winged Swallow (Hirundinidae)
Tree Swallow (Hirundinidae)
Barn Swallow (Hirundinidae)
Ruby−crowned Kinglet (Regulidae)
White−breasted Nuthatch (Sittidae)
Blue−gray Gnatcatcher (Polioptilidae)
House Wren (Troglodytidae)
Carolina Wren (Troglodytidae)
European Starling (Sturnidae)
Gray Catbird (Mimidae)
Brown Thrasher (Mimidae)
Northern Mockingbird (Mimidae)
Eastern Bluebird (Turdidae)
Swainson's Thrush (Turdidae)
American Robin (Turdidae)
Cedar Waxwing (Bombycillidae)
House Sparrow (Passeridae)
House Finch (Fringillidae)
American Goldfinch (Fringillidae)
Chipping Sparrow (Passerellidae)
Dark−eyed Junco (Passerellidae)
White−crowned Sparrow (Passerellidae)
White−throated Sparrow (Passerellidae)
Song Sparrow (Passerellidae)
Eastern Towhee (Passerellidae)
Baltimore Oriole (Icteridae)
Red−winged Blackbird (Icteridae)
Brown−headed Cowbird (Icteridae)
Common Grackle (Icteridae)
Black−and−white Warbler (Parulidae)
Orange−crowned Warbler (Parulidae)
Nashville Warbler (Parulidae)
Common Yellowthroat (Parulidae)
American Redstart (Parulidae)
Northern Parula (Parulidae)
Yellow Warbler (Parulidae)
Palm Warbler (Parulidae)
Yellow−rumped Warbler (Parulidae)
Northern Cardinal (Cardinalidae)
Rose−breasted Grosbeak (Cardinalidae)
Indigo Bunting (Cardinalidae)

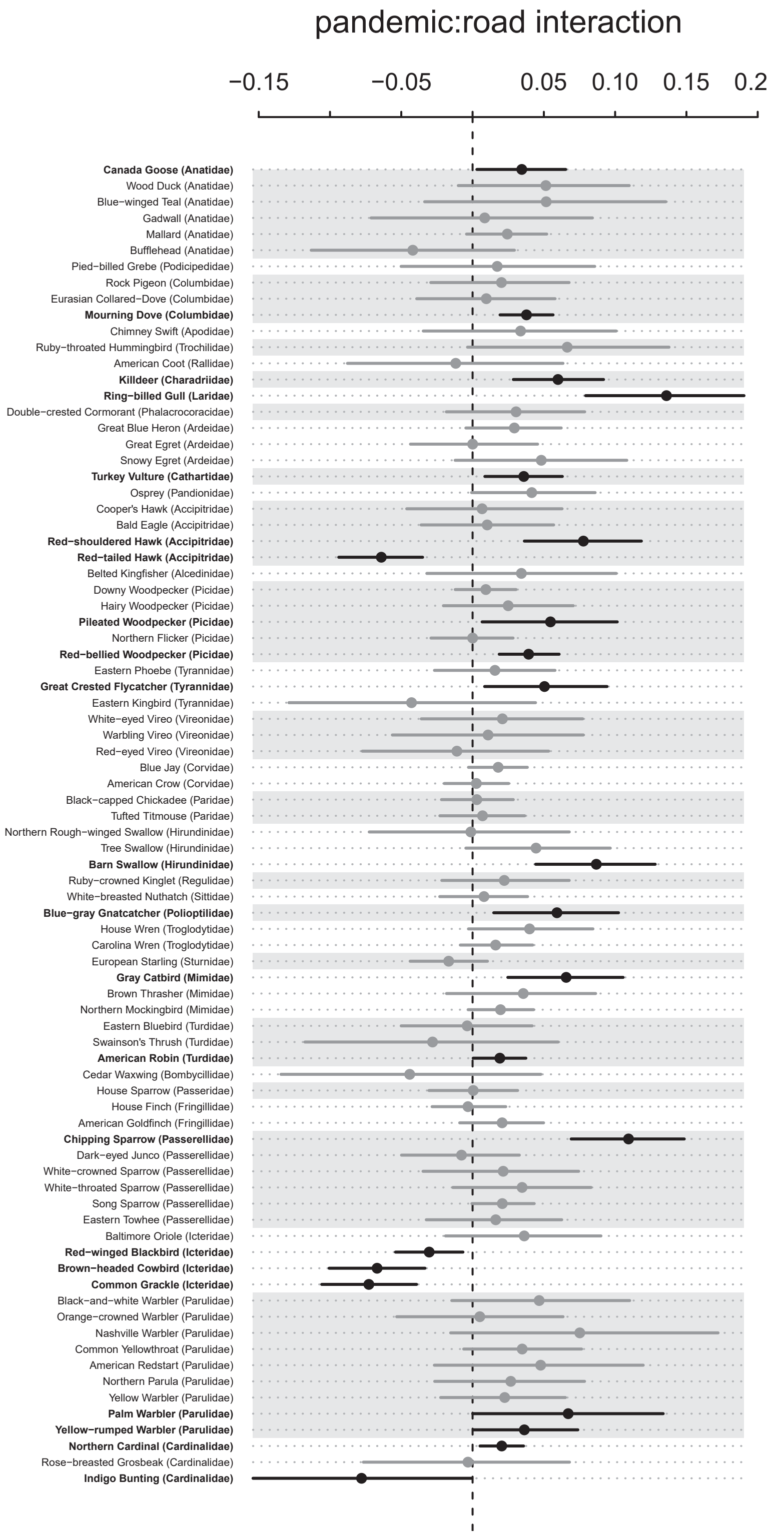

pandemic:road interaction
−0.15
−0.05
0.05
0.10
0.15
0.2
Canada Goose (Anatidae)
Wood Duck (Anatidae)
Blue−winged Teal (Anatidae)
Gadwall (Anatidae)
Mallard (Anatidae)
Bufflehead (Anatidae)
Pied−billed Grebe (Podicipedidae)
Rock Pigeon (Columbidae)
Eurasian Collared−Dove (Columbidae)
Mourning Dove (Columbidae)
Chimney Swift (Apodidae)
Ruby−throated Hummingbird (Trochilidae)
American Coot (Rallidae)
Killdeer (Charadriidae)
Ring−billed Gull (Laridae)
Double−crested Cormorant (Phalacrocoracidae)
Great Blue Heron (Ardeidae)
Great Egret (Ardeidae)
Snowy Egret (Ardeidae)
Turkey Vulture (Cathartidae)
Osprey (Pandionidae)
Cooper's Hawk (Accipitridae)
Bald Eagle (Accipitridae)
Red−shouldered Hawk (Accipitridae)
Red−tailed Hawk (Accipitridae)
Belted Kingfisher (Alcedinidae)
Downy Woodpecker (Picidae)
Hairy Woodpecker (Picidae)
Pileated Woodpecker (Picidae)
Northern Flicker (Picidae)
Red−bellied Woodpecker (Picidae)
Eastern Phoebe (Tyrannidae)
Great Crested Flycatcher (Tyrannidae)
Eastern Kingbird (Tyrannidae)
White−eyed Vireo (Vireonidae)
Warbling Vireo (Vireonidae)
Red−eyed Vireo (Vireonidae)
Blue Jay (Corvidae)
American Crow (Corvidae)
Black−capped Chickadee (Paridae)
Tufted Titmouse (Paridae)
Northern Rough−winged Swallow (Hirundinidae)
Tree Swallow (Hirundinidae)
Barn Swallow (Hirundinidae)
Ruby−crowned Kinglet (Regulidae)
White−breasted Nuthatch (Sittidae)
Blue−gray Gnatcatcher (Polioptilidae)
House Wren (Troglodytidae)
Carolina Wren (Troglodytidae)
European Starling (Sturnidae)
Gray Catbird (Mimidae)
Brown Thrasher (Mimidae)
Northern Mockingbird (Mimidae)
Eastern Bluebird (Turdidae)
Swainson's Thrush (Turdidae)
American Robin (Turdidae)
Cedar Waxwing (Bombycillidae)
House Sparrow (Passeridae)
House Finch (Fringillidae)
American Goldfinch (Fringillidae)
Chipping Sparrow (Passerellidae)
Dark−eyed Junco (Passerellidae)
White−crowned Sparrow (Passerellidae)
White−throated Sparrow (Passerellidae)
Song Sparrow (Passerellidae)
Eastern Towhee (Passerellidae)
Baltimore Oriole (Icteridae)
Red−winged Blackbird (Icteridae)
Brown−headed Cowbird (Icteridae)
Common Grackle (Icteridae)
Black−and−white Warbler (Parulidae)
Orange−crowned Warbler (Parulidae)
Nashville Warbler (Parulidae)
Common Yellowthroat (Parulidae)
American Redstart (Parulidae)
Northern Parula (Parulidae)
Yellow Warbler (Parulidae)
Palm Warbler (Parulidae)
Yellow−rumped Warbler (Parulidae)
Northern Cardinal (Cardinalidae)
Rose−breasted Grosbeak (Cardinalidae)
Indigo Bunting (Cardinalidae)

Fig. S11. (multi-page figure beginning on the next page)

Interactions between period (pandemic/pre-pandemic) and focal variables describing pandemic-altered areas. Marginal predicted mean count (lines or points) including 95% credible interval (shaded areas or error bars) of the effect of the focal variable on bird counts during the COVID-19 pandemic (purple) compared to the pre-pandemic baseline period (orange) for all 82 North American focal species (arranged alphabetically by common name). To facilitate visualization of non-zero changes in relationship, plots are only included for interaction coefficients with 95% credible intervals not containing zero and are arranged as follows: (top right) overlap between peak migration and peak travel reductions, (center left) percent change in human mobility during pandemic, (center right) urban versus rural habitat, (bottom left) distance to airport, and (bottom right) distance to major road. Changes to human activity during the pandemic increase from left to right on each panel.

American Coot

Fulica americana

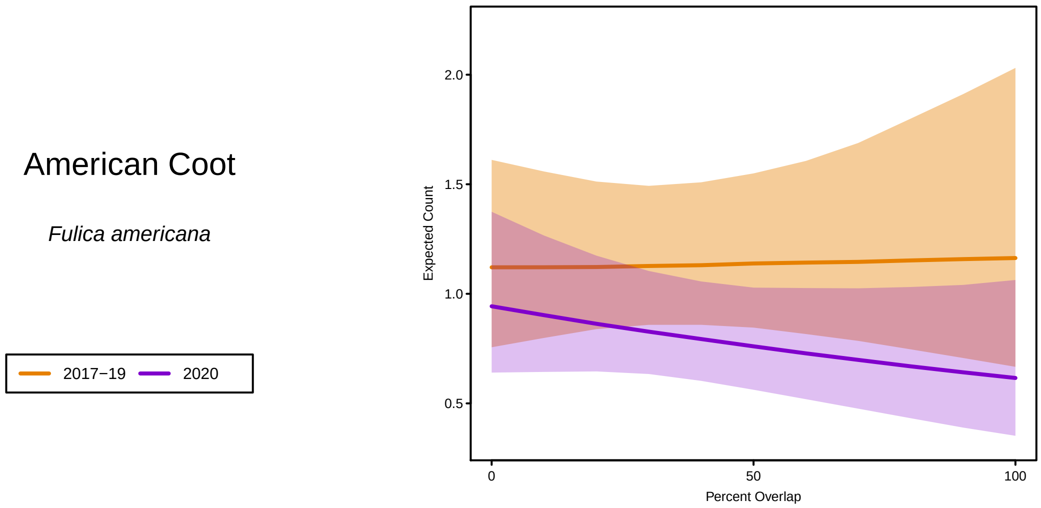

posterior distribution 95% CI
for interaction with
delta_traf includes zero

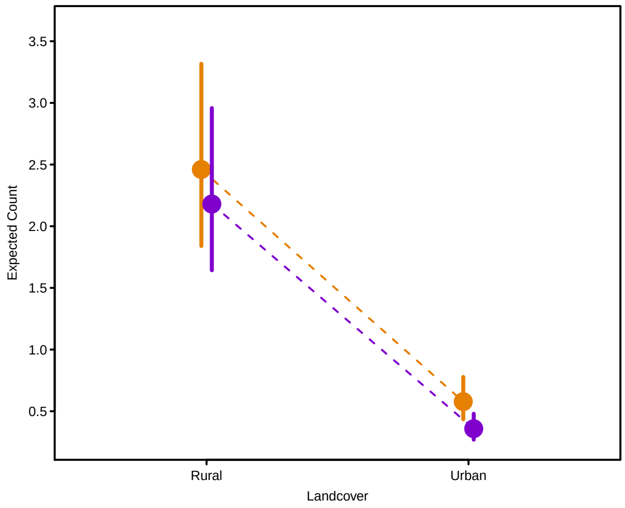

posterior distribution 95% CI
for interaction with
air includes zero

posterior distribution 95% CI
for interaction with
road includes zero

American Crow

Corvus brachyrhynchos

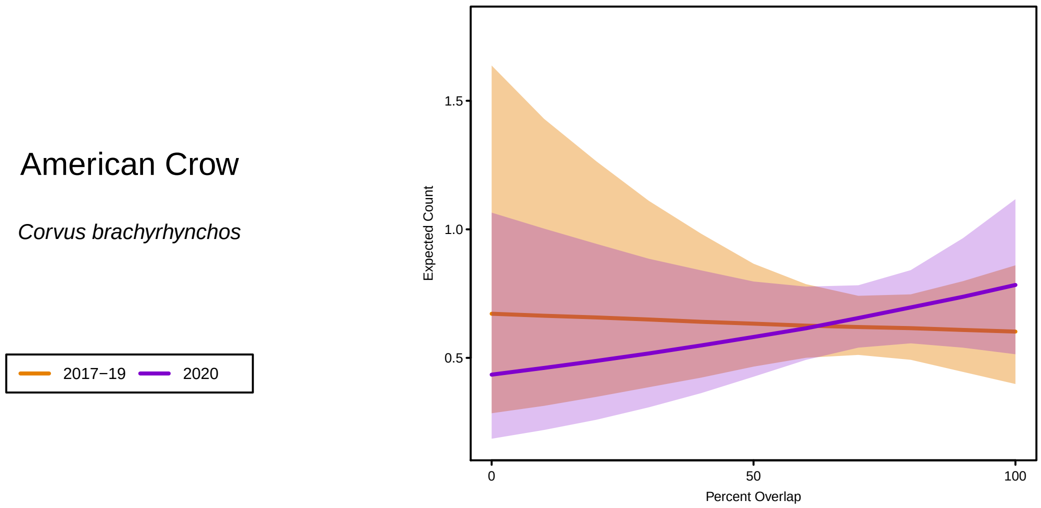

posterior distribution 95% CI
for interaction with
delta_traf includes zero

posterior distribution 95% CI
for interaction with
land includes zero

posterior distribution 95% CI
for interaction with
air includes zero

posterior distribution 95% CI
for interaction with
road includes zero

American Goldfinch

Spinus tristis

2017−19 2020

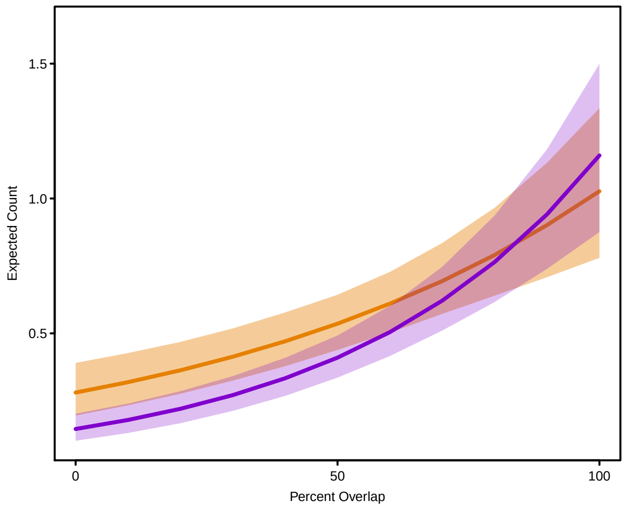

posterior distribution 95% CI
for interaction with
delta_traf includes zero

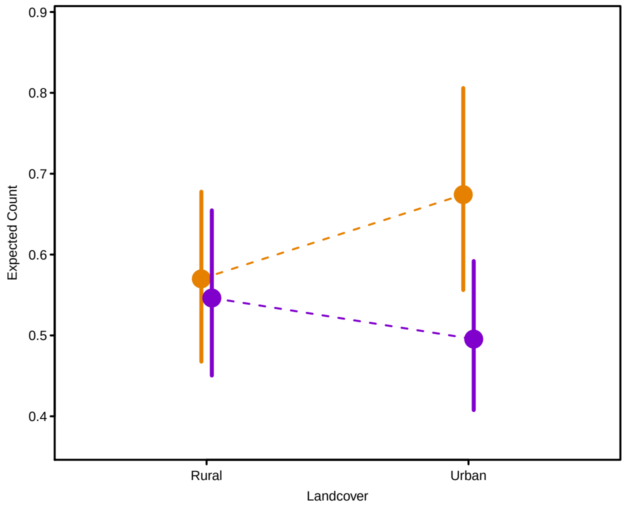

posterior distribution 95% CI
for interaction with
air includes zero

posterior distribution 95% CI
for interaction with
road includes zero

American Redstart

Setophaga ruticilla

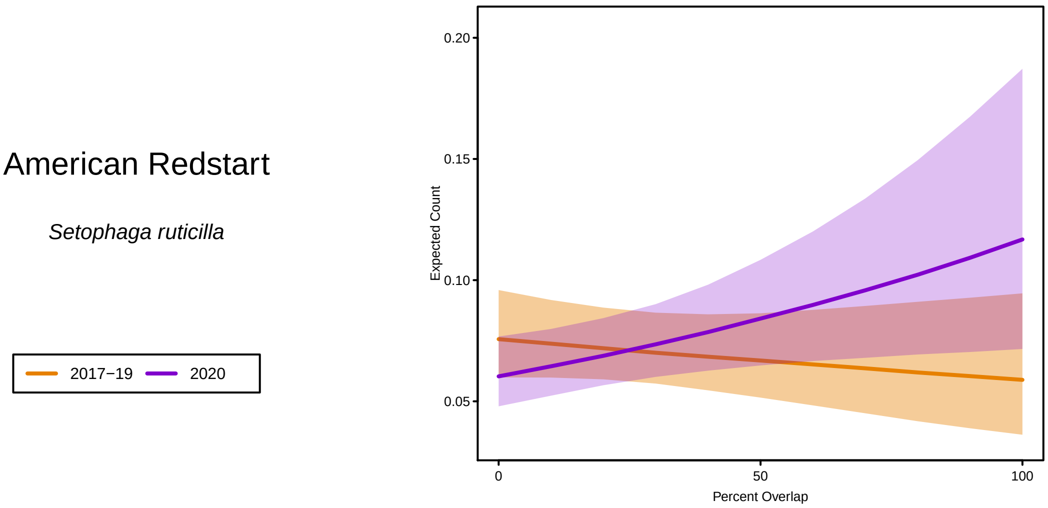

posterior distribution 95% CI
for interaction with
delta_traf includes zero

posterior distribution 95% CI
for interaction with
land includes zero

posterior distribution 95% CI
for interaction with
air includes zero

posterior distribution 95% CI
for interaction with
road includes zero

American Robin

Turdus migratorius

2017−19 2020

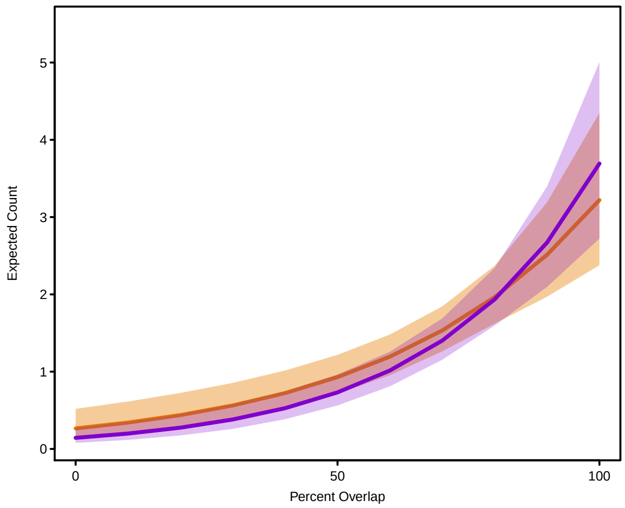

posterior distribution 95% CI
for interaction with
delta_traf includes zero

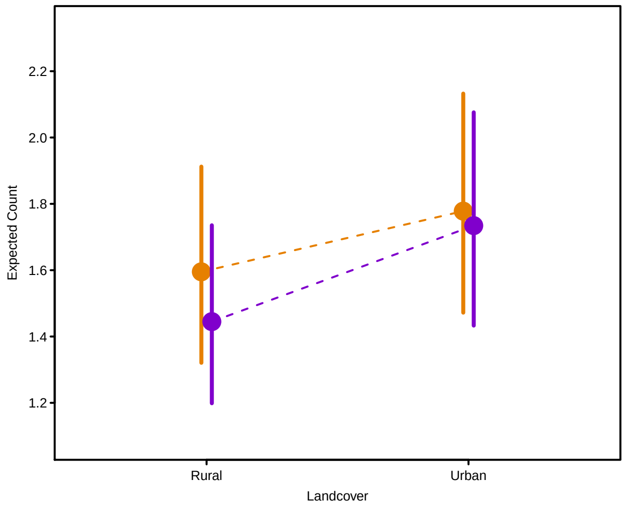

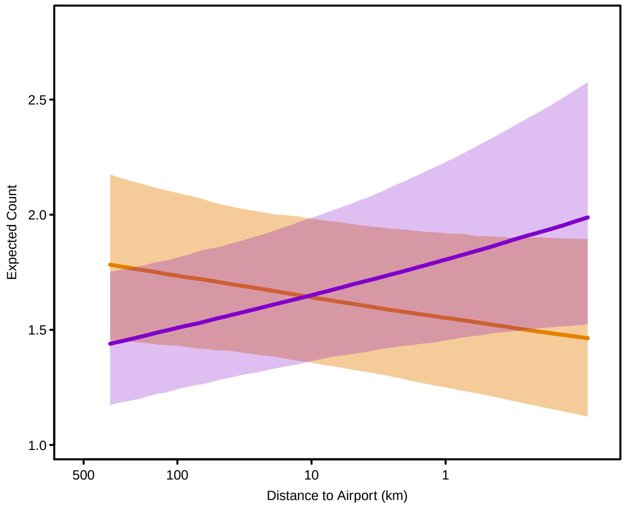

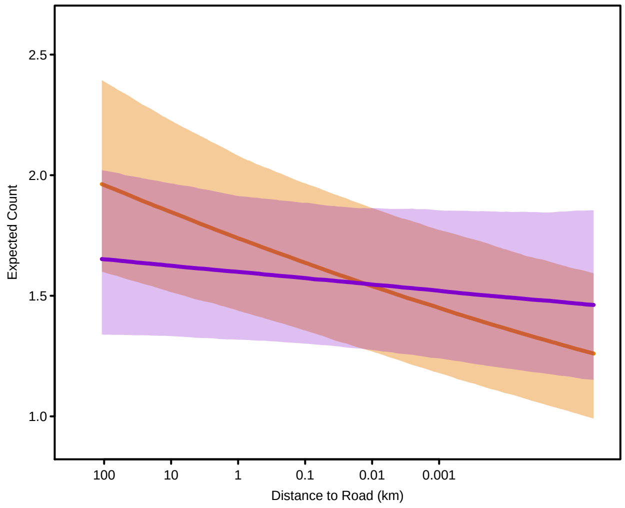

Bald Eagle

Haliaeetus leucocephalus

posterior distribution 95% CI
for interaction with
overlap includes zero

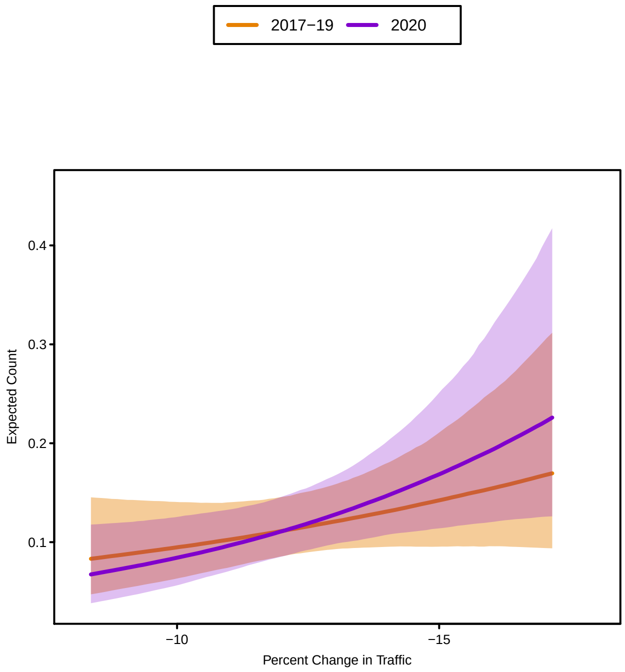

posterior distribution 95% CI
for interaction with
land includes zero

posterior distribution 95% CI
for interaction with
air includes zero

posterior distribution 95% CI
for interaction with
road includes zero

Baltimore Oriole

Icterus galbula

2017−19 2020

posterior distribution 95% CI
for interaction with
overlap includes zero

posterior distribution 95% CI
for interaction with
delta_traf includes zero

posterior distribution 95% CI
for interaction with
land includes zero

posterior distribution 95% CI
for interaction with
air includes zero

posterior distribution 95% CI
for interaction with
road includes zero

Barn Swallow

Hirundo rustica

2017−19 — 2020

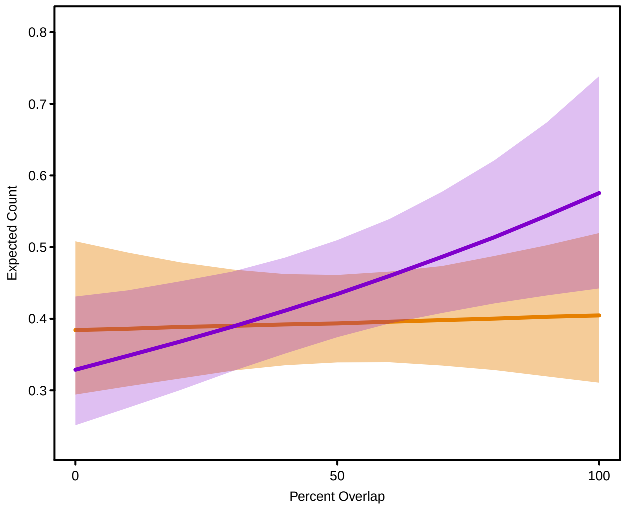

posterior distribution 95% CI
for interaction with
delta_traf includes zero

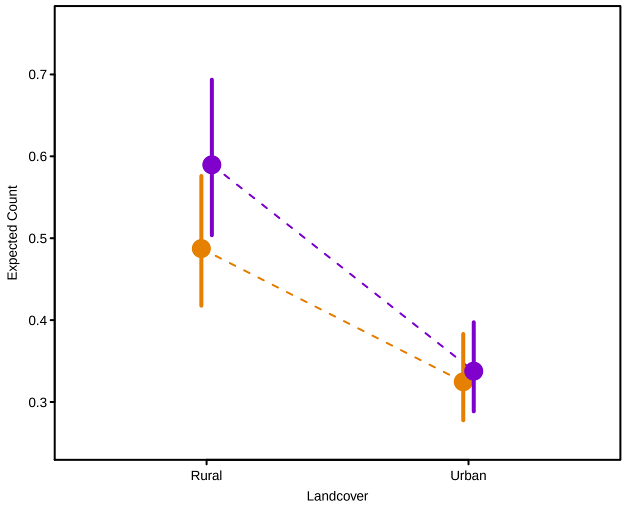

posterior distribution 95% CI
for interaction with
air includes zero

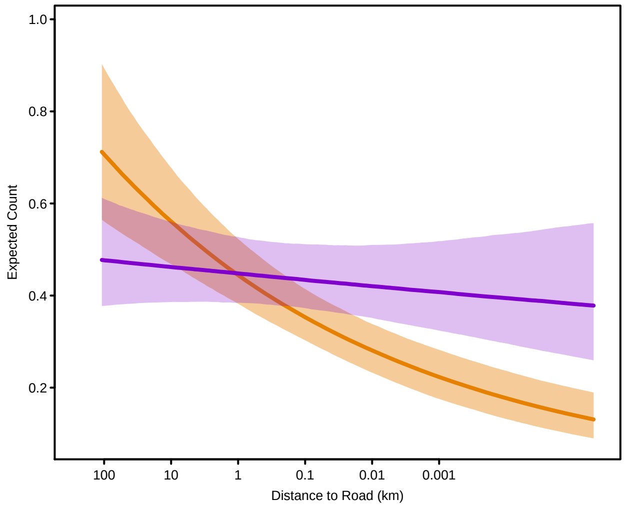

Belted Kingfisher

Megaceryle alcyon

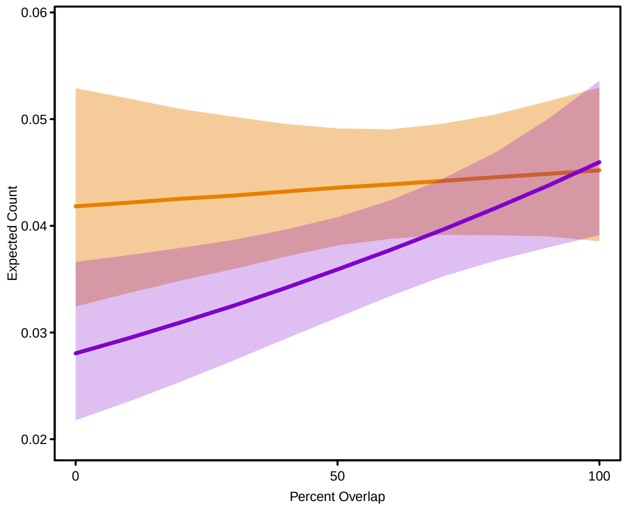

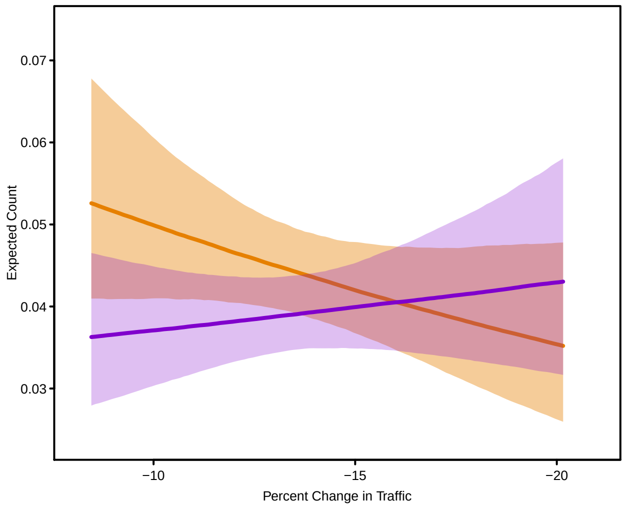

2017−19 2020

posterior distribution 95% CI
for interaction with
land includes zero

posterior distribution 95% CI
for interaction with
air includes zero

posterior distribution 95% CI
for interaction with
road includes zero

Black−and−white Warbler

Mniotilta varia

2017−19 2020

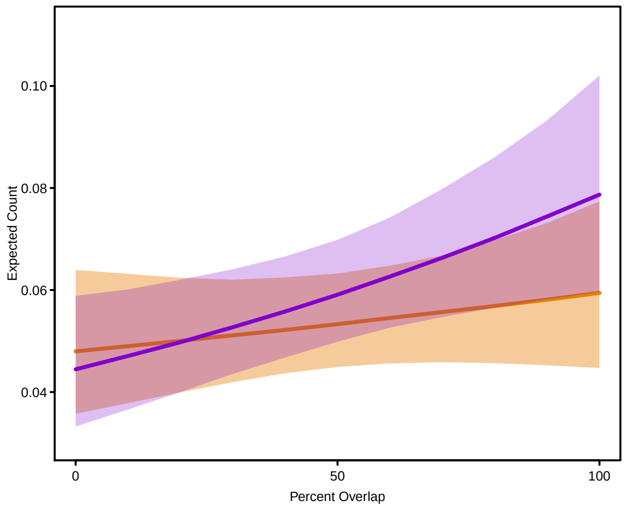

posterior distribution 95% CI
for interaction with
delta_traf includes zero

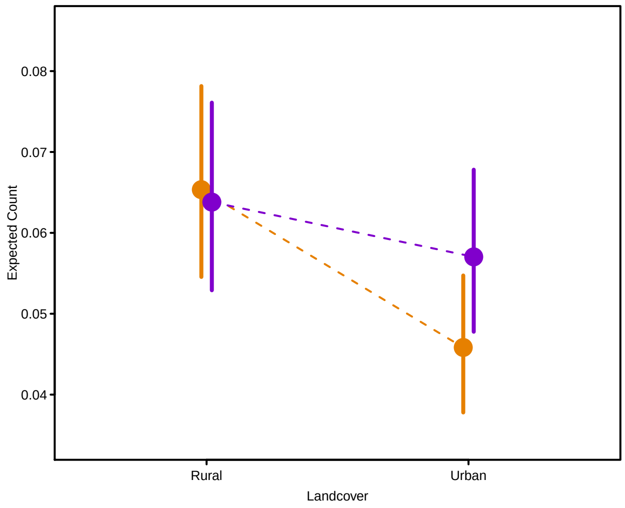

posterior distribution 95% CI
for interaction with
air includes zero

posterior distribution 95% CI
for interaction with
road includes zero

Black−capped Chickadee

Poecile atricapillus

2017−19 2020

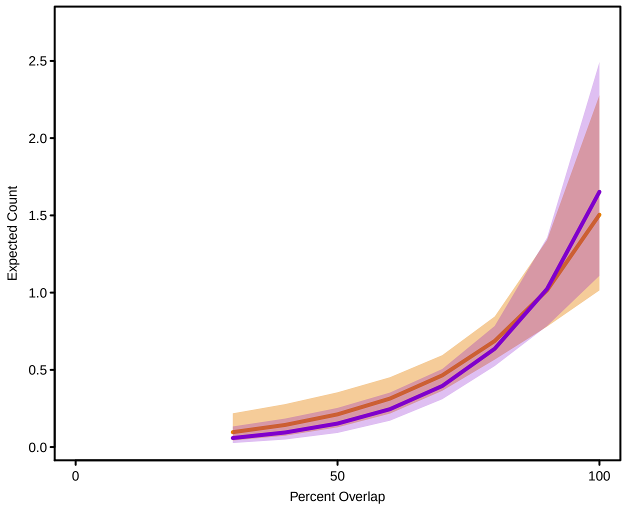

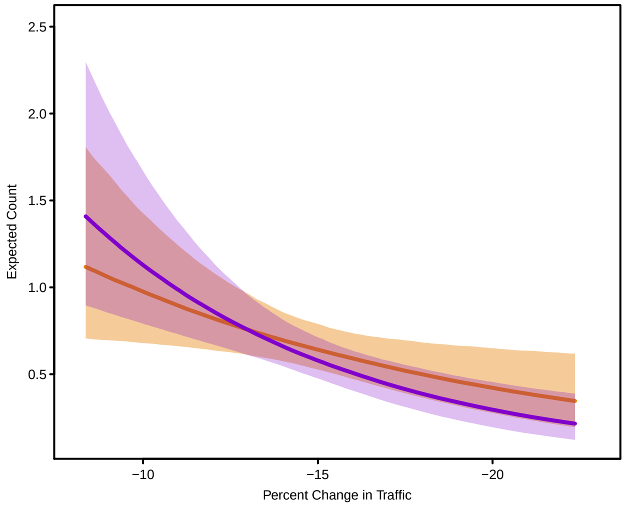

posterior distribution 95% CI
for interaction with
land includes zero

posterior distribution 95% CI
for interaction with
air includes zero

posterior distribution 95% CI
for interaction with
road includes zero

Blue−gray Gnatcatcher

Polioptila caerulea

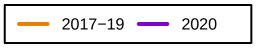

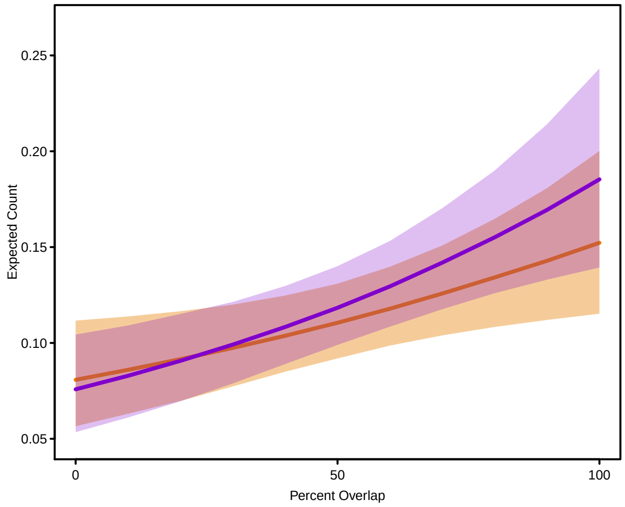

posterior distribution 95% CI
for interaction with
delta_traf includes zero

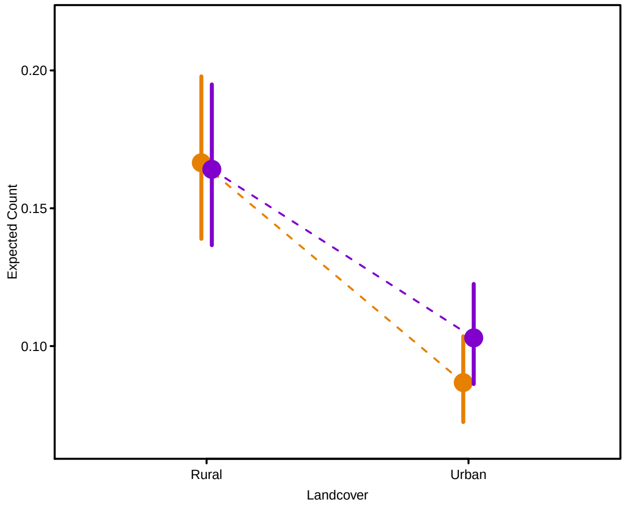

posterior distribution 95% CI
for interaction with
air includes zero

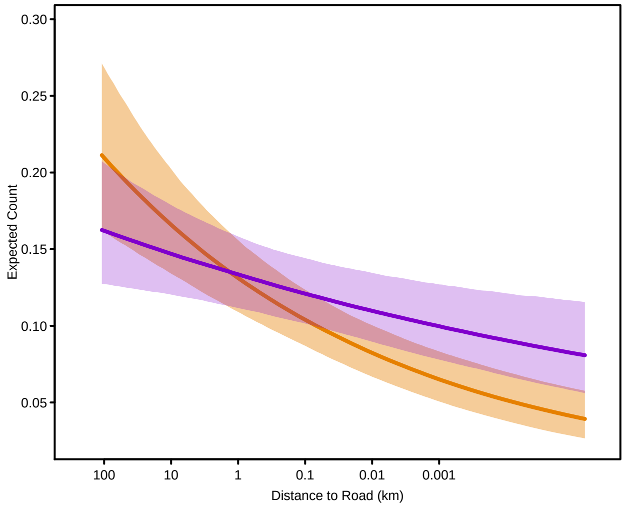

Blue−winged Teal

Spatula discors

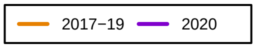

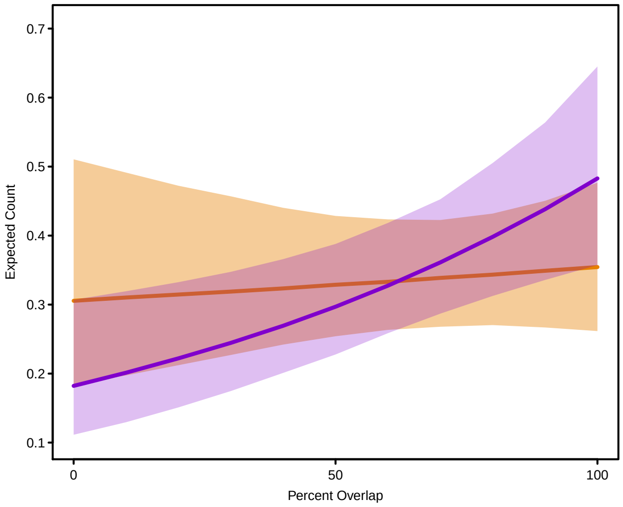

posterior distribution 95% CI
for interaction with
delta_traf includes zero

posterior distribution 95% CI
for interaction with
land includes zero

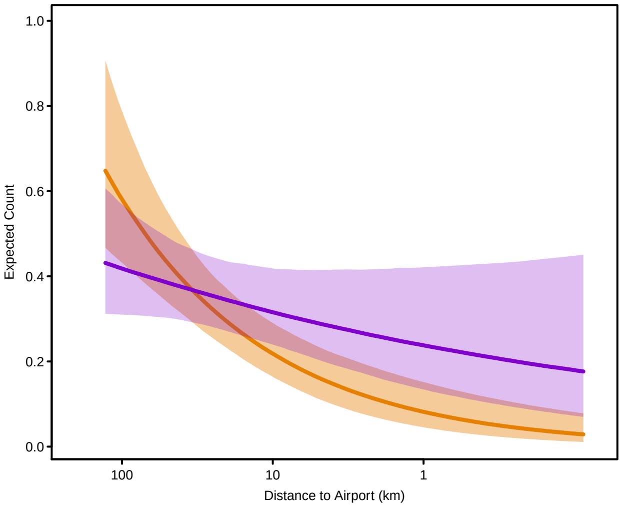

posterior distribution 95% CI
for interaction with
road includes zero

Blue Jay

Cyanocitta cristata

posterior distribution 95% CI
for interaction with
overlap includes zero

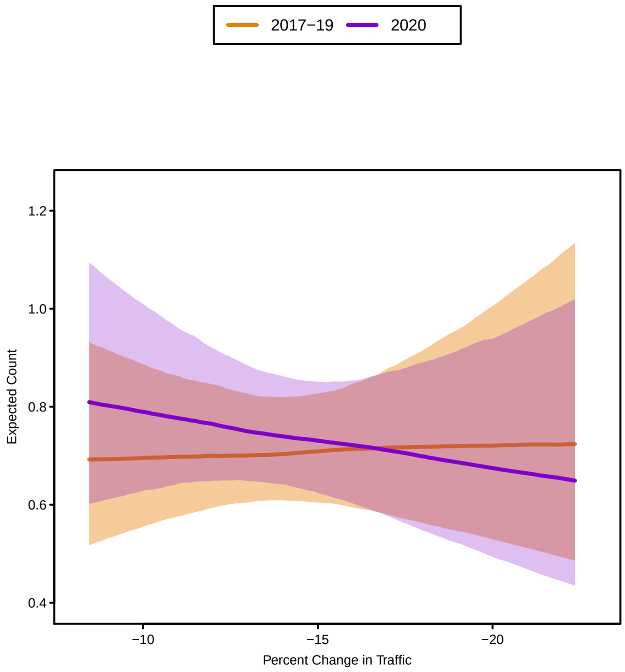

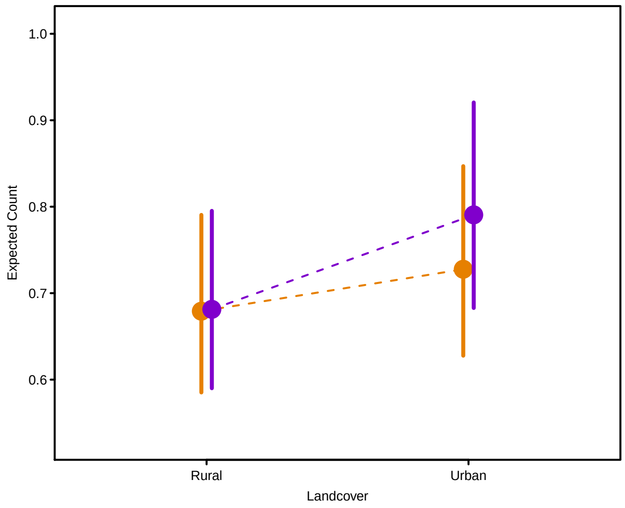

posterior distribution 95% CI
for interaction with
air includes zero

posterior distribution 95% CI
for interaction with
road includes zero

Brown−headed Cowbird

Molothrus ater

2017−19 2020

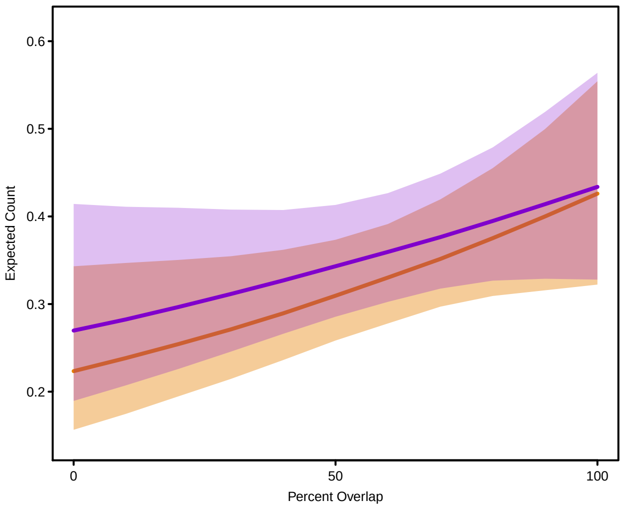

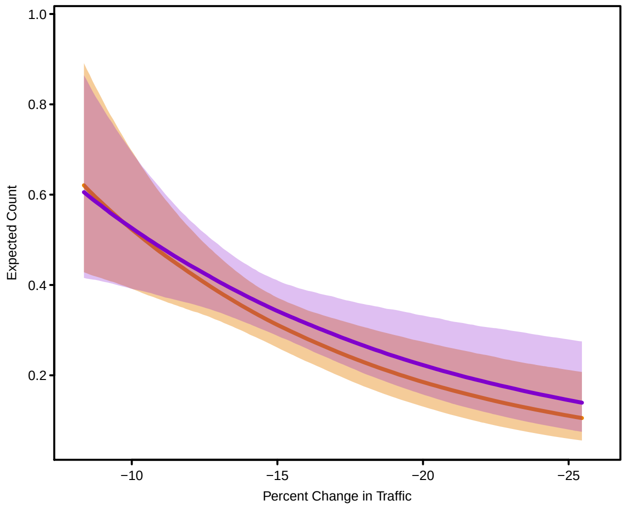

posterior distribution 95% CI
for interaction with
land includes zero

posterior distribution 95% CI
for interaction with
air includes zero

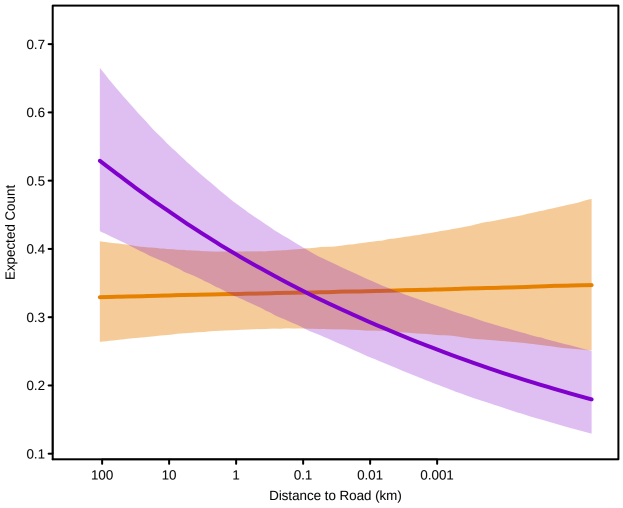

Brown Thrasher

Toxostoma rufum

posterior distribution 95% CI
for interaction with
overlap includes zero

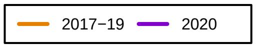

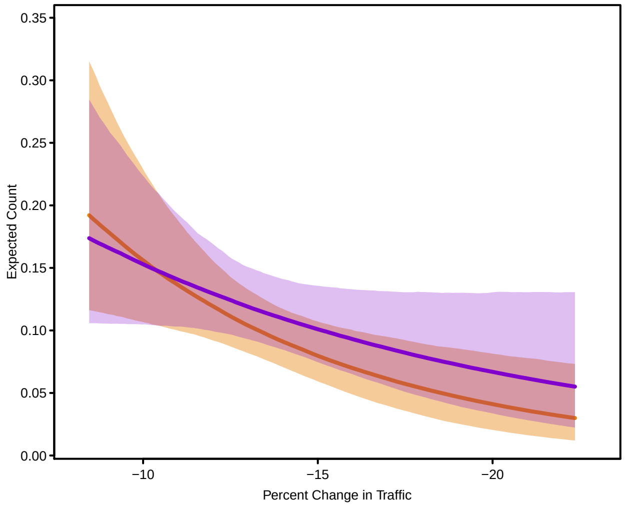

posterior distribution 95% CI
for interaction with
land includes zero

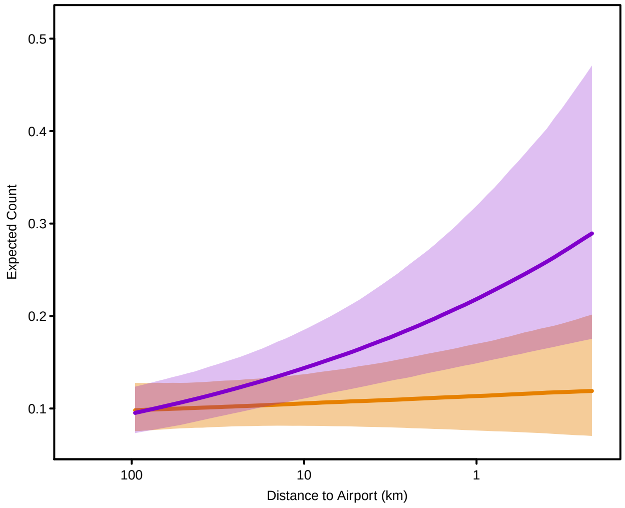

posterior distribution 95% CI
for interaction with
road includes zero

Bufflehead

Bucephala albeola

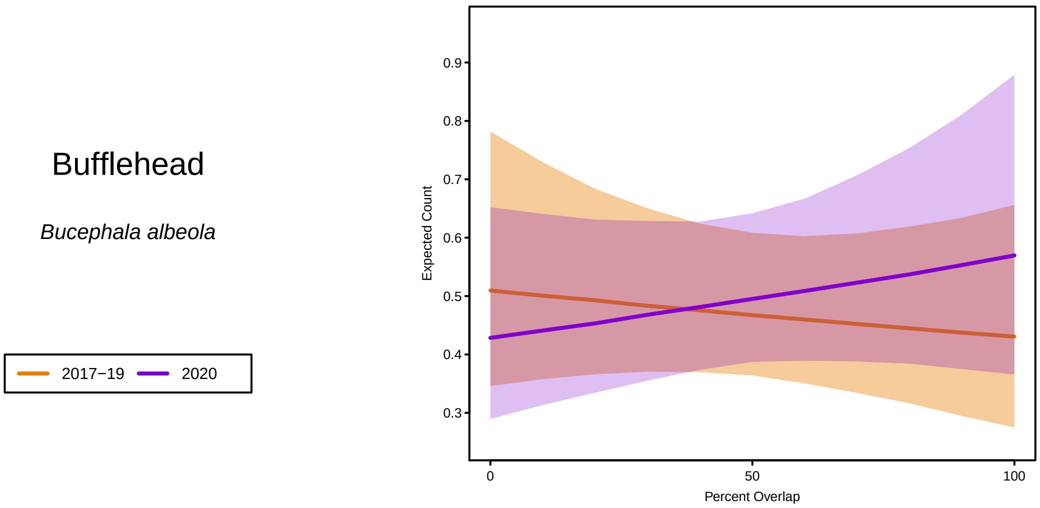

posterior distribution 95% CI
for interaction with
delta_traf includes zero

posterior distribution 95% CI
for interaction with
land includes zero

posterior distribution 95% CI
for interaction with
air includes zero

posterior distribution 95% CI
for interaction with
road includes zero

Canada Goose

Branta canadensis

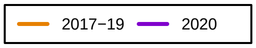

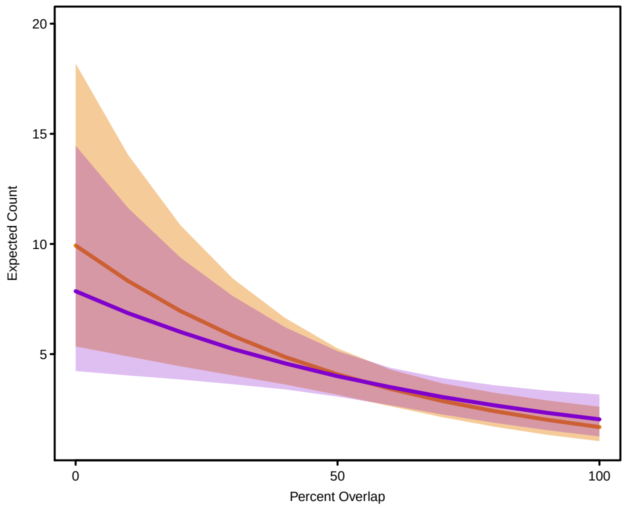

posterior distribution 95% CI
for interaction with
delta_traf includes zero

posterior distribution 95% CI
for interaction with
land includes zero

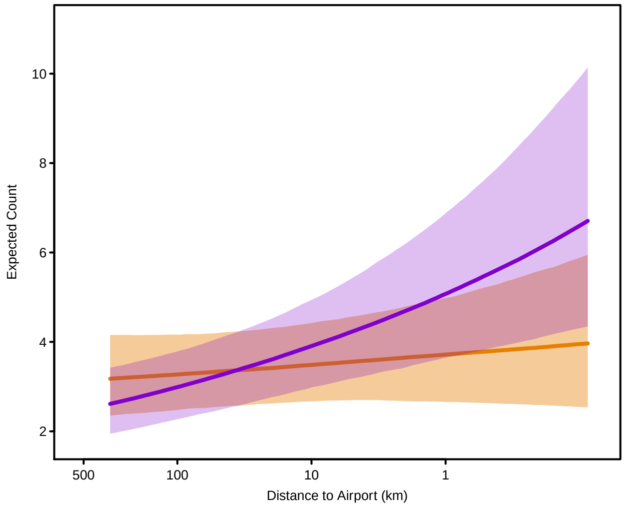

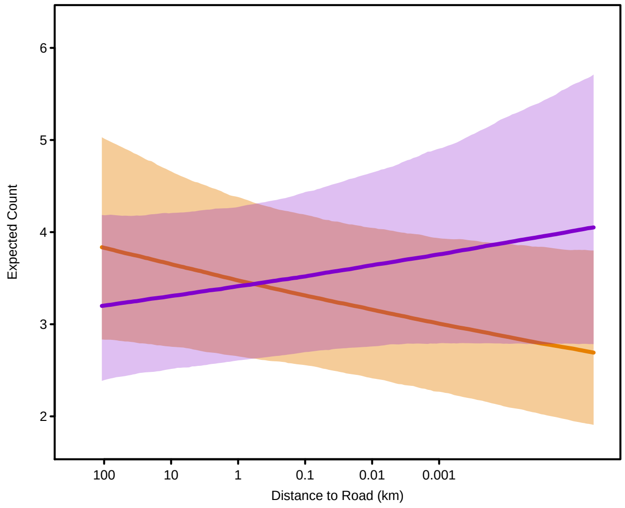

Carolina Wren

Thryothorus ludovicianus

posterior distribution 95% CI
for interaction with
overlap includes zero

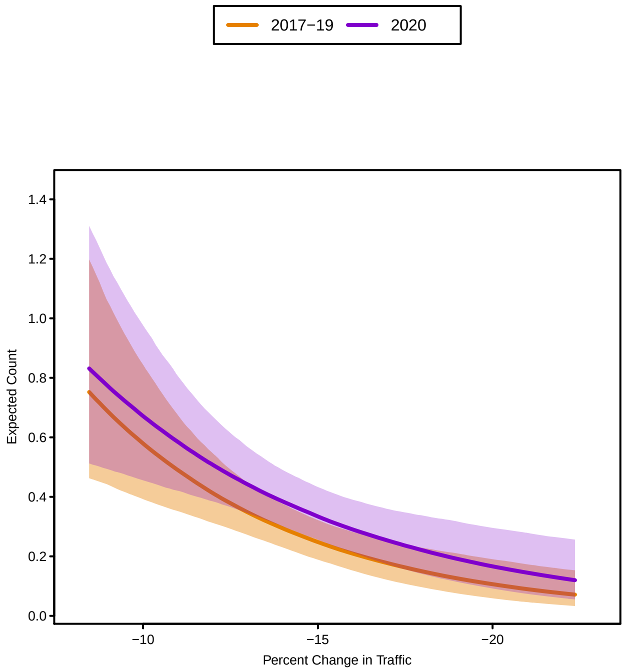

posterior distribution 95% CI
for interaction with
land includes zero

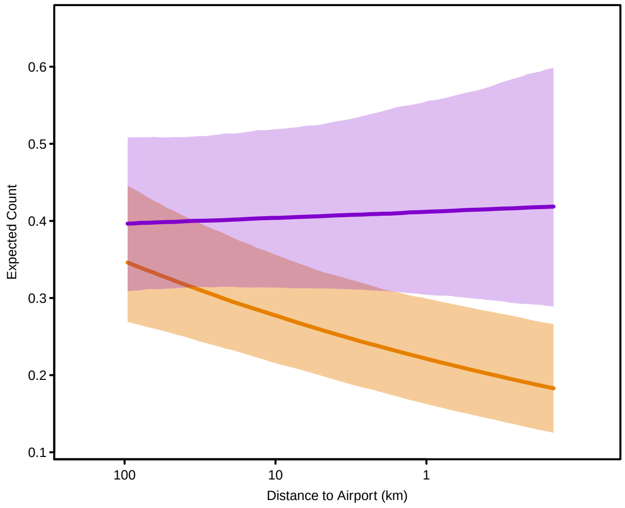

posterior distribution 95% CI
for interaction with
road includes zero

Cedar Waxwing

Bombycilla cedrorum

posterior distribution 95% CI
for interaction with
overlap includes zero

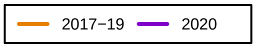

posterior distribution 95% CI
for interaction with
delta_traf includes zero

posterior distribution 95% CI
for interaction with
land includes zero

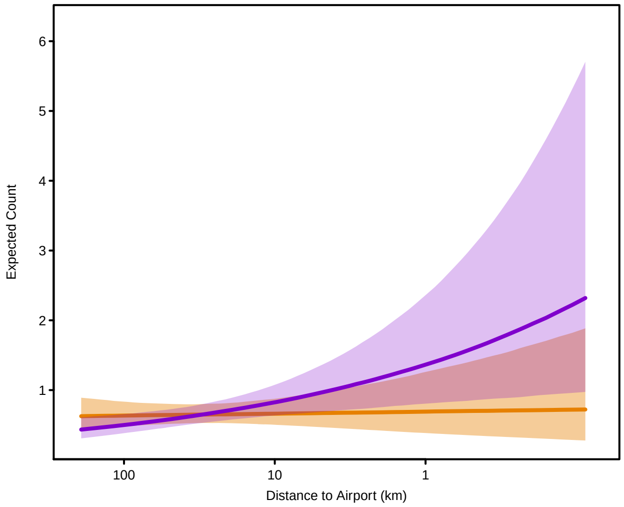

posterior distribution 95% CI
for interaction with
road includes zero

Chimney Swift

Chaetura pelagica

posterior distribution 95% CI
for interaction with
overlap includes zero

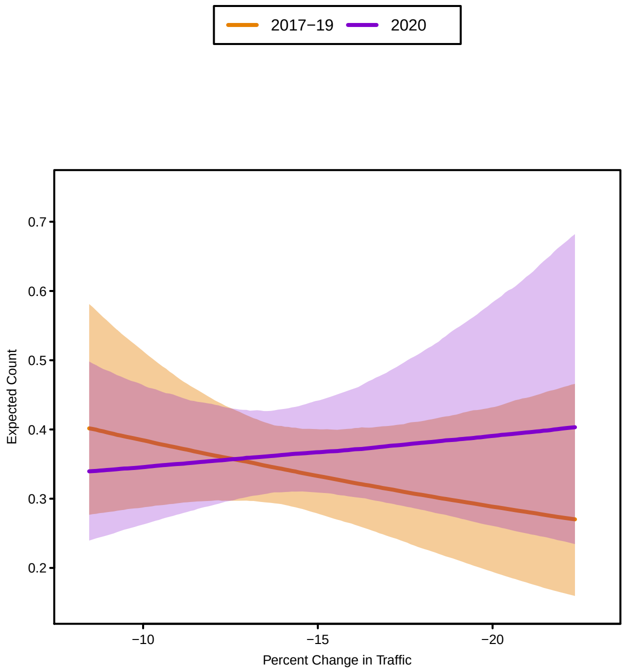

posterior distribution 95% CI
for interaction with
land includes zero

posterior distribution 95% CI
for interaction with
air includes zero

posterior distribution 95% CI
for interaction with
road includes zero

Chipping Sparrow

Spizella passerina

2017−19 2020

posterior distribution 95% CI
for interaction with
overlap includes zero

posterior distribution 95% CI
for interaction with
delta_traf includes zero

posterior distribution 95% CI
for interaction with
land includes zero

posterior distribution 95% CI
for interaction with
air includes zero

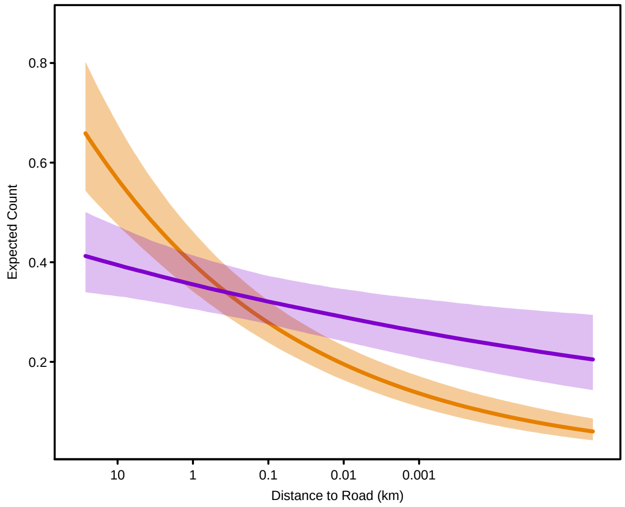

Common Grackle

Quiscalus quiscula

2017−19 — 2020

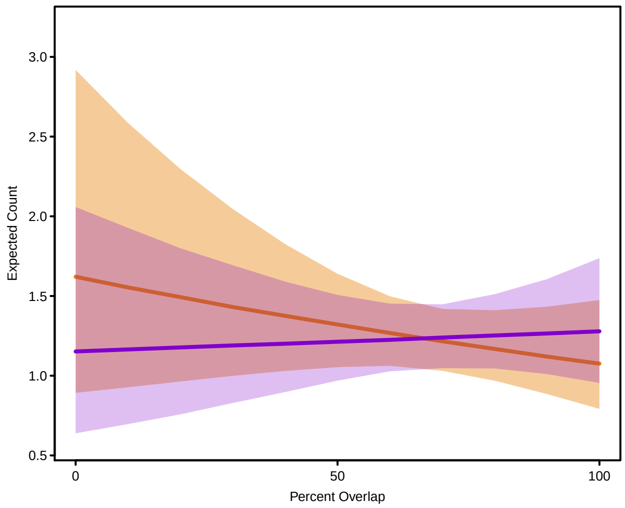

posterior distribution 95% CI
for interaction with
delta_traf includes zero

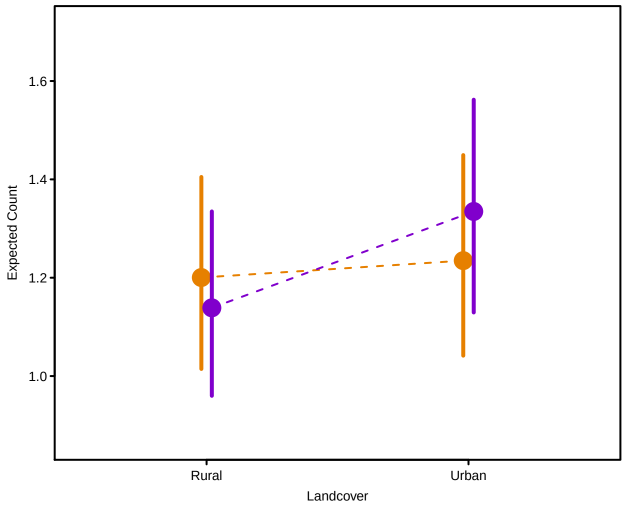

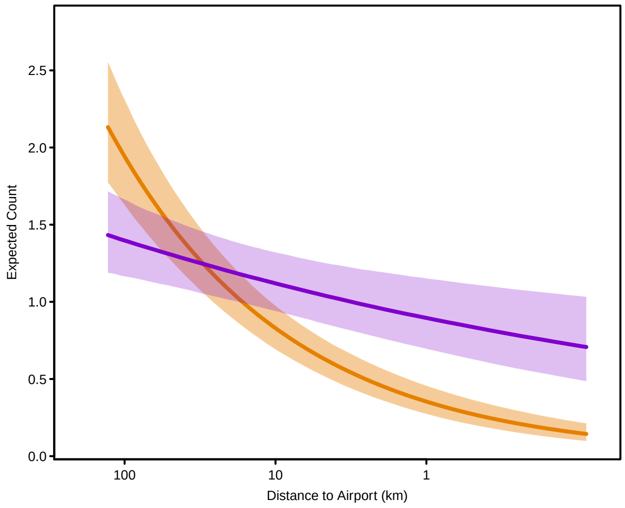

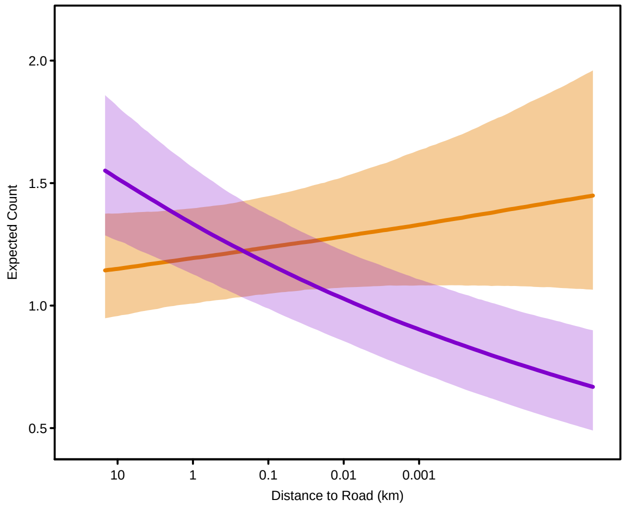

Common Yellowthroat

Geothlypis trichas

2017−19 2020

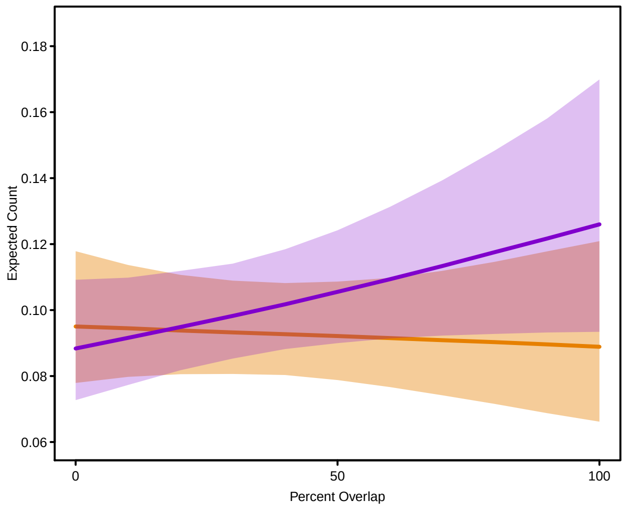

posterior distribution 95% CI
for interaction with
delta_traf includes zero

posterior distribution 95% CI
for interaction with
land includes zero

posterior distribution 95% CI
for interaction with
air includes zero

posterior distribution 95% CI
for interaction with
road includes zero

Cooper's Hawk

Accipiter cooperii

2017−19 2020

posterior distribution 95% CI
for interaction with
overlap includes zero

posterior distribution 95% CI
for interaction with
delta_traf includes zero

posterior distribution 95% CI
for interaction with
land includes zero

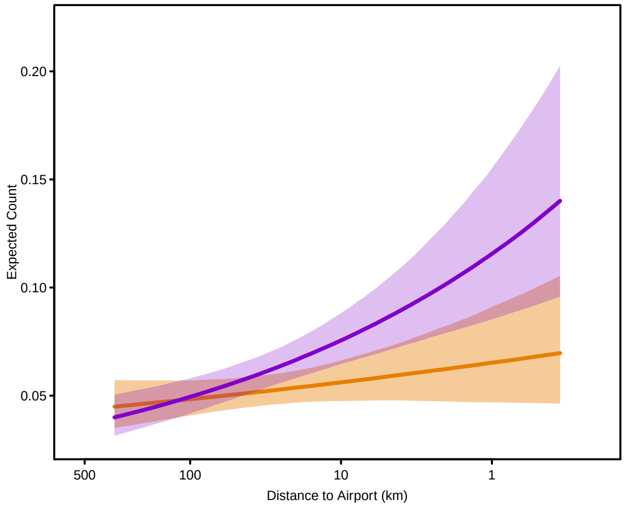

posterior distribution 95% CI
for interaction with
road includes zero

Dark−eyed Junco

Junco hyemalis

2017−19 2020

posterior distribution 95% CI
for interaction with
overlap includes zero

posterior distribution 95% CI
for interaction with
delta_traf includes zero

posterior distribution 95% CI
for interaction with
land includes zero

posterior distribution 95% CI
for interaction with
air includes zero

posterior distribution 95% CI
for interaction with
road includes zero

Double−crested Cormorant

Phalacrocorax auritus

2017−19 2020

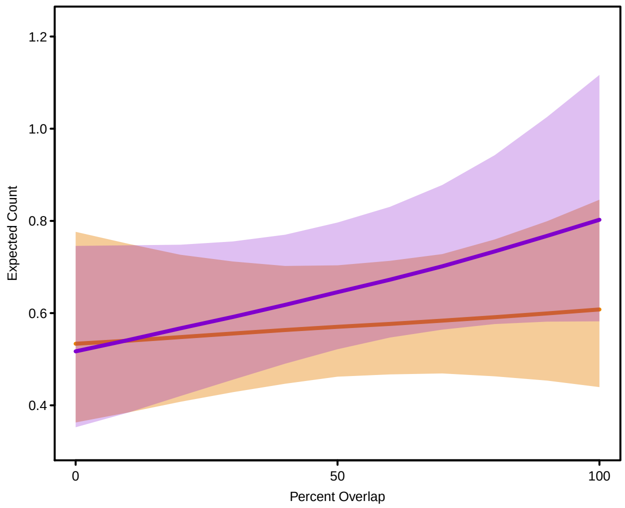

posterior distribution 95% CI
for interaction with
delta_traf includes zero

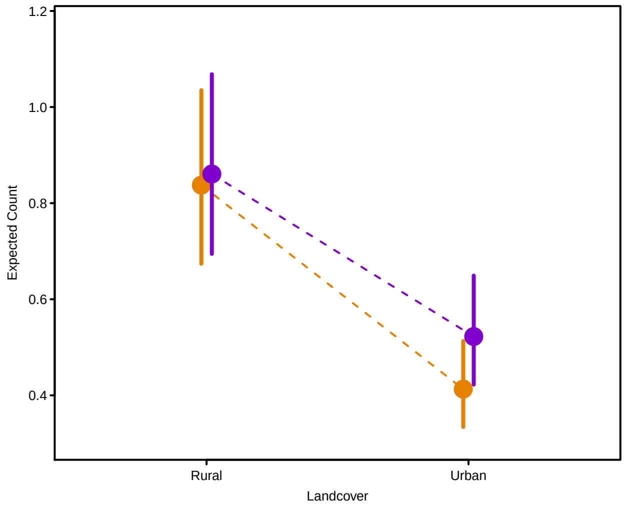

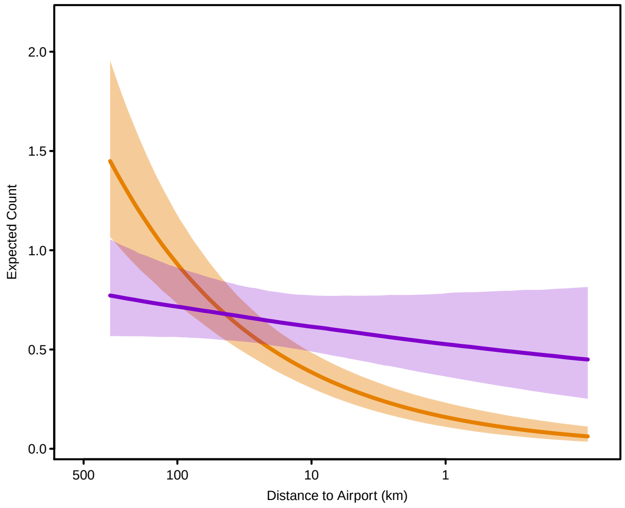

posterior distribution 95% CI
for interaction with
road includes zero

Downy Woodpecker

Dryobates pubescens

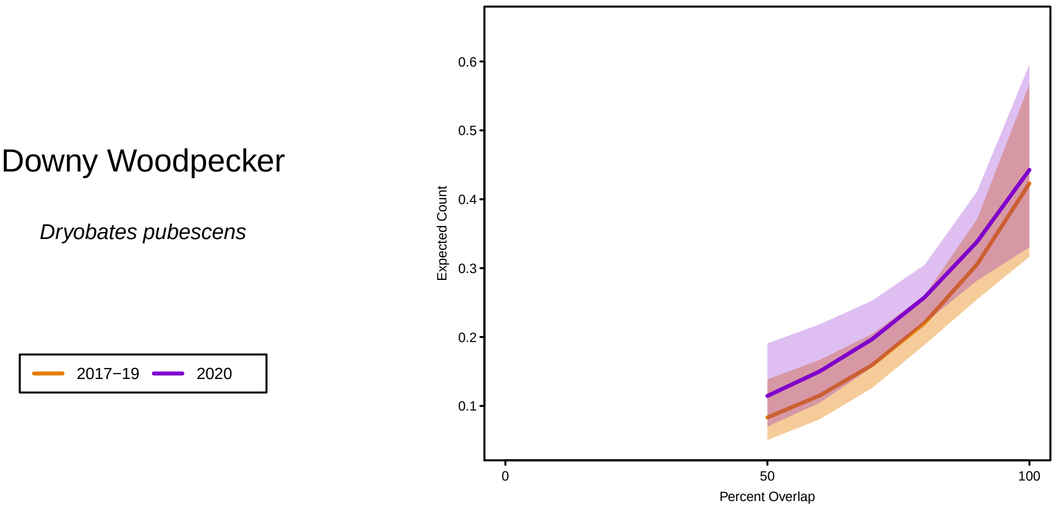

posterior distribution 95% CI
for interaction with
delta_traf includes zero

posterior distribution 95% CI
for interaction with
land includes zero

posterior distribution 95% CI
for interaction with
air includes zero

posterior distribution 95% CI
for interaction with
road includes zero

Eastern Bluebird

Sialia sialis

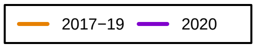

posterior distribution 95% CI
for interaction with
overlap includes zero

posterior distribution 95% CI
for interaction with
delta_traf includes zero

posterior distribution 95% CI
for interaction with
land includes zero

posterior distribution 95% CI
for interaction with
air includes zero

posterior distribution 95% CI
for interaction with
road includes zero

Eastern Kingbird

Tyrannus tyrannus

2017−19 2020

posterior distribution 95% CI
for interaction with
overlap includes zero

posterior distribution 95% CI
for interaction with
delta_traf includes zero

posterior distribution 95% CI
for interaction with
land includes zero

posterior distribution 95% CI
for interaction with
air includes zero

posterior distribution 95% CI
for interaction with
road includes zero

Eastern Phoebe

Sayornis phoebe

posterior distribution 95% CI
for interaction with
overlap includes zero

2017−19 2020

posterior distribution 95% CI
for interaction with
delta_traf includes zero

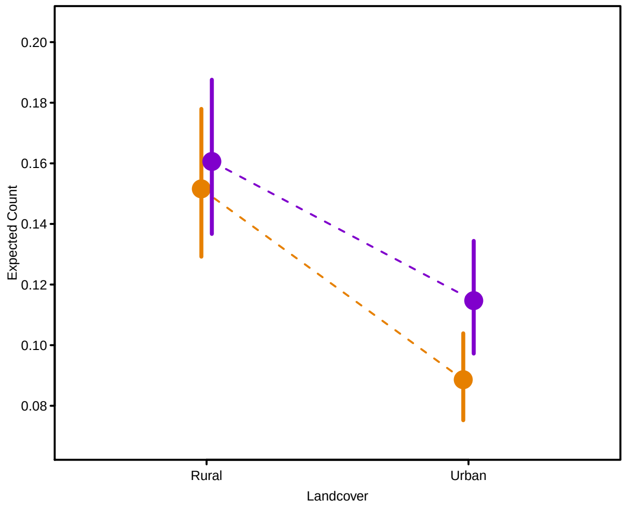

posterior distribution 95% CI
for interaction with
air includes zero

posterior distribution 95% CI
for interaction with
road includes zero

Eastern Towhee

Pipilo erythrophthalmus

posterior distribution 95% CI
for interaction with
overlap includes zero

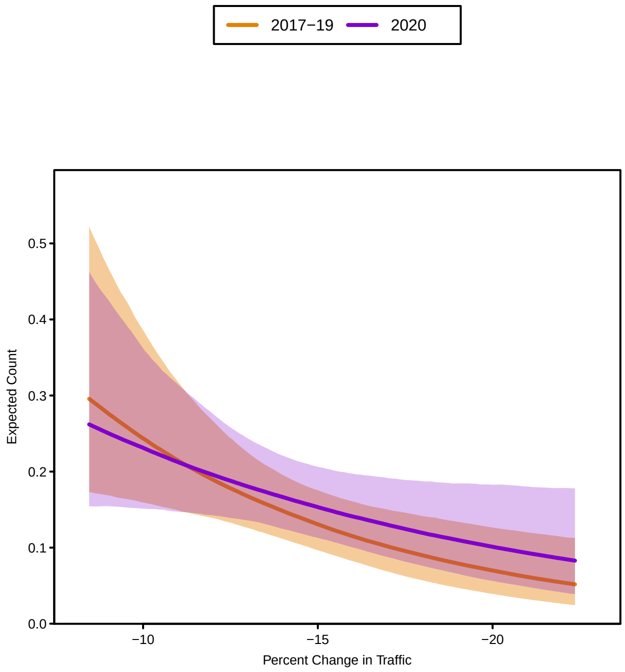

posterior distribution 95% CI
for interaction with
land includes zero

posterior distribution 95% CI
for interaction with
air includes zero

posterior distribution 95% CI
for interaction with
road includes zero

Eurasian Collared−Dove

Streptopelia decaocto

2017−19 2020

posterior distribution 95% CI
for interaction with
overlap includes zero

posterior distribution 95% CI
for interaction with
delta_traf includes zero

posterior distribution 95% CI
for interaction with
land includes zero

posterior distribution 95% CI
for interaction with
air includes zero

posterior distribution 95% CI
for interaction with
road includes zero

European Starling

Sturnus vulgaris

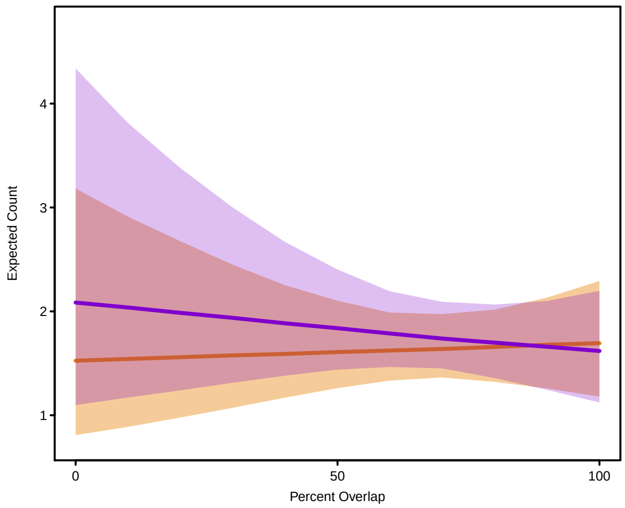

posterior distribution 95% CI
for interaction with
delta_traf includes zero

posterior distribution 95% CI
for interaction with
land includes zero

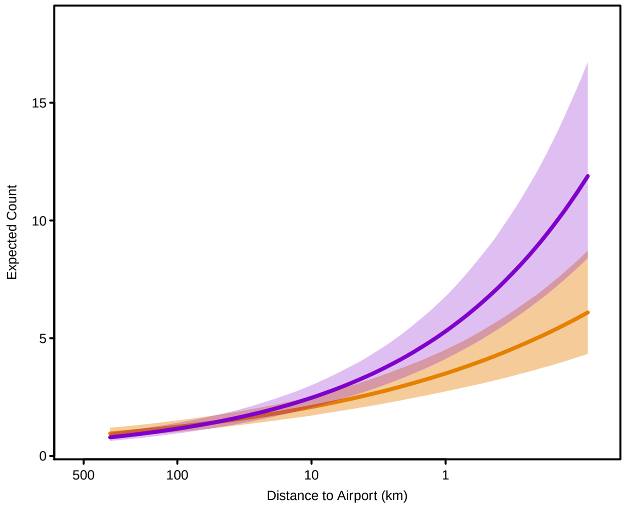

posterior distribution 95% CI
for interaction with
road includes zero

Gadwall

Mareca strepera

posterior distribution 95% CI
for interaction with
overlap includes zero

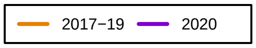

posterior distribution 95% CI
for interaction with
delta_traf includes zero

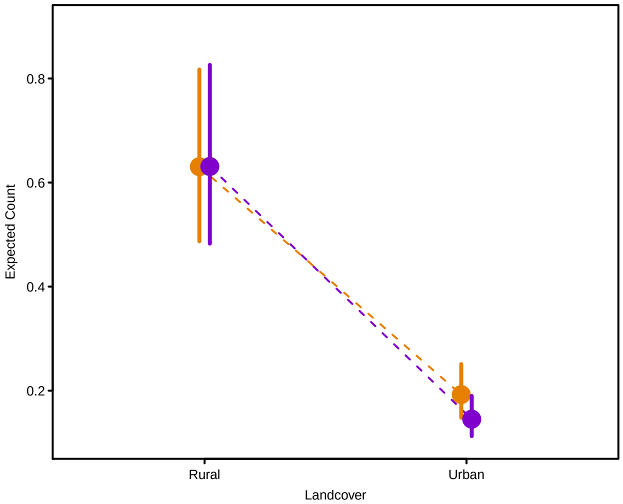

posterior distribution 95% CI
for interaction with
air includes zero

posterior distribution 95% CI
for interaction with
road includes zero

Gray Catbird

Dumetella carolinensis

2017−19 2020

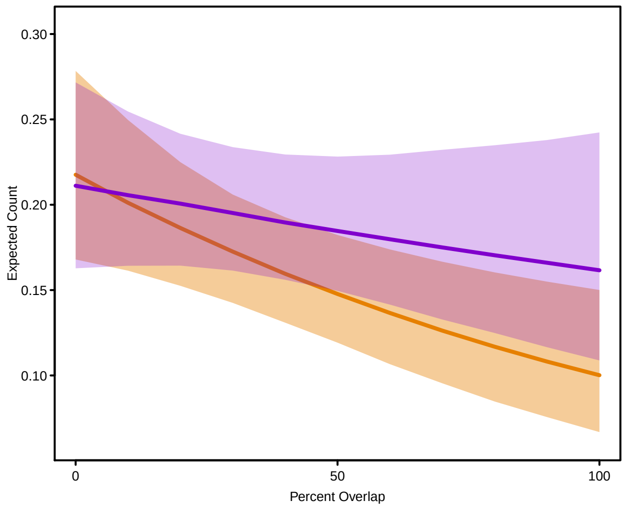

posterior distribution 95% CI
for interaction with
delta_traf includes zero

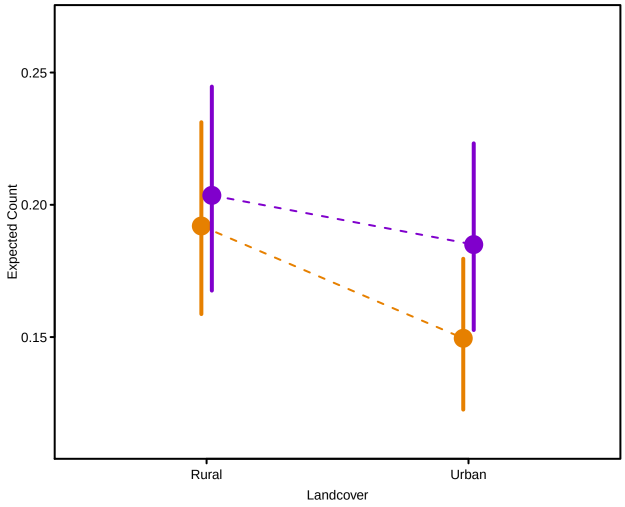

posterior distribution 95% CI
for interaction with
air includes zero

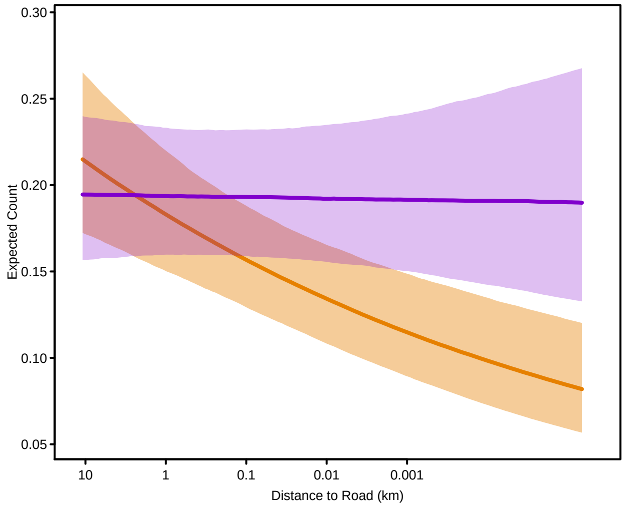

Great Blue Heron

Ardea herodias

2017−19 2020

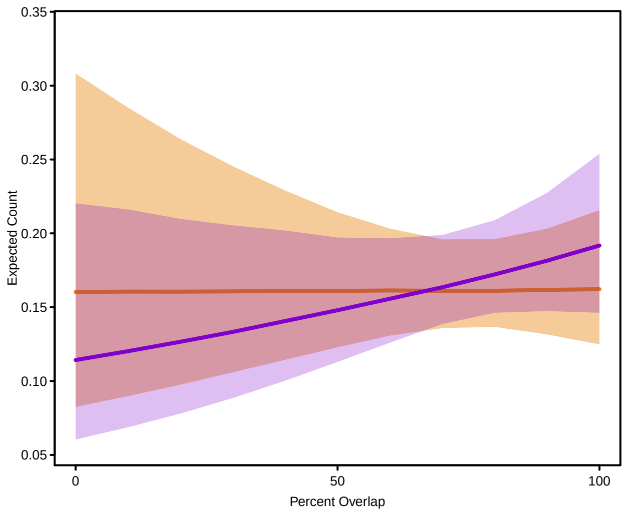

posterior distribution 95% CI
for interaction with
delta_traf includes zero

posterior distribution 95% CI
for interaction with
land includes zero

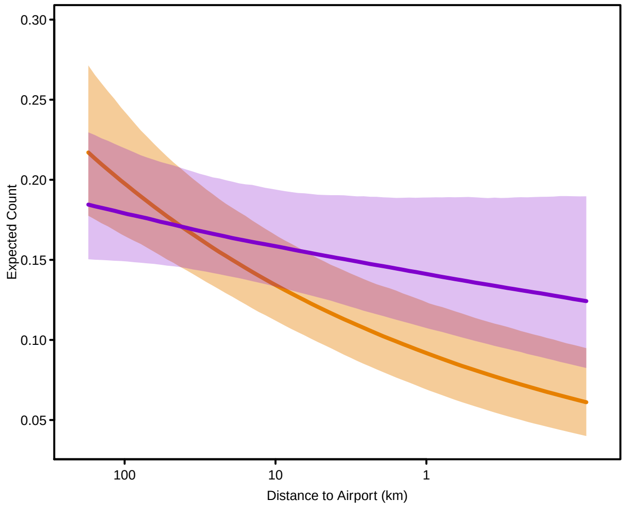

posterior distribution 95% CI
for interaction with
road includes zero

Great Crested Flycatcher

Myiarchus crinitus

2017−19 2020

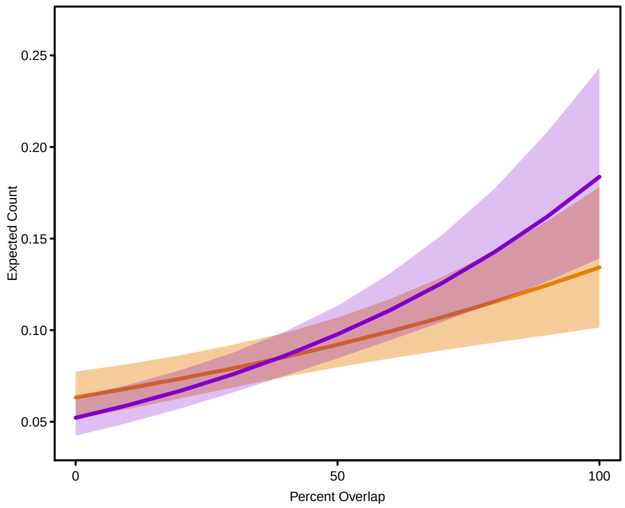

posterior distribution 95% CI
for interaction with
delta_traf includes zero

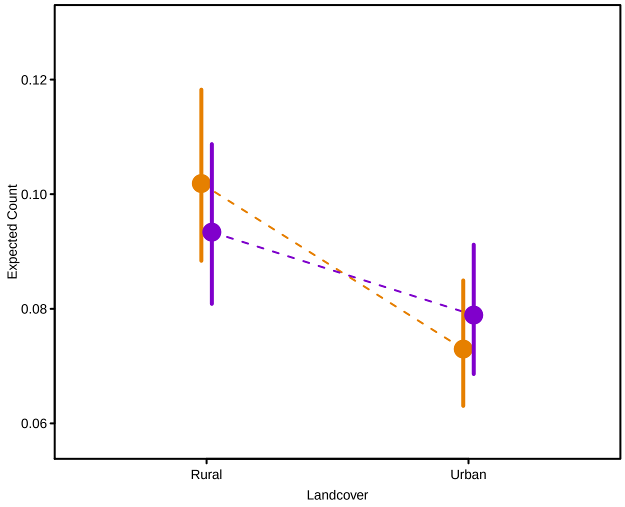

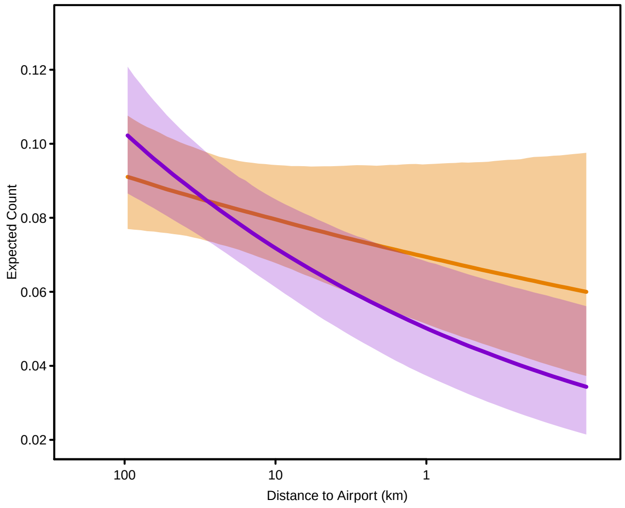

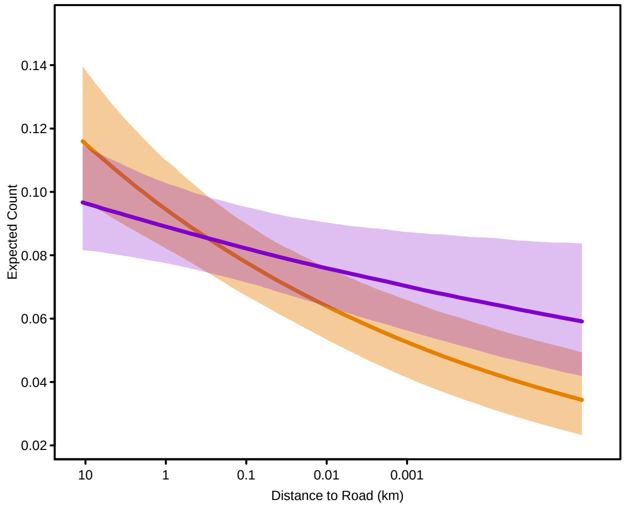

Great Egret

Ardea alba

posterior distribution 95% CI
for interaction with
overlap includes zero

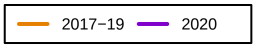

posterior distribution 95% CI
for interaction with
delta_traf includes zero

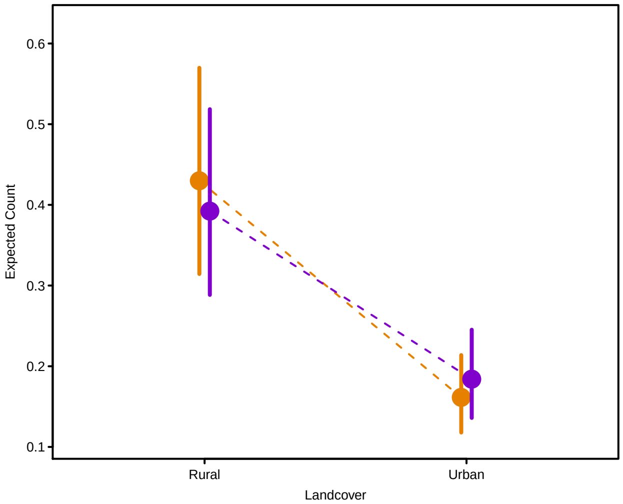

posterior distribution 95% CI
for interaction with
air includes zero

posterior distribution 95% CI
for interaction with
road includes zero

Hairy Woodpecker

Dryobates villosus

posterior distribution 95% CI
for interaction with
overlap includes zero

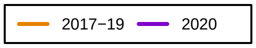

posterior distribution 95% CI
for interaction with
delta_traf includes zero

posterior distribution 95% CI
for interaction with
land includes zero

posterior distribution 95% CI
for interaction with
air includes zero

posterior distribution 95% CI
for interaction with
road includes zero

House Finch

Haemorhous mexicanus

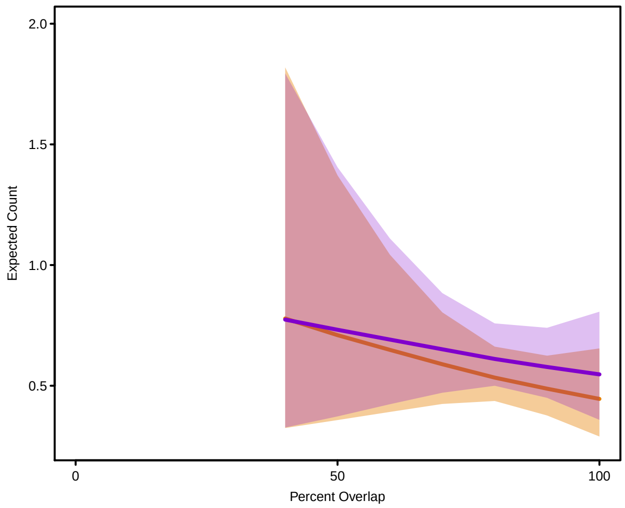

posterior distribution 95% CI
for interaction with
delta_traf includes zero

posterior distribution 95% CI
for interaction with
land includes zero

posterior distribution 95% CI
for interaction with
air includes zero

posterior distribution 95% CI
for interaction with
road includes zero

House Sparrow

Passer domesticus

2017−19 2020

posterior distribution 95% CI
for interaction with
overlap includes zero

posterior distribution 95% CI
for interaction with
delta_traf includes zero

posterior distribution 95% CI
for interaction with
land includes zero

posterior distribution 95% CI
for interaction with
air includes zero

posterior distribution 95% CI
for interaction with
road includes zero

House Wren

Troglodytes aedon

2017−19 2020

posterior distribution 95% CI
for interaction with
overlap includes zero

posterior distribution 95% CI
for interaction with
delta_traf includes zero

posterior distribution 95% CI
for interaction with
land includes zero

posterior distribution 95% CI
for interaction with
air includes zero

posterior distribution 95% CI
for interaction with
road includes zero

Indigo Bunting

Passerina cyanea

2017−19 2020

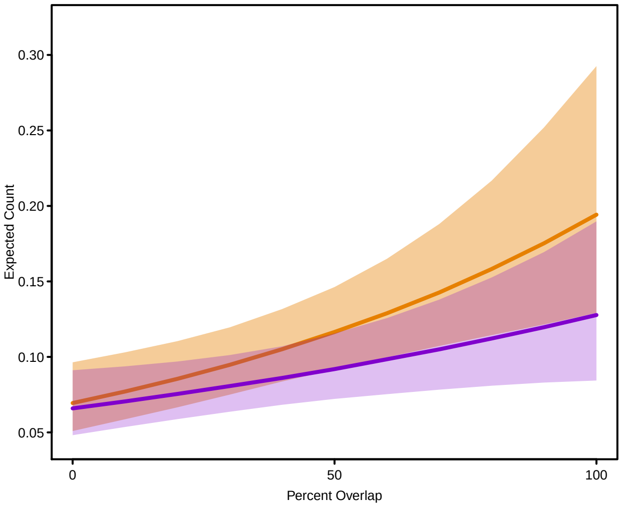

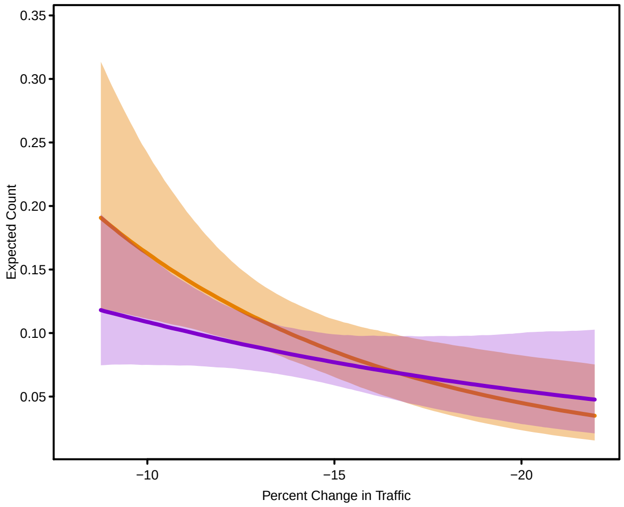

posterior distribution 95% CI
for interaction with
land includes zero

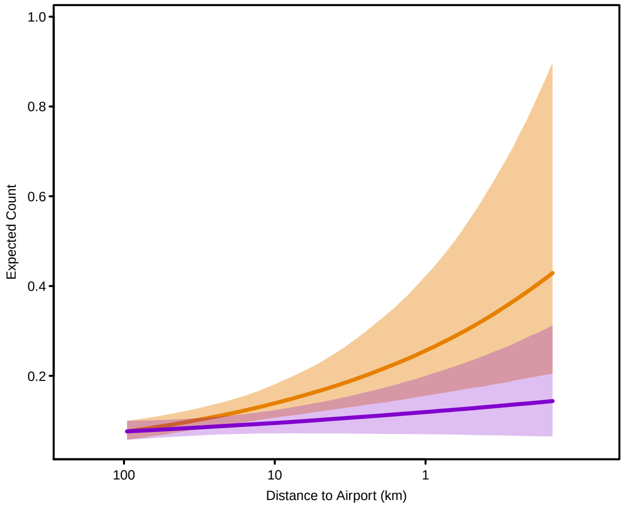

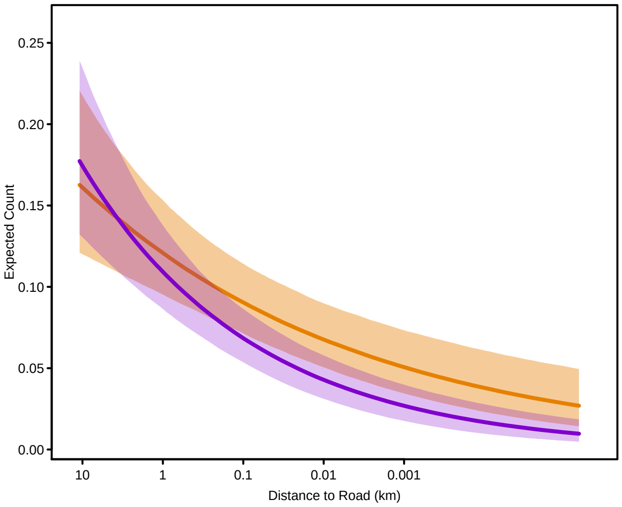

Killdeer

Charadrius vociferus

2017−19 2020

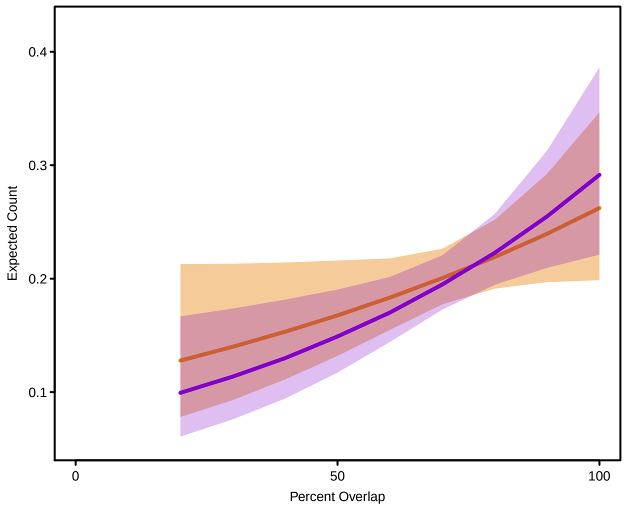

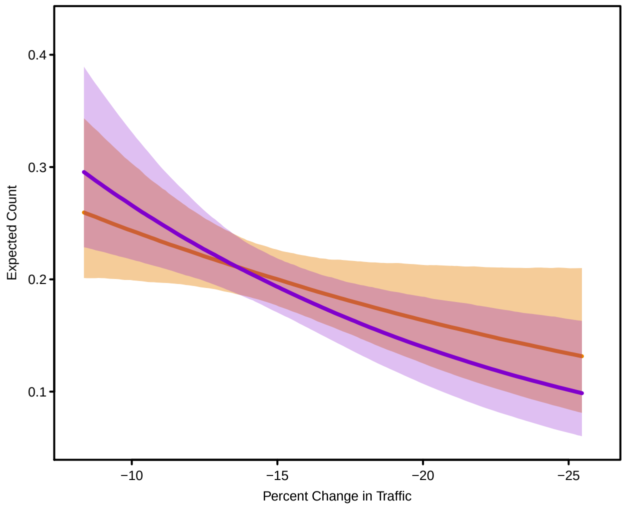

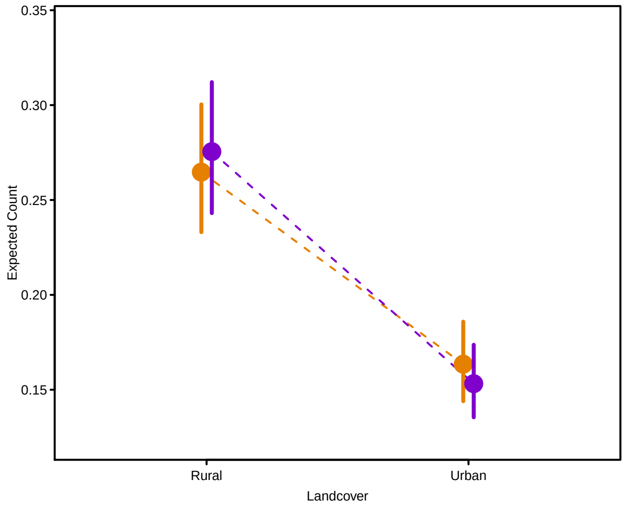

posterior distribution 95% CI
for interaction with
air includes zero

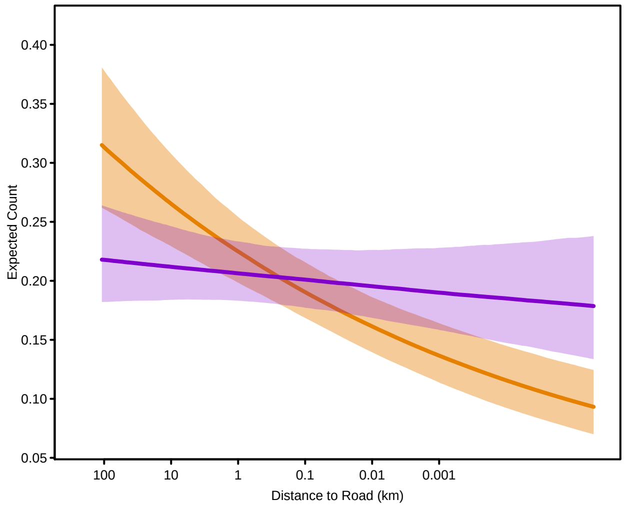

Mallard

Anas platyrhynchos

2017−19 2020

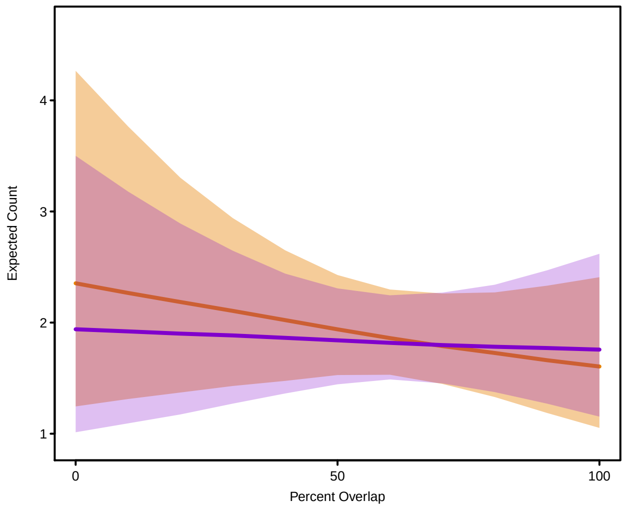

posterior distribution 95% CI
for interaction with
delta_traf includes zero

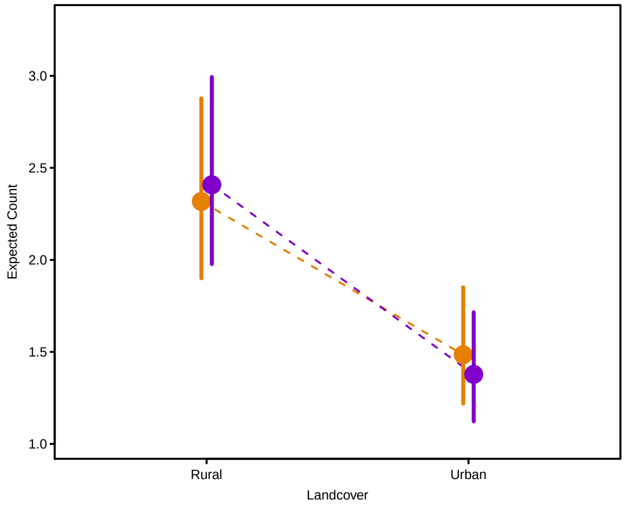

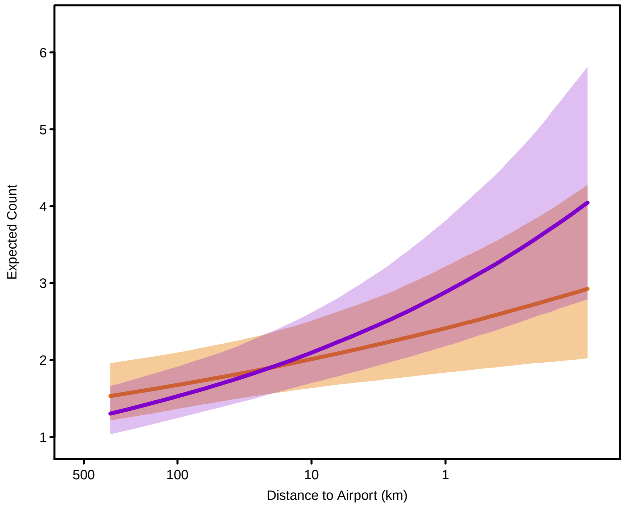

posterior distribution 95% CI
for interaction with
road includes zero

Mourning Dove

Zenaida macroura

2017−19 2020

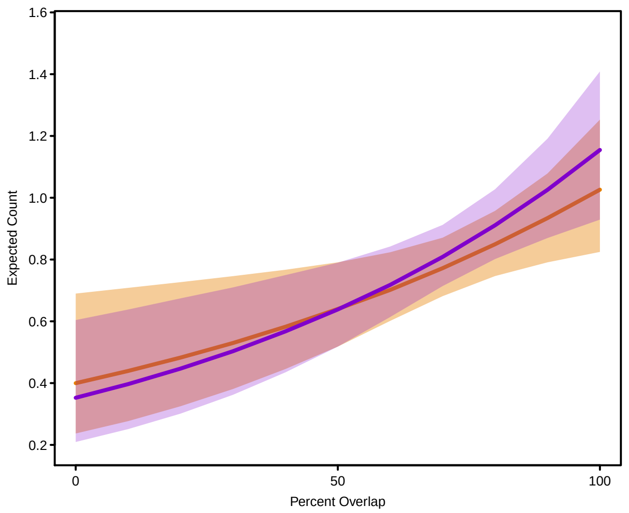

posterior distribution 95% CI
for interaction with
delta_traf includes zero

posterior distribution 95% CI
for interaction with
land includes zero

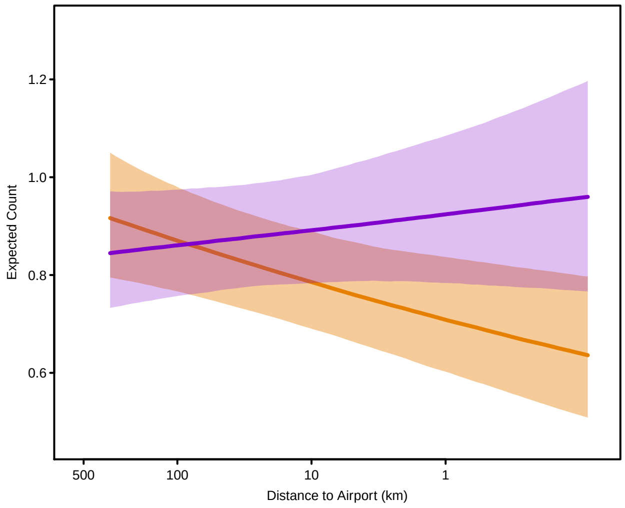

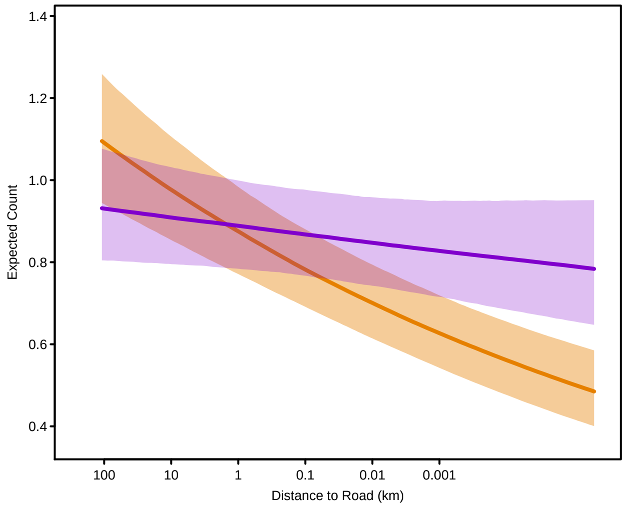

Nashville Warbler

Leiothlypis ruficapilla

2017−19 2020

posterior distribution 95% CI
for interaction with
overlap includes zero

posterior distribution 95% CI
for interaction with
delta_traf includes zero

posterior distribution 95% CI
for interaction with
land includes zero

posterior distribution 95% CI
for interaction with
air includes zero

posterior distribution 95% CI
for interaction with
road includes zero

Northern Cardinal

Cardinalis cardinalis

posterior distribution 95% CI
for interaction with
overlap includes zero

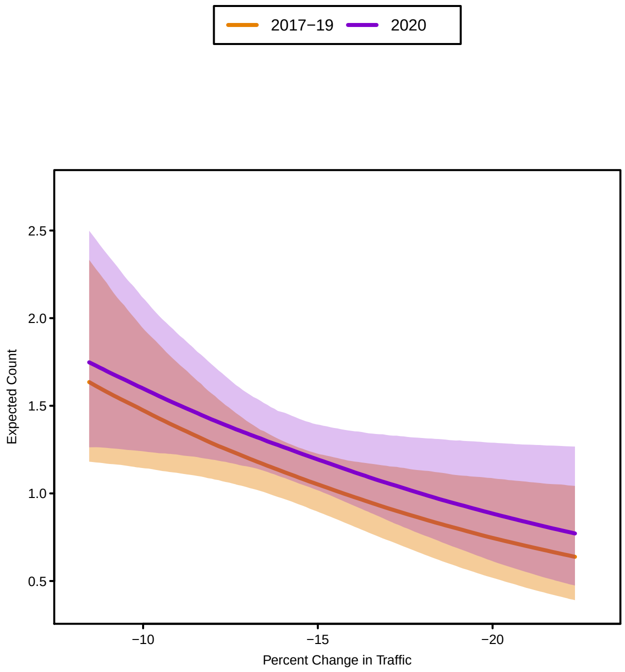

posterior distribution 95% CI
for interaction with
land includes zero

posterior distribution 95% CI
for interaction with
air includes zero

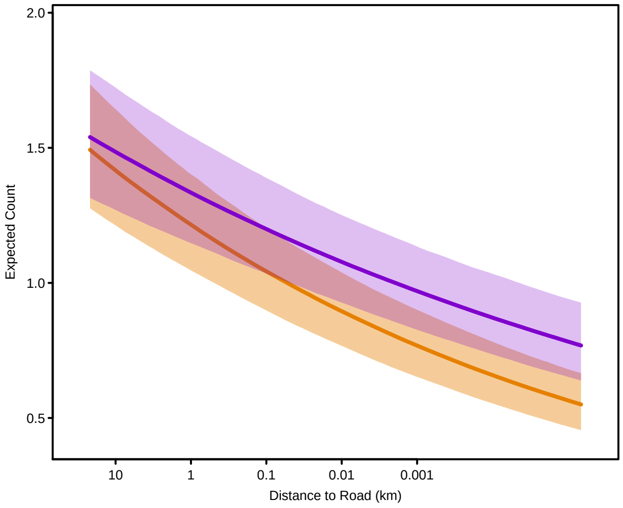

Northern Flicker

Colaptes auratus

2017−19 2020

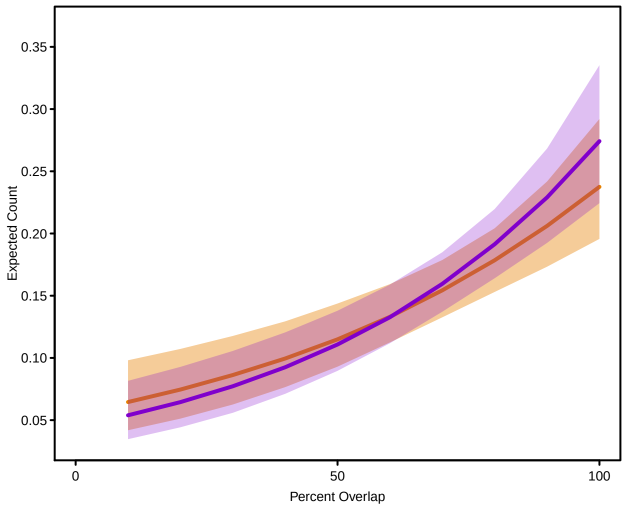

posterior distribution 95% CI
for interaction with
delta_traf includes zero

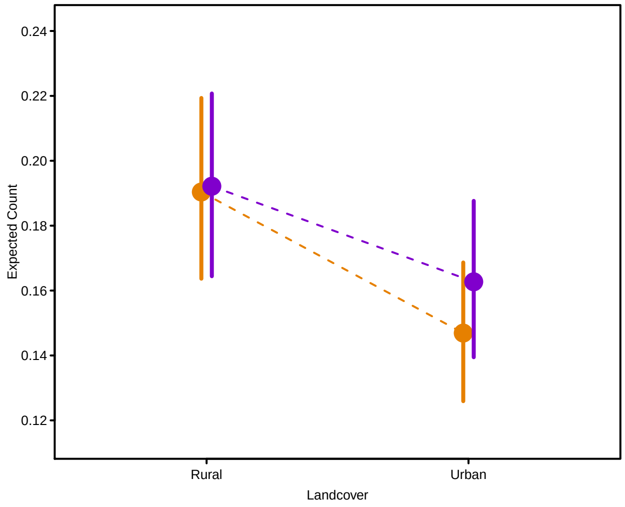

posterior distribution 95% CI
for interaction with
air includes zero

posterior distribution 95% CI
for interaction with
road includes zero

Northern Mockingbird

Mimus polyglottos

2017−19 2020

posterior distribution 95% CI
for interaction with
overlap includes zero

posterior distribution 95% CI
for interaction with
delta_traf includes zero

posterior distribution 95% CI
for interaction with
land includes zero

posterior distribution 95% CI
for interaction with
air includes zero

posterior distribution 95% CI
for interaction with
road includes zero

Northern Parula

Setophaga americana

2017−19 2020

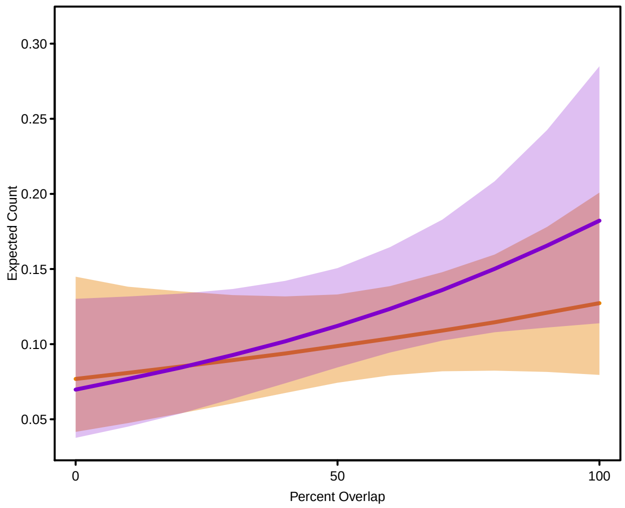

posterior distribution 95% CI
for interaction with
delta_traf includes zero

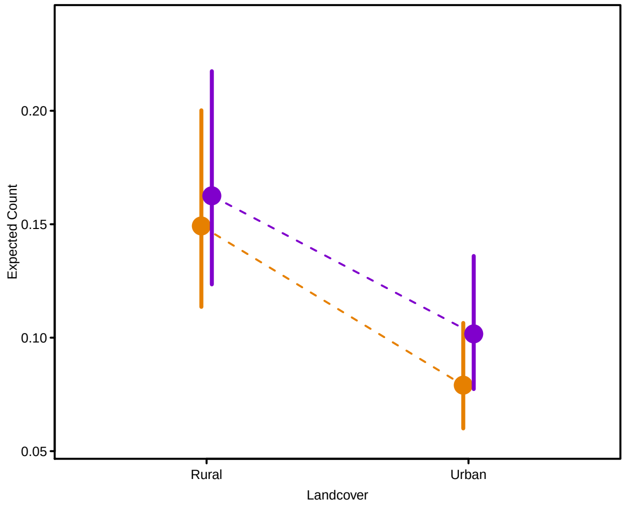

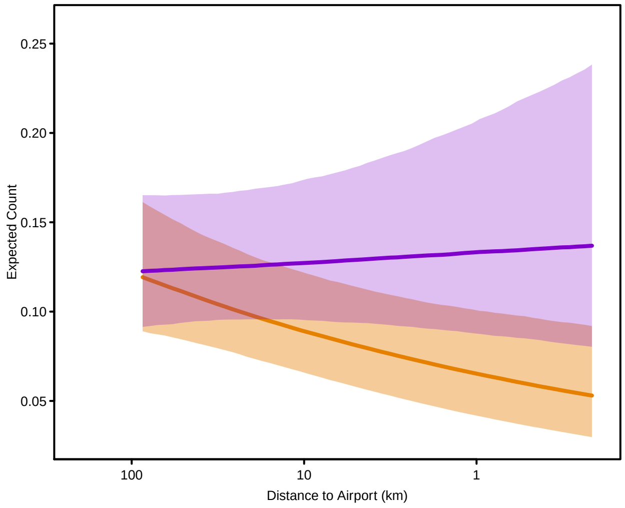

posterior distribution 95% CI
for interaction with
road includes zero

Northern Rough−winged Swallow

Stelgidopteryx serripennis

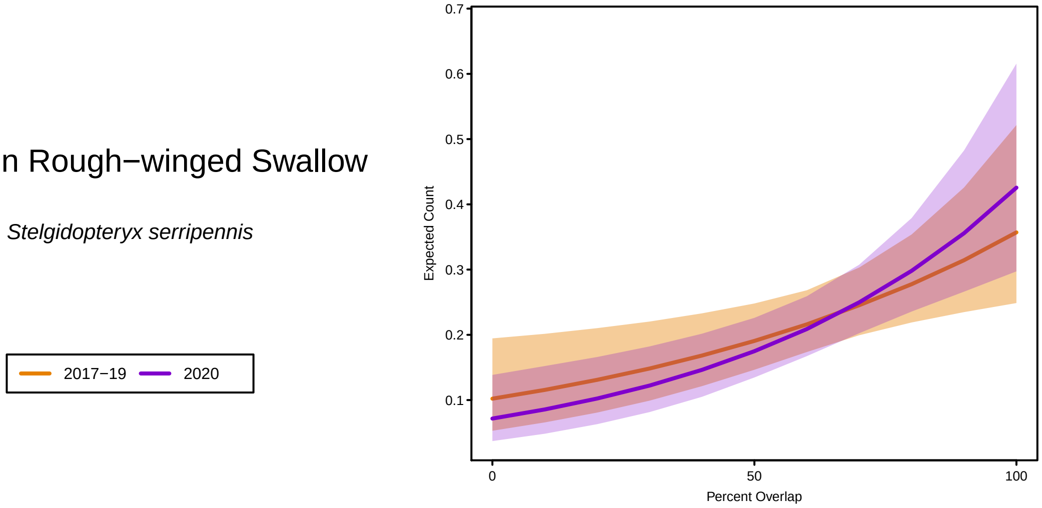

posterior distribution 95% CI
for interaction with
delta_traf includes zero

posterior distribution 95% CI
for interaction with
land includes zero

posterior distribution 95% CI
for interaction with
air includes zero

posterior distribution 95% CI
for interaction with
road includes zero

Orange−crowned Warbler

Leiothlypis celata

2017−19 2020

posterior distribution 95% CI
for interaction with
overlap includes zero

posterior distribution 95% CI
for interaction with
delta_traf includes zero

posterior distribution 95% CI
for interaction with
land includes zero

posterior distribution 95% CI
for interaction with
air includes zero

posterior distribution 95% CI
for interaction with
road includes zero

Osprey

Pandion haliaetus

posterior distribution 95% CI
for interaction with
overlap includes zero

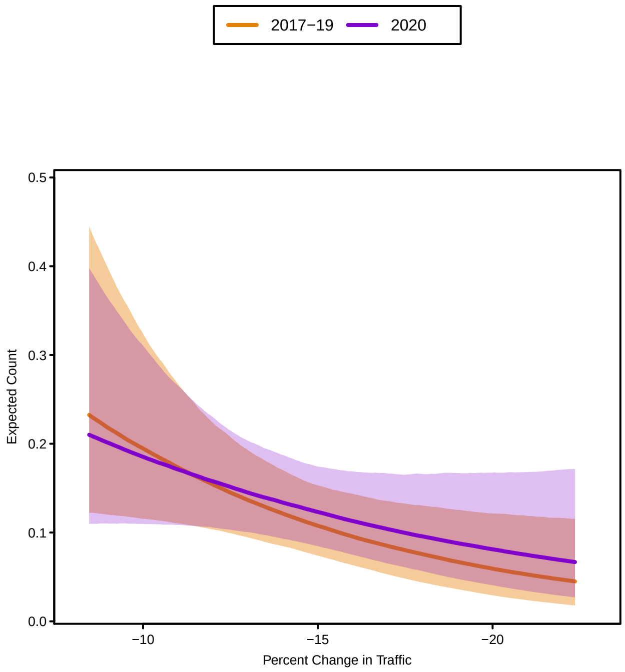

posterior distribution 95% CI
for interaction with
land includes zero

posterior distribution 95% CI
for interaction with
air includes zero

posterior distribution 95% CI
for interaction with
road includes zero

Palm Warbler

Setophaga palmarum

2017−19 2020

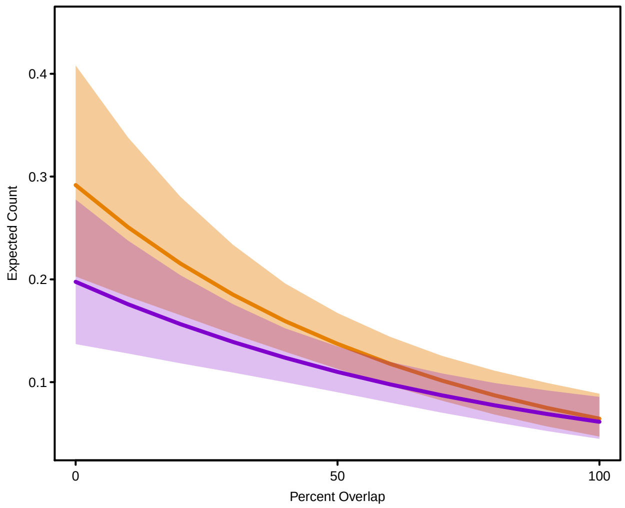

posterior distribution 95% CI
for interaction with
delta_traf includes zero

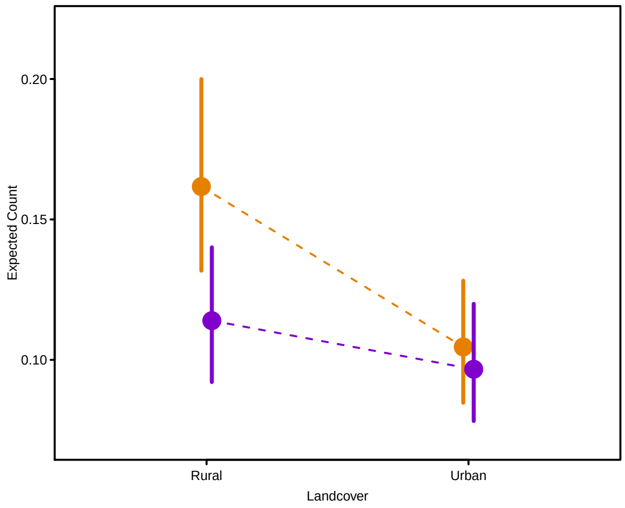

posterior distribution 95% CI
for interaction with
air includes zero

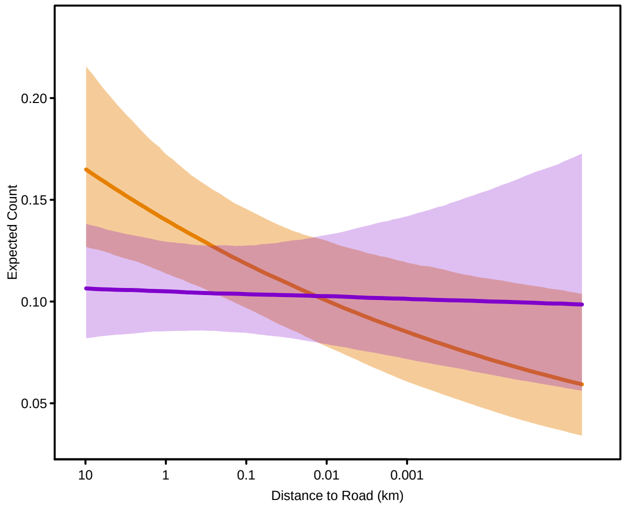

Pied−billed Grebe

Podilymbus podiceps

2017−19 2020

posterior distribution 95% CI
for interaction with
overlap includes zero

posterior distribution 95% CI
for interaction with
delta_traf includes zero

posterior distribution 95% CI
for interaction with
land includes zero

posterior distribution 95% CI
for interaction with
air includes zero

posterior distribution 95% CI
for interaction with
road includes zero

Pileated Woodpecker

Dryocopus pileatus

posterior distribution 95% CI
for interaction with
overlap includes zero

2017−19 2020

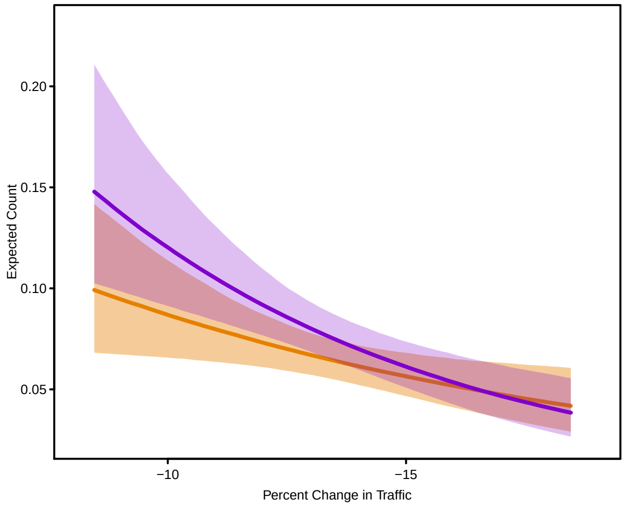

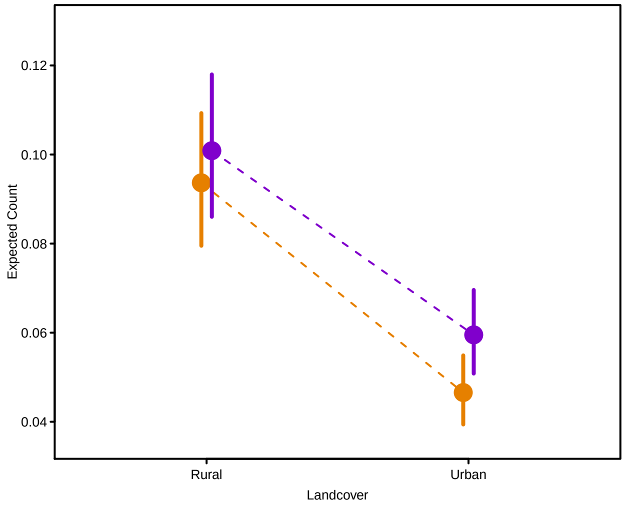

posterior distribution 95% CI
for interaction with
air includes zero

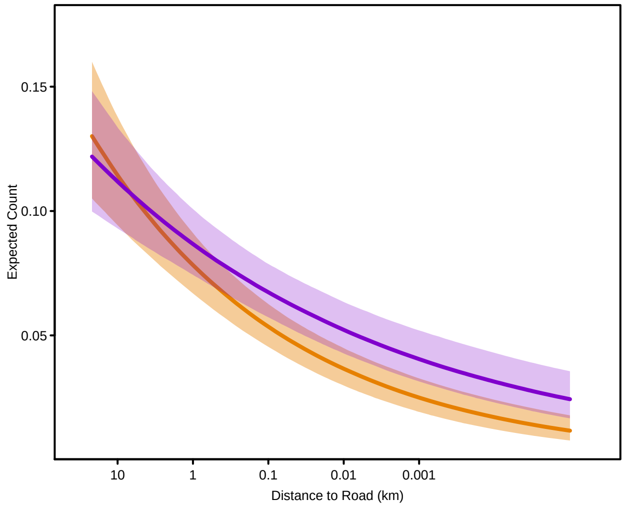

Red−bellied Woodpecker

Melanerpes carolinus

posterior distribution 95% CI
for interaction with
overlap includes zero

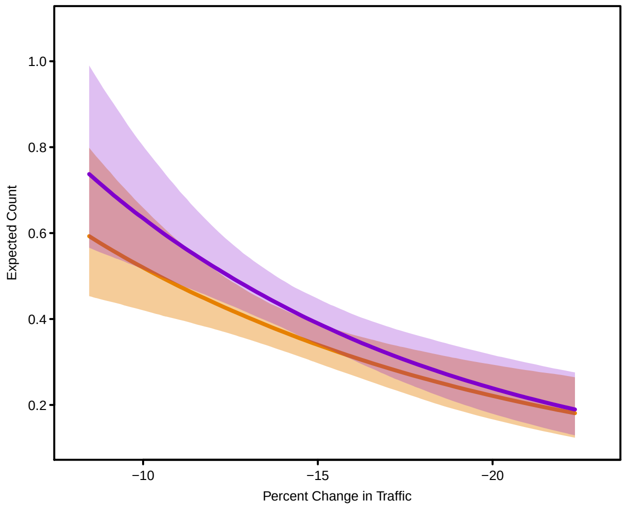

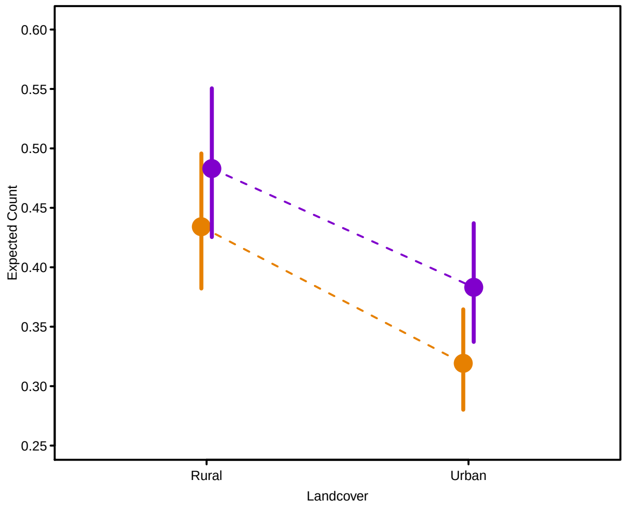

posterior distribution 95% CI
for interaction with
air includes zero

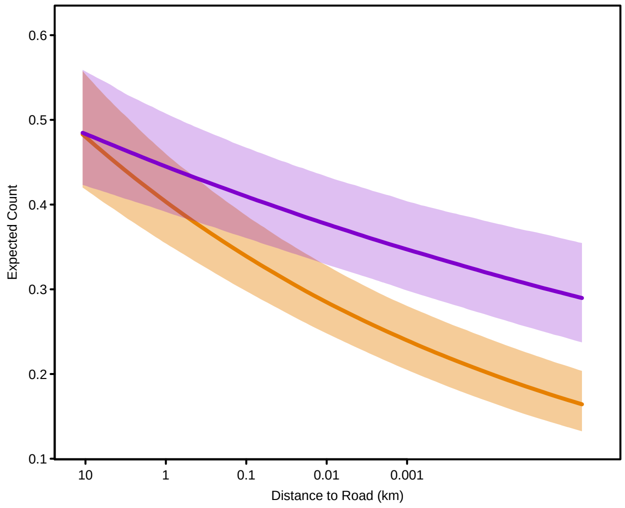

Red−eyed Vireo

Vireo olivaceus

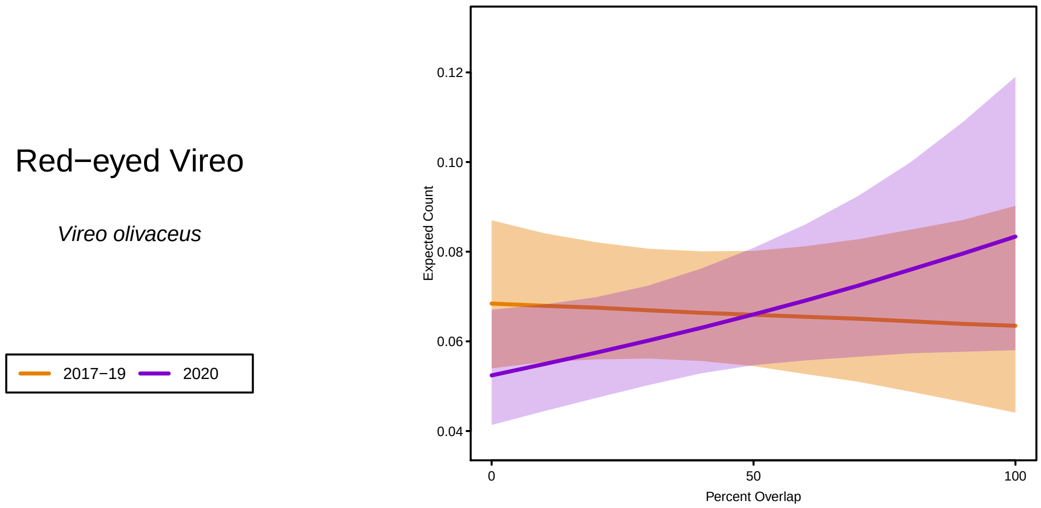

posterior distribution 95% CI
for interaction with
delta_traf includes zero

posterior distribution 95% CI
for interaction with
land includes zero

posterior distribution 95% CI
for interaction with
air includes zero

posterior distribution 95% CI
for interaction with
road includes zero

Red−shouldered Hawk

Buteo lineatus

posterior distribution 95% CI
for interaction with
overlap includes zero

2017−19 2020

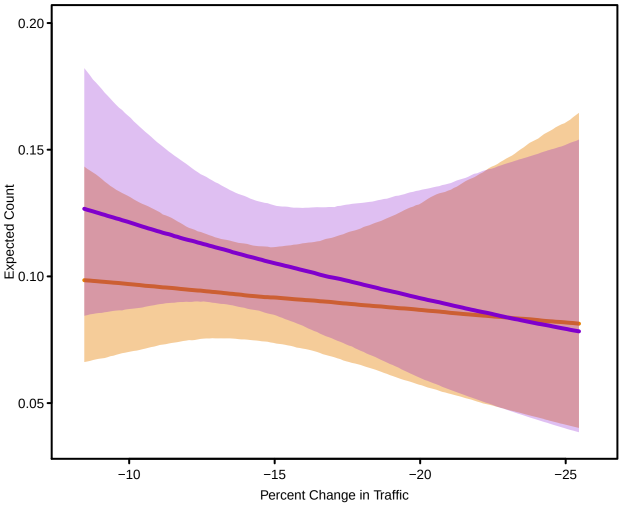

posterior distribution 95% CI
for interaction with
land includes zero

posterior distribution 95% CI
for interaction with
air includes zero

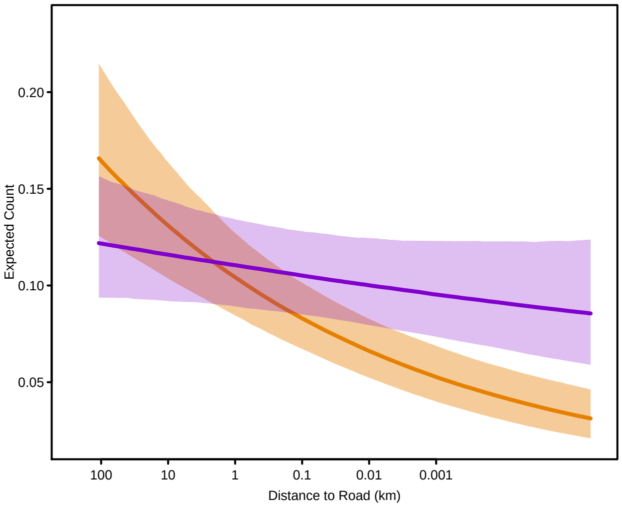

Red−tailed Hawk

Buteo jamaicensis

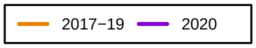

posterior distribution 95% CI
for interaction with
overlap includes zero

posterior distribution 95% CI
for interaction with
delta_traf includes zero

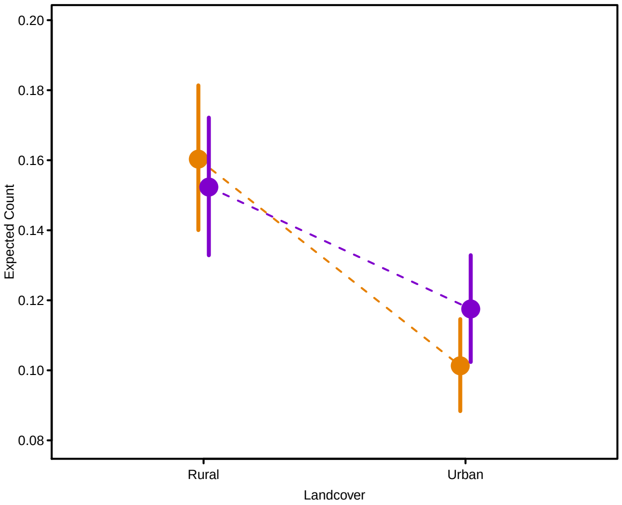

posterior distribution 95% CI
for interaction with
air includes zero

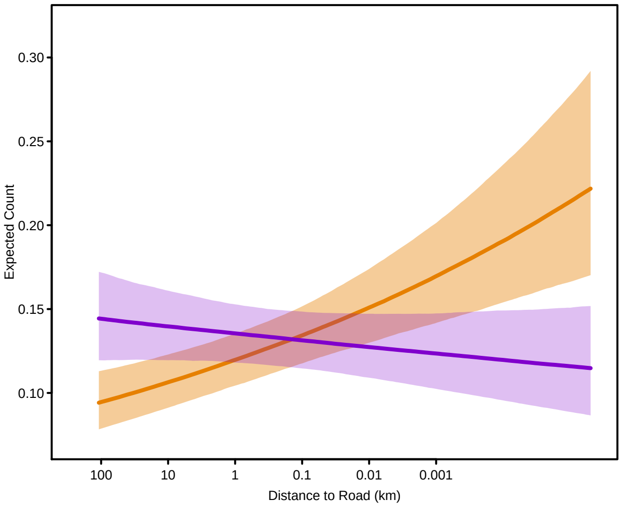

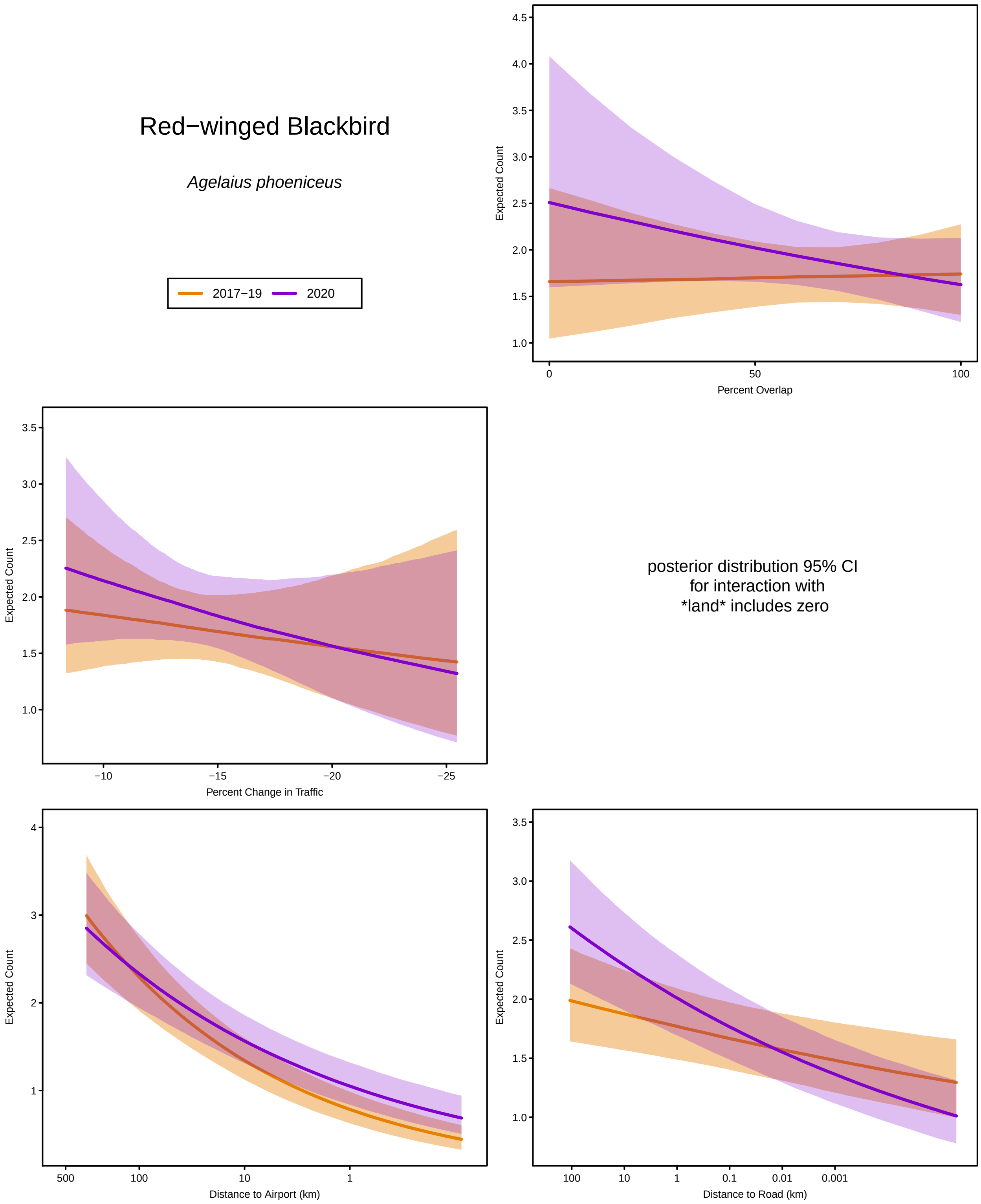
Red−winged Blackbird
Agelaius phoeniceus
2017−19
2020
Expected Count
Percent Overlap
Percent Change in Traffic
posterior distribution 95% CI
for interaction with
land includes zero
Distance to Airport (km)
Distance to Road (km)

Ring−billed Gull

Larus delawarensis

posterior distribution 95% CI
for interaction with
overlap includes zero

2017−19 2020

posterior distribution 95% CI
for interaction with
delta_traf includes zero

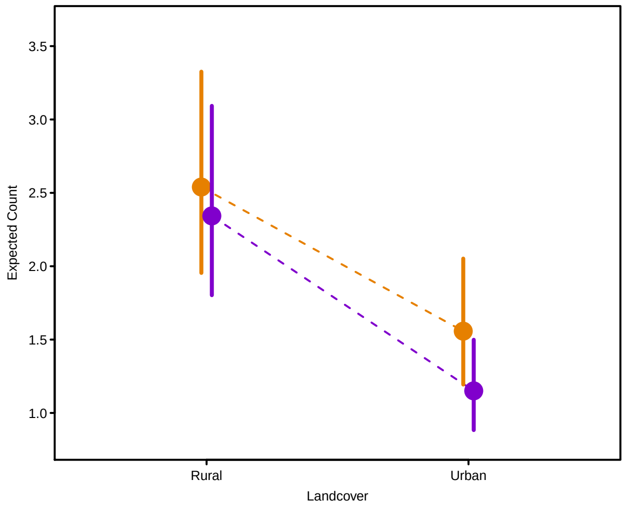

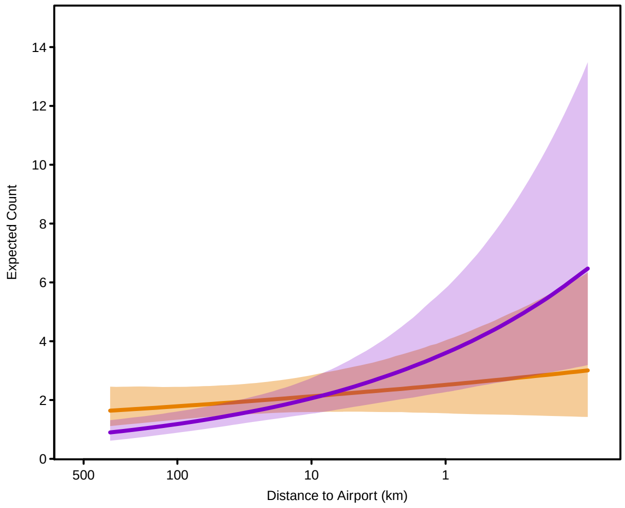

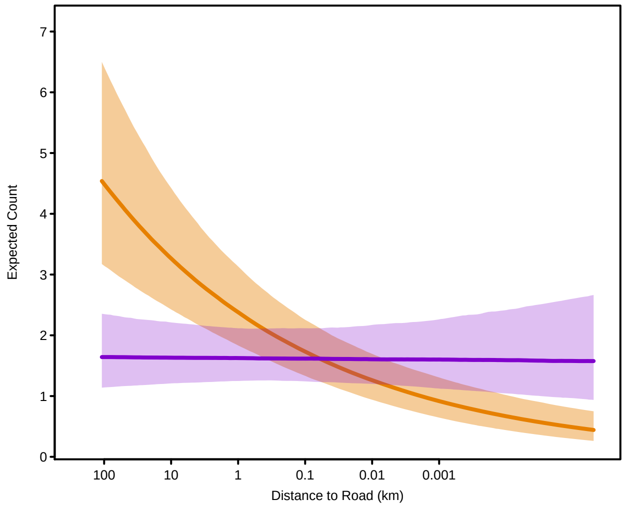

Rock Pigeon

Columba livia

2017−19 2020

posterior distribution 95% CI
for interaction with
overlap includes zero

posterior distribution 95% CI
for interaction with
delta_traf includes zero

posterior distribution 95% CI
for interaction with
land includes zero

posterior distribution 95% CI
for interaction with
air includes zero

posterior distribution 95% CI
for interaction with
road includes zero

Rose−breasted Grosbeak

Pheucticus ludovicianus

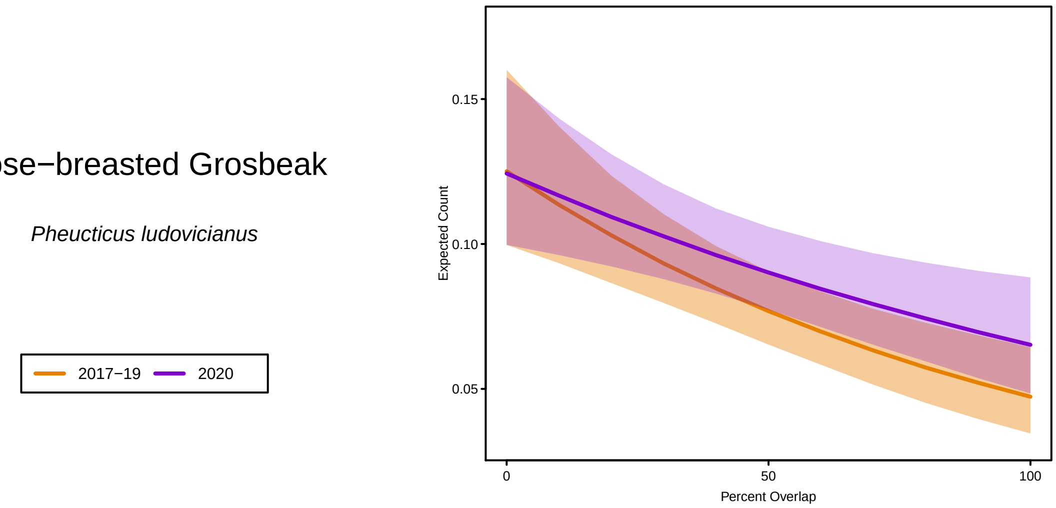

posterior distribution 95% CI
for interaction with
delta_traf includes zero

posterior distribution 95% CI
for interaction with
land includes zero

posterior distribution 95% CI
for interaction with
air includes zero

posterior distribution 95% CI
for interaction with
road includes zero

Ruby−crowned Kinglet

Regulus calendula

posterior distribution 95% CI
for interaction with
overlap includes zero

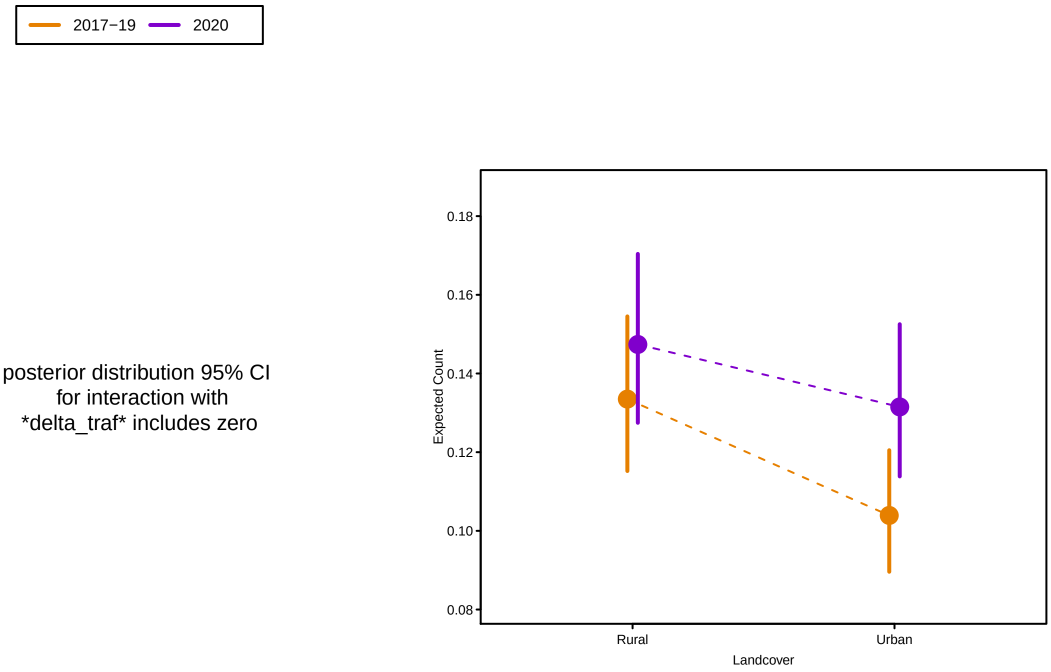

posterior distribution 95% CI
for interaction with
delta_traf includes zero

posterior distribution 95% CI
for interaction with
air includes zero

posterior distribution 95% CI
for interaction with
road includes zero

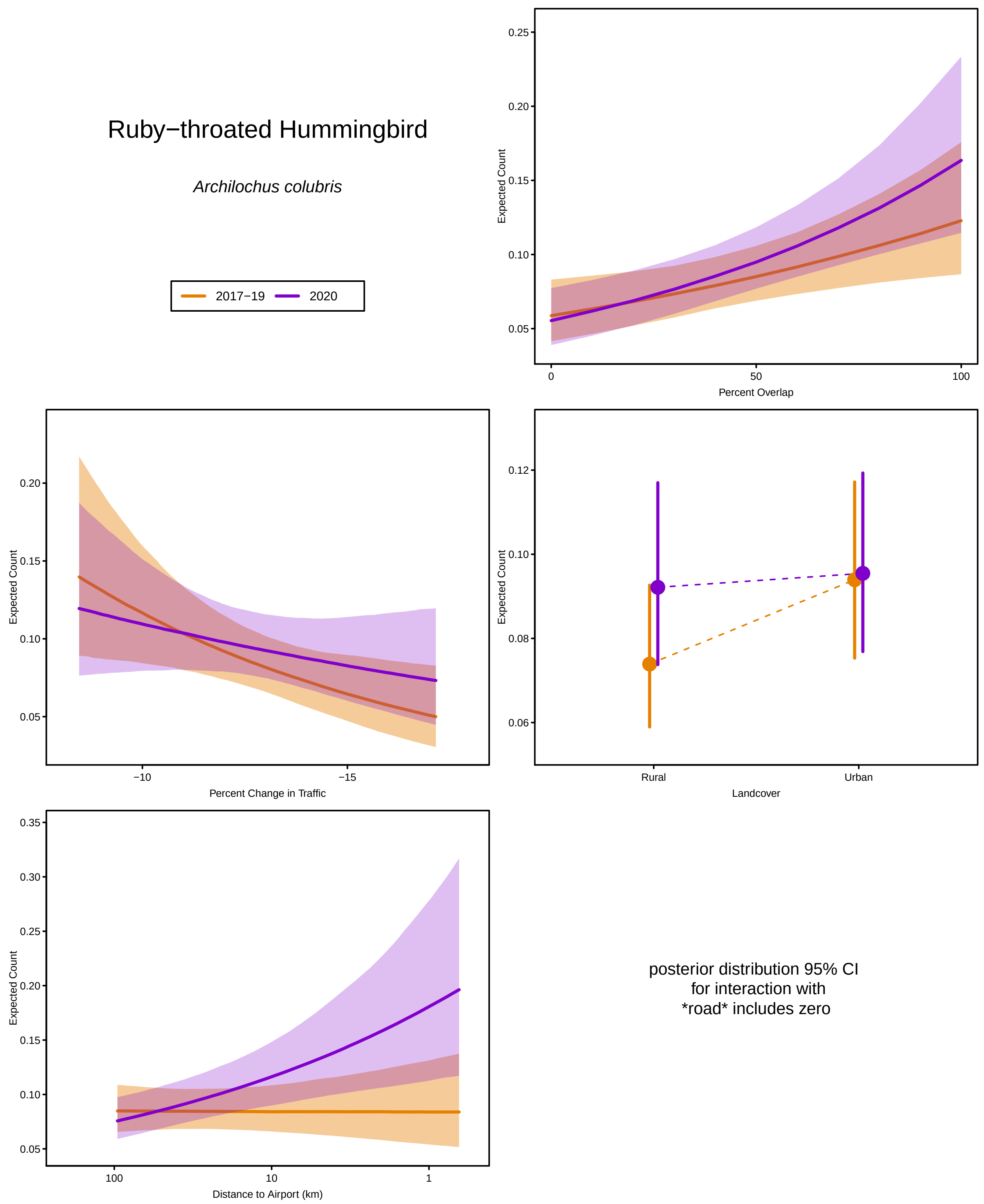
Ruby−throated Hummingbird
Archilochus colubris
2017−19
2020
Expected Count
0.25
0.20
0.15
0.10
0.05
0
50
100
Percent Overlap
0.20
0.15
0.10
0.05
−10
−15
Percent Change in Traffic
0.12
0.10
0.08
0.06
Rural
Urban
Landcover
0.35
0.30
0.25
0.20
0.15
0.10
0.05
100
10
1
Distance to Airport (km)
posterior distribution 95% CI
for interaction with
road includes zero

Snowy Egret

Egretta thula

2017−19 2020

posterior distribution 95% CI
for interaction with
overlap includes zero

posterior distribution 95% CI
for interaction with
delta_traf includes zero

posterior distribution 95% CI
for interaction with
land includes zero

posterior distribution 95% CI
for interaction with
air includes zero

posterior distribution 95% CI
for interaction with
road includes zero

Song Sparrow

Melospiza melodia

2017−19 2020

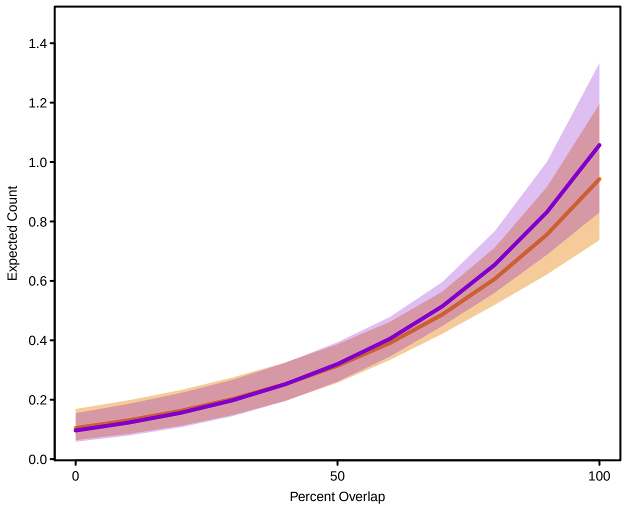

posterior distribution 95% CI
for interaction with
delta_traf includes zero

posterior distribution 95% CI
for interaction with
land includes zero

posterior distribution 95% CI
for interaction with
air includes zero

posterior distribution 95% CI
for interaction with
road includes zero

Swainson's Thrush

Catharus ustulatus

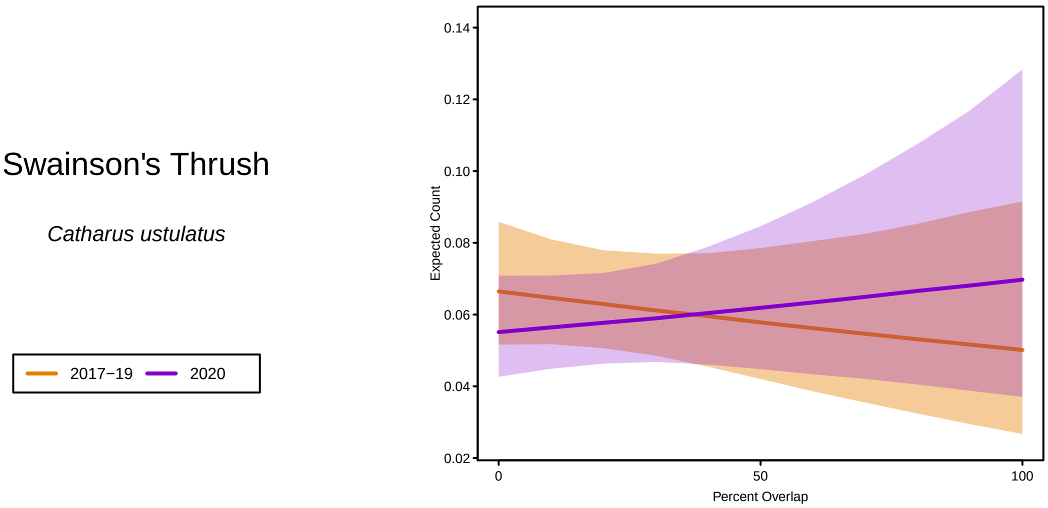

posterior distribution 95% CI
for interaction with
delta_traf includes zero

posterior distribution 95% CI
for interaction with
land includes zero

posterior distribution 95% CI
for interaction with
air includes zero

posterior distribution 95% CI
for interaction with
road includes zero

Tree Swallow

Tachycineta bicolor

posterior distribution 95% CI
for interaction with
overlap includes zero

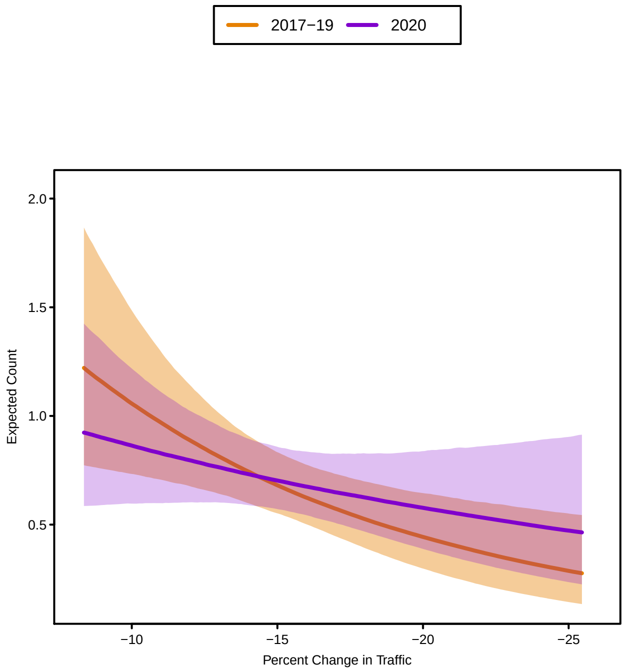

posterior distribution 95% CI
for interaction with
land includes zero

posterior distribution 95% CI
for interaction with
air includes zero

posterior distribution 95% CI
for interaction with
road includes zero

Tufted Titmouse

Baeolophus bicolor

2017−19 2020

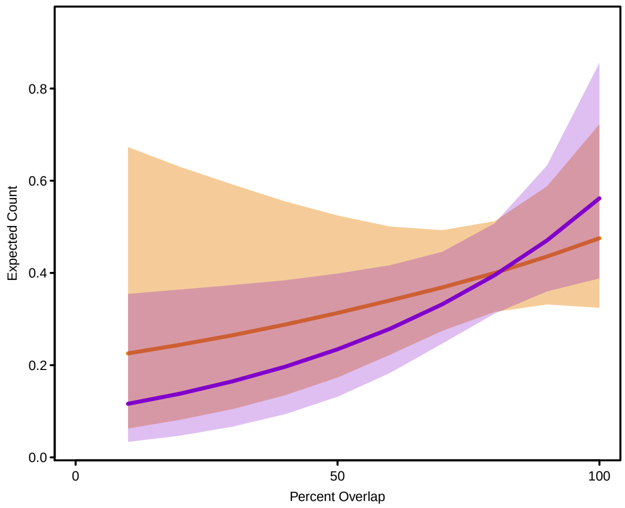

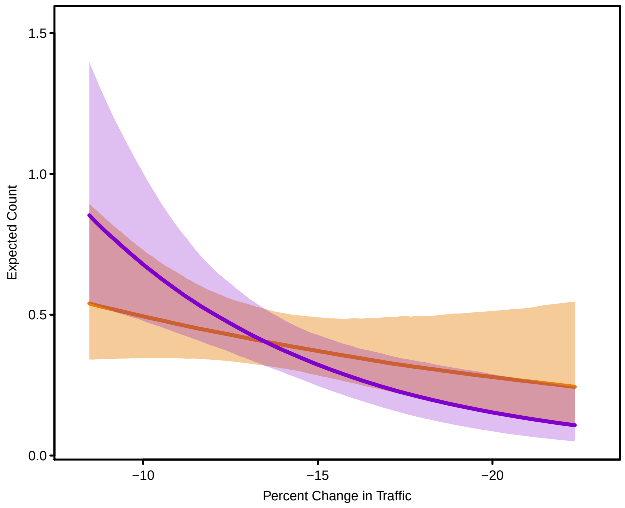

posterior distribution 95% CI
for interaction with
land includes zero

posterior distribution 95% CI
for interaction with
air includes zero

posterior distribution 95% CI
for interaction with
road includes zero

Turkey Vulture

Cathartes aura

posterior distribution 95% CI
for interaction with
overlap includes zero

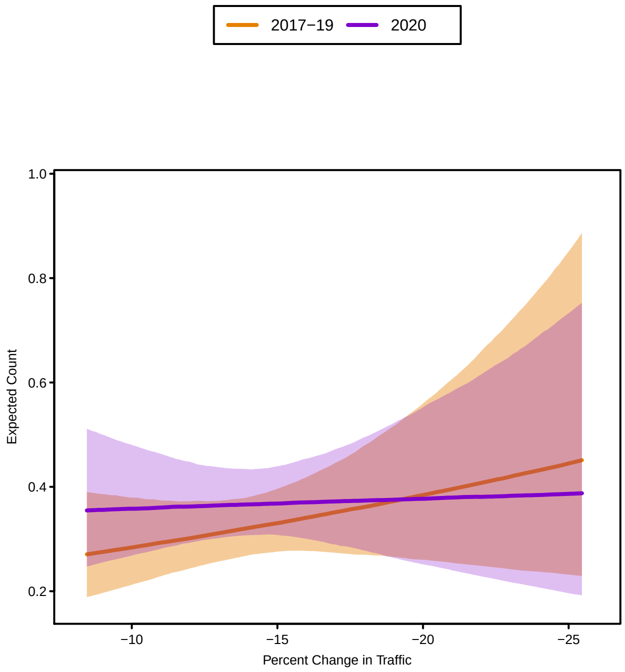

posterior distribution 95% CI
for interaction with
land includes zero

posterior distribution 95% CI
for interaction with
air includes zero

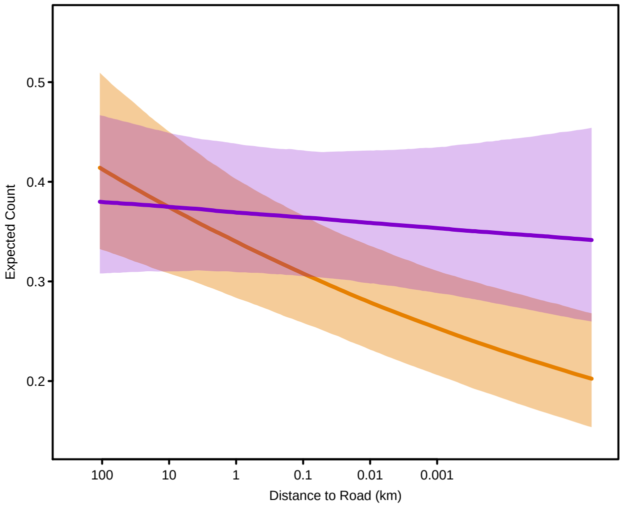

Warbling Vireo

Vireo gilvus

2017−19 2020

posterior distribution 95% CI
for interaction with
overlap includes zero

posterior distribution 95% CI
for interaction with
delta_traf includes zero

posterior distribution 95% CI
for interaction with
land includes zero

posterior distribution 95% CI
for interaction with
air includes zero

posterior distribution 95% CI
for interaction with
road includes zero

White−breasted Nuthatch

Sitta carolinensis

2017−19 2020

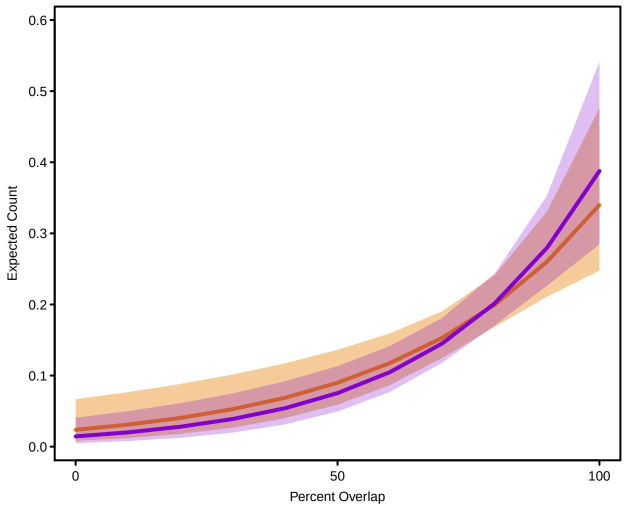

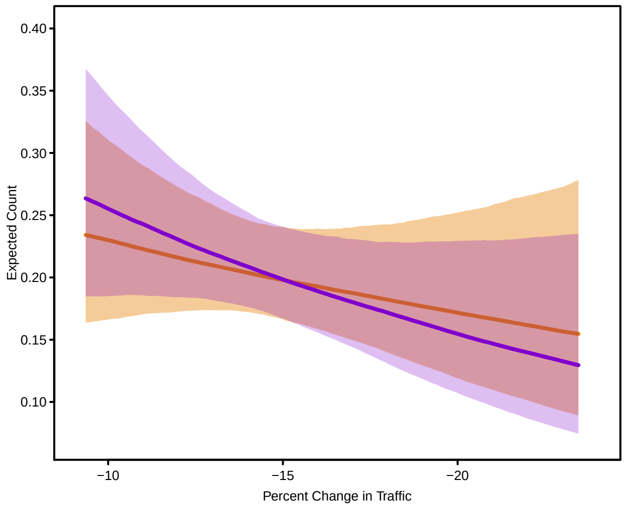

posterior distribution 95% CI
for interaction with
land includes zero

posterior distribution 95% CI
for interaction with
air includes zero

posterior distribution 95% CI
for interaction with
road includes zero

White−crowned Sparrow

Zonotrichia leucophrys

posterior distribution 95% CI
for interaction with
overlap includes zero

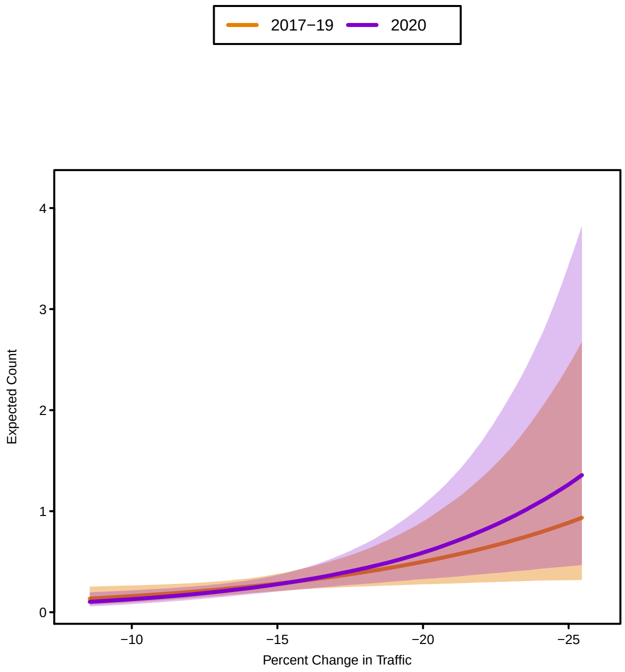

posterior distribution 95% CI
for interaction with
land includes zero

posterior distribution 95% CI
for interaction with
air includes zero

posterior distribution 95% CI
for interaction with
road includes zero

White−eyed Vireo

Vireo griseus

posterior distribution 95% CI
for interaction with
overlap includes zero

2017−19 2020

posterior distribution 95% CI
for interaction with
delta_traf includes zero

posterior distribution 95% CI
for interaction with
land includes zero

posterior distribution 95% CI
for interaction with
air includes zero

posterior distribution 95% CI
for interaction with
road includes zero

White−throated Sparrow

Zonotrichia albicollis

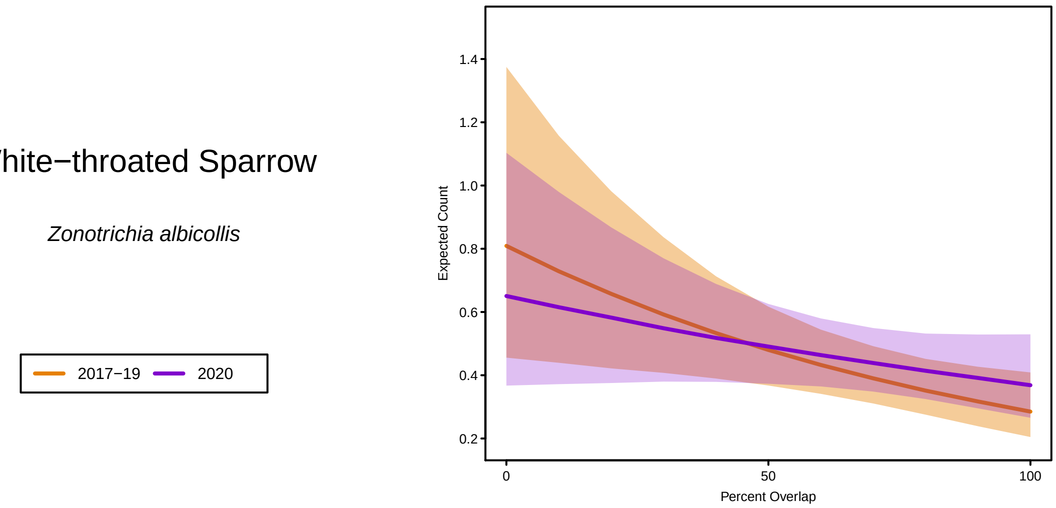

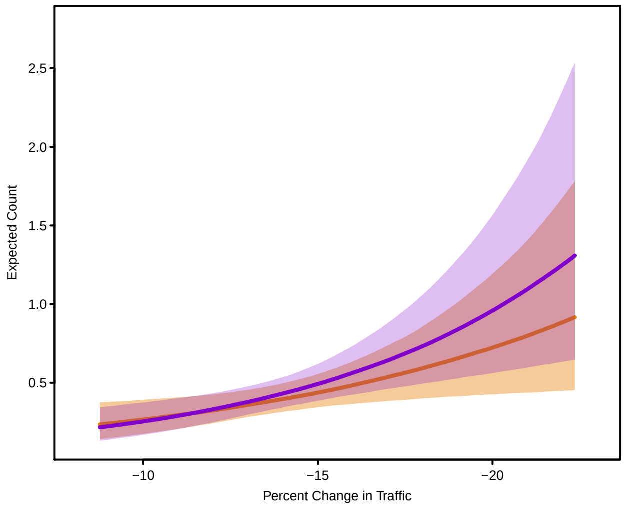

posterior distribution 95% CI
for interaction with
land includes zero

posterior distribution 95% CI
for interaction with
air includes zero

posterior distribution 95% CI
for interaction with
road includes zero

Wood Duck

Aix sponsa

posterior distribution 95% CI
for interaction with
overlap includes zero

posterior distribution 95% CI
for interaction with
delta_traf includes zero

posterior distribution 95% CI
for interaction with
land includes zero

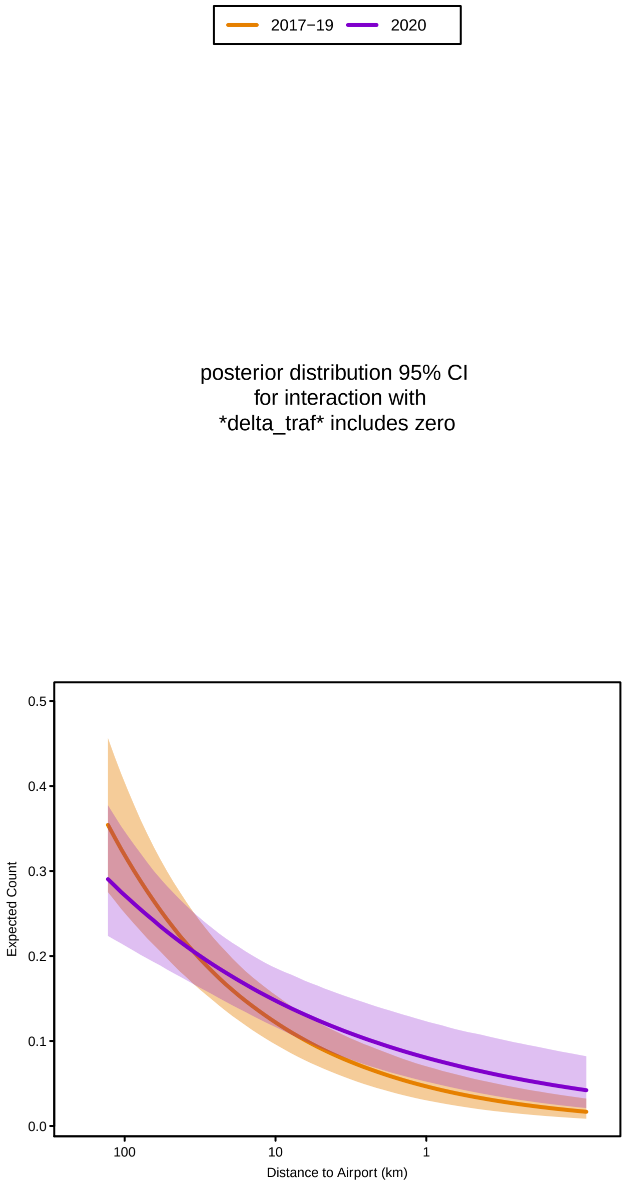

posterior distribution 95% CI
for interaction with
road includes zero

Yellow−rumped Warbler

Setophaga coronata

2017−19 2020

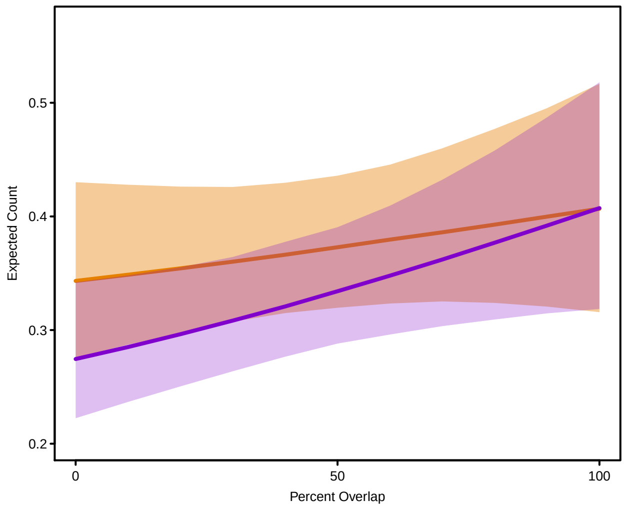

posterior distribution 95% CI
for interaction with
delta_traf includes zero

posterior distribution 95% CI
for interaction with
land includes zero

posterior distribution 95% CI
for interaction with
air includes zero

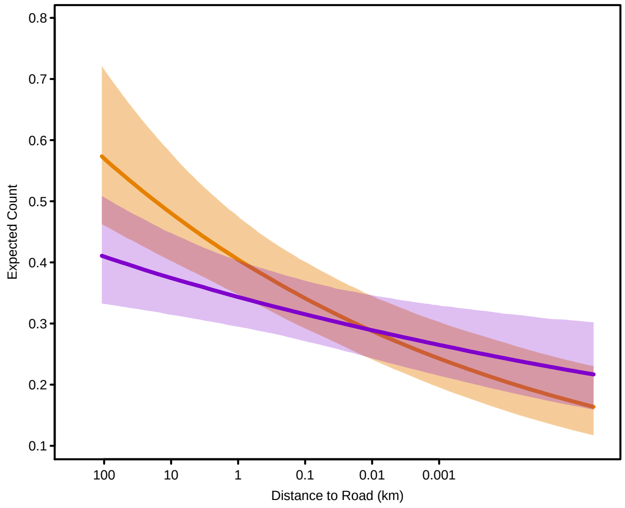

Yellow Warbler

Setophaga petechia

posterior distribution 95% CI
for interaction with
overlap includes zero

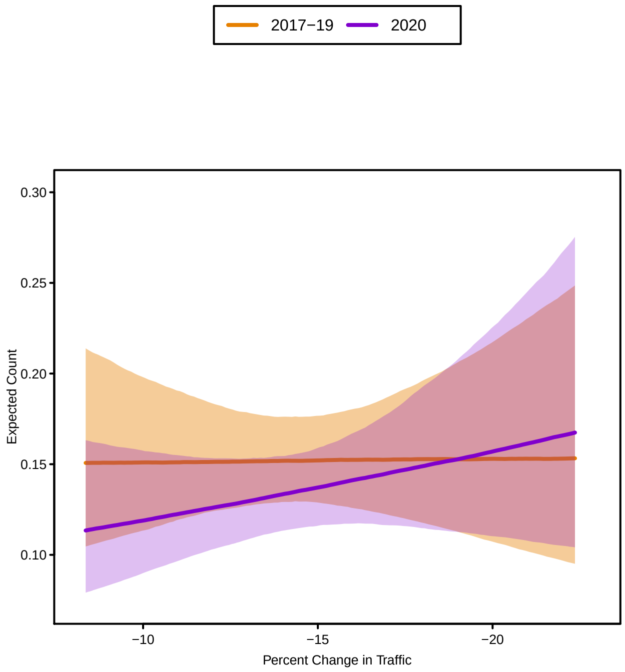

posterior distribution 95% CI
for interaction with
land includes zero

posterior distribution 95% CI
for interaction with
air includes zero

posterior distribution 95% CI
for interaction with
road includes zero

Table S1.
Summary statistics for variables used in the modeling process. Note that distances to airports and roads were logged for modeling (see Figs. S3-S4).

Variable	Type	Range	Mean (S.D)
County	Categorical, random	*n.a.*	*n.a.*
Pandemic	Categorical	Pre-, During-pandemic	*n.a.*
Distance (km)	Continuous	[0, 3]	0.757 (0.88)
Duration (min)	Continuous	[1, 300]	48.7 (47.5)
Change in Traffic (%)	Continuous, county specific	[-25.4, -8.37]	-14.3 (3.17)
Overlap (%)	Continuous, county specific	[0, 100]	65.2 (25.5)
Distance to airport (km)	Continuous	[0.09, 292]	35.0 (19.3)
Distance to road (km)	Continuous	(0, 98.2]	0.775 (2.18)
Landcover	Categorical	Urban, Rural	*n.a.*

Table S2.
Contingency table of the number of checklists (in all counties) that came from urban vs. rural habitat in each time period. Pearson's Chi-squared test with Yates' continuity correction ($\chi^2 = 0.0219, df = 1, p = 0.8824$).

Period	Urban	Rural
2017-2019	23064	21239
2020	23041	21262

Table S3.
Detectability values used for each species, calculations sensu (*46*), with data from the Canadian Wildlife Service and (*49*).

Species	Detectability
American crow	0.619
American goldfinch	0.186
American redstart	0.255
American robin	0.451
Baltimore oriole	0.357
barn swallow	0.288
black-and-white warbler	0.249
black-capped chickadee	0.240
belted kingfisher	0.180
brown-headed cowbird	0.303
blue jay	0.398
brown thrasher	0.533
blue-winged teal	0.440
Canada goose	0.371
cedar waxwing	0.159
chipping sparrow	0.323
common grackle	0.384
common yellowthroat	0.413
dark-eyed junco	0.275
downy woodpecker	0.199
eastern bluebird	0.165
eastern kingbird	0.370
eastern phoebe	0.356
eastern towhee	0.457
European starling	0.534
great crested flycatcher	0.391
gray catbird	0.277

Species	Detectability
hairy woodpecker	0.217
house sparrow	0.474
house wren	0.595
indigo bunting	0.406
killdeer	0.430
mallard	0.455
mourning dove	0.571
Nashville warbler	0.406
northern flicker	0.307
northern parula	0.327
orange-crowned warbler	0.234
palm warbler	0.278
pileated woodpecker	0.355
rose-breasted grosbeak	0.413
ruby-crowned kinglet	0.375
red-eyed vireo	0.520
ruby-throated hummingbird	0.055
red-winged blackbird	0.546
song sparrow	0.446
Swainson's thrush	0.430
tree swallow	0.406
warbling vireo	0.373
white-breasted nuthatch	0.265
white-crowned sparrow	0.490
white-throated sparrow	0.460
yellow warbler	0.388
yellow-rumped warbler	0.251

Table S4.
Impacts of detectability on the probability of species showing increases or any change in counts relative to lower human activity during the pandemic period (*n* = 54 species). LCL = lower 95% confidence limit, HCL = upper 95% confidence limit.

Variable	*β*	SE	*p*	LCL	HCL
Were less detectable species more likely to show increased counts in areas with decreased human activity?					
Urban-rural	-1.607	2.729	0.556	-6.955	3.741
Distance to airport	6.416	7.915	0.418	-9.097	21.930
Distance to road	-0.119	2.806	0.966	-5.619	5.381
Change in traffic	0.269	2.992	0.928	-5.595	6.132
Overlap migration-travel restrictions	-1.207	2.280	0.597	-5.675	3.261
Were less detectable species more likely to show any change in counts in areas with decreased human activity?					
Urban-rural	-3.803	2.563	0.138	-8.826	1.220
Distance to airport	4.317	3.417	0.206	-2.379	11.014
Distance to road	1.082	2.546	0.671	-3.908	6.071
Change in traffic	0.557	2.505	0.824	-4.352	5.466
Overlap migration-travel restrictions	-0.350	2.322	0.880	-4.902	4.201

Table S5.

Impacts of mean species body size on the probability of species showing increases or any change in counts relative to lower human activity during the pandemic period (n = 82 species). LCL = lower 95% confidence limit, HCL = upper 95% confidence limit.

Variable	β	SE	p	LCL	HCL
Were smaller species more likely to show increased counts in areas with decreased human activity?					
Urban-rural	0.003	0.175	0.987	-0.339	0.345
Distance to airport	0.357	0.152	0.019	0.059	0.656
Distance to road	0.090	0.152	0.553	-0.208	0.388
Change in traffic	-0.071	0.201	0.722	-0.464	0.322
Overlap migration-travel restrictions	-0.156	0.132	0.238	-0.414	0.103
Were smaller species more likely to show any change in counts in areas with decreased human activity?					
Urban-rural	0.069	0.135	0.612	-0.197	0.334
Distance to airport	0.289	0.145	0.047	0.004	0.574
Distance to road	0.101	0.140	0.472	-0.174	0.375
Change in traffic	-0.005	0.162	0.978	-0.322	0.313
Overlap migration-travel restrictions	-0.159	0.129	0.219	-0.412	0.094

Data S1. (separate file)
A list of all counties included in the modeling process with associated metadata.

Columns include:
County = county name
eBird_county_code = county code used in the eBird database
major_city = name of the major city located in the county
Country = country name
eBird_country_code = country code used in the eBird database
State = state/province name
eBird_state_code = state/province code used in the eBird database
latitude = latitude of a point in the center of the checklists from that county
longitude longitude of a point in the center of the checklists from that county

Data S2. (separate file)
A list of all species included in the study with associated metadata.

Columns include:
eBird_species_code = 6-character taxonomic code used in the eBird database
common_name = English common name
scientific_name = scientific name
family = taxonomic family
band_code = 4-character banding code
notes = additional notes

Data S3. (separate file)
Comma separated value file with posterior summary statistics and convergence diagnostics for all modeled covariates. Column headers are constructed with abbreviations for *variable name* [*hyphen*] *statistic name*.

Variable names refer to:
dist = distance traveled
dur = duration
pandemic = main effect of pandemic (pre- vs post-pandemic)
overlap = main effect of overlap between peak traffic reduction and peak bird counts
delta_traf = main effect of traffic reduction
air = main effect of logged distance from airport
road = main effect of logged distance from road
land = main effect of local landcover (urban vs rural)
pandemic:overlap = interaction between pandemic and overlap
pandemic:delta_traf = interaction between pandemic and delta_traf
pandemic:air = interaction between pandemic and air
pandemic:road = interaction between pandemic and road
pandemic:land = interaction between pandemic and land

Statistic names refer to:
mean = mean
sd = standard deviation
lower = 2.5% quantile, i.e., lower limit of 95% credible interval
median = median, i.e. 50% quantile
upper = 97.5% quantile, i.e., upper limit of 95% credible interval
Rhat = $\hat{R}$ value, used to check convergence
ESS = crude measure of effective sample size of the posterior

Data S4. (separate file)

R script file containing code used to extract and filter eBird data, prepare individual species data files, run each single-species model using the rstanarm interface with the Stan language, and interpret results.
